# Expanding the genetic toolbox of *Rhodotorula toruloides* by identification and validation of six novel promoters induced or repressed under nitrogen starvation

**DOI:** 10.1186/s12934-023-02175-2

**Published:** 2023-08-19

**Authors:** Daniel P. Brink, Friederike Mierke, Joakim Norbeck, Verena Siewers, Thomas Andlid

**Affiliations:** 1 Systems and Synthetic Biology, Department of Life Sciences, Chalmers University of Technology, Gothenburg, Sweden; 2 Applied Microbiology, Department of Chemistry, Lund University, Lund, Sweden; 3 Food and Nutrition Science, Department of Life Sciences, Chalmers University of Technology, Gothenburg, Sweden; 4 The Novo Nordisk Foundation Center for Biosustainability, Technical University of Denmark, Lyngby, Denmark

**Keywords:** *Rhodotorula toruloides*, *Rhodosporidium*, MinION, RNAseq, Differential gene expression, Nitrogen starvation, Glucose, Xylose, Promoters

## Abstract

**Background:**

The non-conventional yeast *Rhodotorula toruloides* is an emerging host organism in biotechnology by merit of its natural capacity to accumulate high levels of carotenoids and intracellular storage lipids from a variety of carbon sources. While the number of genetic engineering strategies that employ *R. toruloides* is increasing, the lack of genetic tools available for modification of this yeast is still limiting strain development. For instance, several strong, constitutive *R. toruloides* promoters have been characterized, but to date, only five inducible promoters have been identified. Although nitrogen-limited cultivation conditions are commonly used to induce lipid accumulation in this yeast, no promoters regulated by nitrogen starvation have been described for* R. toruloides.*

**Results:**

In this study, we used a combination of genomics and transcriptomics methods to identify novel *R. toruloides* promoter sequences that are either inducible or repressible by nitrogen starvation. RNA sequencing was used to assess gene expression in the recently isolated strain *R. toruloides* BOT-A2 during exponential growth and during nitrogen starvation, when cultivated with either glucose or xylose as the carbon source. The genome of BOT-A2 was sequenced using a combination of long- and short-read sequencing and annotated with support of the RNAseq data. Differential expression analysis was used to identify genes with a |log_2_ fold change|≥ 2 when comparing their expression during nitrogen depletion to that during exponential growth. The promoter regions from 16 of these genes were evaluated for their ability to drive the expression of a fluorescent reporter gene. Three promoters that were clearly upregulated under nitrogen starvation and three that were downregulated were selected and further characterized. One promoter, derived from gene RTBOTA2_003877, was found to function like an on–off switch, as it was only upregulated under full nitrogen depletion and downregulated in the presence of the nitrogen source.

**Conclusions:**

Six new *R. toruloides* promoters that were either upregulated or downregulated under nitrogen-starvation were identified. These substantially contribute to the available promoters when engineering this organism and are foreseen to be particularly useful for future engineering strategies requiring specific regulation of target genes in accordance with nitrogen availability.

**Supplementary Information:**

The online version contains supplementary material available at 10.1186/s12934-023-02175-2.

## Background

The basidiomycete yeast *Rhodotorula toruloides*, also known as *Rhodosporidium toruloides* [1], has emerged as a promising cell factory for biological production of lipids, carotenoids, and certain industrially relevant enzymes [2–4]. It is capable of accumulating high levels of internal storage lipids in the form of triacylglycerols (TAGs) from excess carbon when other nutrients such as nitrogen, phosphorous or sulphur are limited [5–7]. Its natural capacity to grow on a variety of carbon sources, including several hexose and pentose sugars, glycerol, acetate, plant oils, and certain aromatic compounds [8–10], together with its inherent tolerance to some inhibitory compounds, e.g. formic and acetic acid [11–13], make it an attractive microbe for bioconversion of renewable feedstocks, such as lignocellulosic hydrolysates and other waste streams. *R. toruloides* can utilise a variety of nitrogen sources including ammonium sulphate, ammonium nitrate, urate and urea. However, the central nitrogen metabolic pathway in *R. toruloides* uses ammonia as an input, while an existing alternative nitrogen source metabolism for urate and urea is needed for further use of those nitrogen sources [14]. Thanks to major achievements in method development in the last decade, several successful strategies for engineering *R. toruloides* for improved production of native and non-native products have been demonstrated [15–21].

Nevertheless, the genetic engineering tools of *R. toruloides* are still very limited compared to those available for the model yeast *Saccharomyces cerevisiae* and fellow oleaginous yeast *Yarrowia lipolytica*. Development of efficient genetic tools for engineering of *R. toruloides* is therefore going to be a crucial driver for the advancement of this yeast species as an industrially relevant cell factory. The *R. toruloides* genetic toolbox currently contains methods for transformation [22–25], CRISPR-Cas9 tools [26–28], in silico models [29–32], and a growing number of functionally verified promoters and terminators for control of gene expression [2]. As is the case for many microbes, a majority of the currently utilized promoters for engineering *R. toruloides* are constitutive and many, but not all, are derived from glycolytic genes [22, 33–36]. Although constitutive promoters are essential components of many recombinant gene expression strategies, there also exists a need to identify inducible and repressible promoters in order to be able to create more complex gene expression circuits and bioprocess designs. In particular, we see a need to identify *R. toruloides* promoters that are differentially active during lipid-accumulation conditions, including limitation or depletion of nitrogen. Nitrogen starvation-induced promoters could for example be used to increase lipid production and lipid yields, and promoters that are repressed during nitrogen starvation could be used to reduce lipid production and potentially channel the acetyl-CoA flux towards other bioproducts such as carotenoids.

To our knowledge, five inducible *R. toruloides* promoters have been identified to date. Liu et al. characterized the *DAO1* promoter and found it to be strongly induced by availability of d-amino acids as a carbon source [37]. Johns and colleagues identified promoters from *ICL1, CTR3, MET16*, and *NAR1* in *R. toruloides* CBS14 based on homology to functioning promoters in other fungi: *ICL1p* was induced by switching the carbon source from glucose to sodium acetate, *CTR3p* by copper deficiency, *MET16p* by methionine deficiency, and *NAR1p* by a combination of ammonium starvation and presence of nitrate [38]. *NAR1p* is an example of an inducible promoter that responds to nitrogen availability, but since it is induced by nitrate presence, it cannot be used to induce gene expression during nitrogen-depletion.

In this study we used transcriptomics to identify putative *R. toruloides* promoters that are either upregulated or downregulated under limitation and subsequent depletion of the nitrogen source ammonium sulphate. From here on, all occasions of nitrogen depletion, starvation or limitation in this study refer to ammonium sulphate depletion, starvation and limitation. Several *R. toruloides* transcriptome datasets are available from a variety of different culture conditions, including nitrogen limitation [14, 39], phosphate limitation [5], stress response during growth on hydrolysates [11, 40, 41] and growth on different carbon sources [10, 30, 33, 39, 42], but no study has yet compared the effect of hexose and pentose sugars during nitrogen starvation. We here generated a highly contiguous, functionally annotated genome assembly from our recently isolated *Rhodotorula toruloides* strain BOT-A2 (previously classified as *Rhodosporidium toruloides*) [43] and performed RNA sequencing (RNAseq) on samples cultivated in nitrogen-limited media with either glucose or xylose as the sole carbon source. Differential expression analysis of samples taken during the exponential growth phase and at nitrogen depletion was used to identify putative promoter candidate sequences, which were further evaluated using reporter gene expression.

## Results

### Genome and transcriptome analysis of *R. toruloides* BOT-A2

#### Genome assembly and average nucleotide identity analysis

**Fig. 1:**
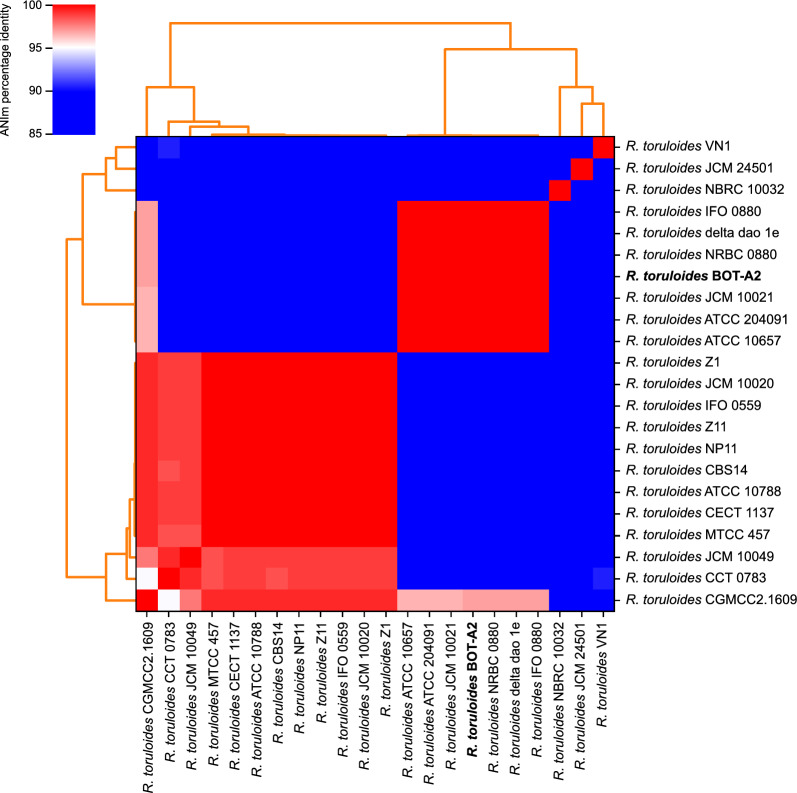
Average Nucleotide Identity (ANI) using Mummer as an aligner (ANIm). Comparison of BOT-A2 with all other available *R. toruloides* strains that have published genomes on NCBI. > 95% ANI percent identity is commonly considered as cut-off for species delineation [109] and is represented by the red squares in the heat map. White squares: 95% percent identity. Blue squares < 95% percent identity

As a basis for the RNAseq analysis, the genome of *R. toruloides* BOT-A2 was sequenced using a combination of long-read and short-read sequencing methods. This resulted in one of the most contiguous *R. toruloides* assemblies to date, consisting of 20 contigs and a total genome size of 20.54 Mb (Table 1). Based on observations from previous studies that *R. toruloides* genome sequences differ substantially between haploid strains with different mating types [44, 45], an average nucleotide identity (ANI) analysis was conducted using the genomes from all strains with public genome assemblies available on NCBI, and the polished BOT-A2 assembly. The analysis revealed two major clusters with different genomic nucleotide composition as illustrated in the ANI heatmap (Fig. 1). Using protein sequences from previously identified *R. toruloides* mating pheromone receptors A1 and A2 (Uniprot: M7X934 and G0T0M8), it was found that all genomes in the top ANI cluster (Fig. 1) contained the receptor locus for the A1 mating type. The bottom cluster contained genomes that all contained the receptor locus for the A2 mating type (Additional file 1: Table S1). It is worth noting that mating type switching of the kind that occurs in *S. cerevisiae* has been suggested not to occur in *R. toruloides* [46]. All strains only had one clear pheromone receptor type homolog (A1 or A2) in their genome assemblies, with the exception of CCT 0783, which seemed to contain both loci and thus suggests that this is a diploid strain. Strain BOT-A2, named unrelated to its mating type, was found to cluster in the MAT A2 cluster together with strains such as NRBC 0880 (Fig. 1). Strains such as NP11 and CBS14 were placed in the MAT A1 cluster (Fig. 1). We furthermore identified three *R. toruloides* strains that did not cluster with any of the two larger clusters at all: *R. toruloides* VN1, *toruloides* NBRC 10032, and *R. toruloides* JCM 24501. The two former contained homologs for the A1 pheromone receptor, and the latter for type A2 (Additional file 1: Table S1). The *R. toruloides* CGMCC2.1609 strain that clustered in both ANI clusters (Fig. 1), only had a locus with homology to the A1 pheromone receptor, and not to the A2 receptor (Additional file 1: Table S1).

**Table 1:** Metrics of the final BOT-A2 assembly, compared to the assembly of the closely related strain NRBC 0880 and the current most contiguous *R. toruloides* assembly Delta dao 1e

*R. toruloides* strain	BOT-A2	NRBC 0880	Delta dao 1e
Number of contigs	20	30	19
Largest contig [bp]	2,201,149	2,216,085	3,763,927
Contigs ≥ 50,000 bp	19	18	19
Total length [Mb]	20.54	20.68	21.18
GC content [%]	61.77	61.83	61.49
N50 [bp]	1,308,578	1,390,799	1,460,187
N75 [bp]	938,637	870,210	954,776
L50	7	6	5
L75	12	11	10
# N’s per 100 kbp	0.00	0.00	195.33
NCBI Accession number	JAOEGQ000000000	GCA_000988875.2	GCA_023968905.1

#### RNA sequencing and genome annotation

**Fig. 2:**
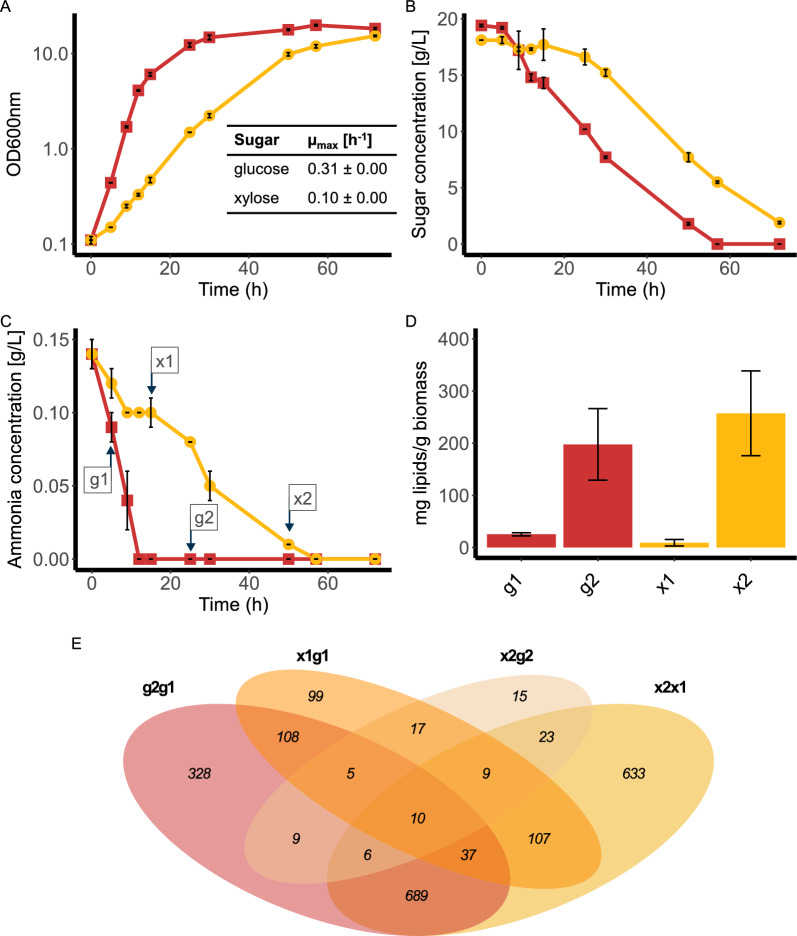
Differential gene expression analysis during shake flask cultivations of BOT-A2 on glucose and xylose. Parameters measured include growth as OD_600nm_ **A**, sugar concentration for glucose or xylose **B**, ammonia concentration **C**, and lipid concentration in mg lipids/g CDW at growth on glucose and on xylose **D**. All experiments were performed in biological triplicates and error bars represent the standard deviations. RNAseq sampling time points are shown in C. RNAseq samples were taken at two time points with g1 = time point 1 during exponential growth on glucose, x1 = time point 1 during exponential growth on xylose, and g2 = time point 2 during nitrogen-starvation phase on glucose, and x2 = time point 2 during nitrogen-starvation phase on xylose. Red and ■ = BOT-A2 on glucose, Yellow and ● = BOT-A2 on xylose. The Venn diagram (E) illustrates all 2095 significantly differentially expressed genes (fulfilling cut-off values of |log_2_ fold change|≥ 2 and adjusted p-value ≤ 10^–6^) identified in the four different comparisons: g2g1—differential gene expression during nitrogen starvation on glucose; x2x1—differential gene expression during nitrogen starvation on xylose; x1g1—differential gene expression during exponential growth on xylose compared to glucose; x2g2—differential gene expression during nitrogen-starvation on xylose compared to glucose

To generate samples for RNAseq, BOT-A2 was cultivated in shake flasks in CN80 medium with glucose or xylose as the carbon source (Fig. 2). Samples were taken at two distinct time points on either sugar according to ammonia and biomass levels: g1–exponential growth on glucose, x1–exponential growth on xylose, g2–nitrogen-starved cultivation on glucose, x2–nitrogen-starved cultivation on xylose. The growth rate for BOT-A2 was three times higher on glucose with 0.31 ± 0.00 h^−1^ compared to 0.10 ± 0.00 h^−1^ on xylose, and sugar and ammonia consumption varied accordingly. Because of the differences in growth rate, the specific time points at which the samples were collected differed between the two sugars. Glucose was completely depleted after 57 h, while residual xylose remained in the cultivation even after 72 h. Ammonia was completely depleted after 12 h during growth on glucose, and after 57 h for growth on xylose. Total acyl lipid analysis (Fig. 2D) confirmed that the cells had entered the lipid accumulation phase at time point 2 compared to time point 1 on both sugars. A total of 12 RNAseq samples were collected: biological triplicates of two different time points on two different sugars.

Using the RNAseq reads as biological evidence, a pipeline for annotation of the genome assembly was run. A total of 7001 genes were predicted in the final gene model, including rRNA, mRNA and tRNA genes (Additional file 1: Table S2). The predicted genes were converted to their corresponding amino acid sequences and were functionally annotated based on homology to known proteins (Additional file 2: Fig. S2). The sequence reads from the 12 RNAseq samples were mapped to the annotated assembly, and a principal component analysis (PCA) was performed to evaluate the robustness of the RNAseq experimental setup. The samples indeed clustered in four different groups representing each of the different cultivation conditions: g1, g2, x1, x2 (Additional file 1: Fig. S1).

#### Differential gene expression analysis

Differential expression (DE) values for each predicted gene were calculated using DESeq2 for four different comparisons: nitrogen starvation versus exponential growth on glucose (g2g1) and on xylose (x2x1); exponential growth on xylose compared to glucose (x1g1); and nitrogen-starvation on xylose compared to glucose (x2g2). Across all 12 samples, a total of 2095 genes were found to be significantly differentially expressed (Benjamini-Hochberg-adjusted p-value < 10^–6^) while also fulfilling the designated fold change threshold of |log_2_ fold change|≥ 2 (Fig. 2E). Most of those genes were identified in the two nitrogen starvation conditions (g2g1: 1192 DE genes; x2x1: 1514 DE genes). When comparing expression levels during growth on glucose versus xylose during time point 1 (x1g1) 392 DE genes were identified, but only 94 DE genes were found when comparing the samples from the two different sugars at time point 2 (x2g2). A master table containing the raw and processed data from the RNAseq experiment for all predicted BOT-A2 genes is available in Additional file 2.

**Fig. 3:**
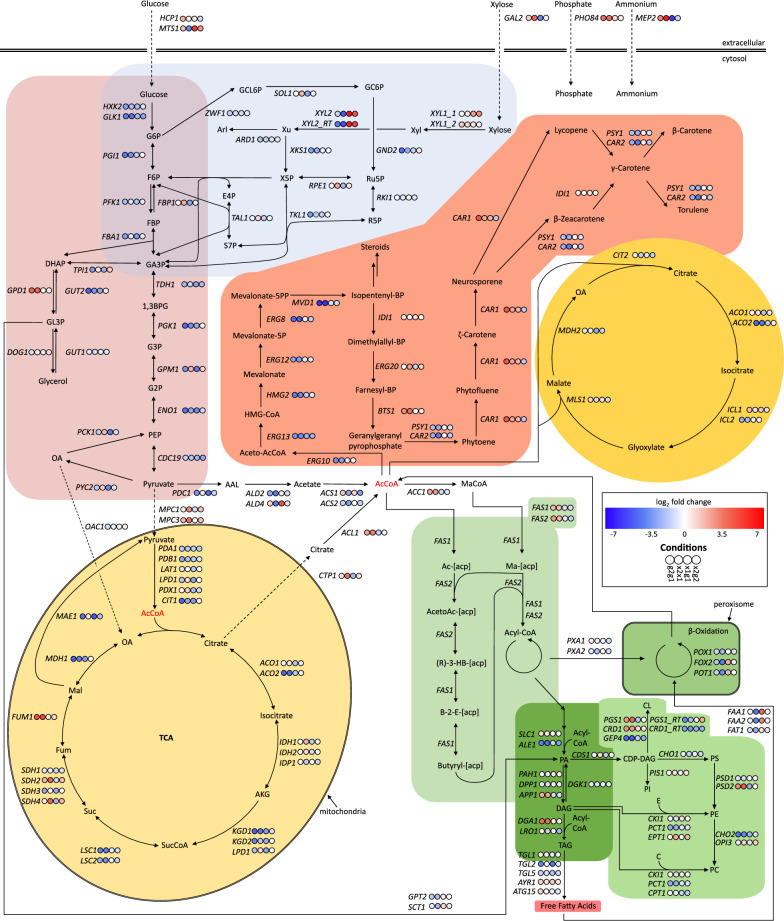
Map of central metabolic pathways in *R. toruloides* BOT-A2. Pathways were reconstructed from genome and RNAseq data, using KEGG, SGD and BLASTp. The network includes glycolysis (pink), the pentose phosphate pathway (blue), the TCA cycle (pale yellow), lipid biosynthesis, triacylglycerol biosynthesis and β-oxidation (all different shades of green), as well as carotenoid biosynthesis (orange), the glyoxylate cycle (dark yellow), and specific transport processes. Results from differential gene expression analysis are displayed for all 4 conditions analysed, conditions are as follows: g2g1—differential gene expression during nitrogen starvation on glucose; x2x1—differential gene expression during nitrogen starvation on xylose; x1g1—differential gene expression during exponential growth on xylose compared to glucose; x2g2—differential gene expression during nitrogen-starvation on xylose compared to glucose. Compound abbreviations: G6P: D-glucose-6P, F6P: D-fructose-6P, FBP: D-fructose-1,6P2, DHAP: dihydroxyacetone phosphate, GL3P: glycerol-3P, GA3P: D-glyceraldehyde-3P, 1,3BPG: glycerate-1,3P2, G3P: glycerate-3P, G2P: glycerate-2P, PEP: phosphoenolpyruvate, OA: oxaloacetate, AKG: α-ketoglutarate, SucCoA: succinyl-CoA, Suc: succinate, Fum: fumarate, Mal: (S)-malate, AAL: acetaldehyde, AcCoA: acetyl-CoA, MaCoA: malonyl-CoA, GCL6P: D-glucono-1,5-lactone-6P, GC6P: D-gluconate-6P, Xyl: xylitol, Xu: D-xylulose, Ru5P: D-ribulose-5P, R5P: D-ribose-5P, X5P: D-xylulose-5P, Arl: arabinitol, E4P: D-erythrose-4P, S7P: D-sedoheptulose-7P, Ac-[acp]: acetyl-[acp], Ma-[acp]: malonyl-[acp], AcetoAc-[acp]: acetoacetyl-[acp], (R)-3-HB-[acp]: (R)-3-hydroxybutanoyl-[acp], B-2-E-[acp]: but-2-enoyl-[acp], PA: phosphatidic acid, DAG: diacylglycerol, TAG: triacylglycerol. Gene name abbreviations are according to SGD or other oleaginous yeasts

Several central metabolic pathways were reconstructed for *R. toruloides* BOT-A2 based on the functional annotation and the RNAseq results (Fig. 3). In general, in the reconstructed metabolic network (Fig. 3), around a third of the genes were significantly differentially expressed while also having a |log_2_ fold change|≥ 2 during nitrogen starvation. The putative fatty acid synthesis genes *FAS1* (RTBOTA2_004415) and *FAS2* (RTBOTA2_004570) were significantly differentially expressed during nitrogen starvation but did not fulfil the additional threshold of |log_2_ fold change|≥ 2. The only highly upregulated DE gene fulfilling the fold change threshold in the lipid pathway was the putative diacylglycerol acyltransferase gene *DGA1* (RTBOTA2_000438)*,* which is related to the formation of TAGs. A few genes related to xylose metabolism were highly upregulated during growth on xylose (x1g1, x2g2), specifically the two putative D-xylulose reductases/xylitol dehydrogenase genes *XYL2* (RTBOTA2_004775) and *XYL2_RT* (RTBOTA2_000431).

#### Selection of promoter candidates

Using the differential expression analysis data, 15 genes potentially regulated by nitrogen-levels were selected for further characterization of the inducible or repressible nature of their promoters (Table 2). Only significantly differentially expressed genes fulfilling the additional |log_2_ fold change|≥ 2 threshold for both g2g1 and x2x1 were selected. No genes annotated as *hypothetical protein* were considered since there is a risk these might represent erroneous gene predictions that for instance might lack some exons. Putative promoter sequences from the selected genes were identified by selecting the DNA sequence directly upstream of the start codon; the final promoter lengths varied from promoter to promoter based on the distance to the closest upstream gene, with lengths being in the range of 600 bp to 1000 bp. The final sequences are available in Additional file 3. In addition to the nine upregulated candidates selected from the BOT-A2 transcriptome data, the promoter from the *MEP2* gene from the *R. toruloides* strain NP11 was also chosen by merit of its high upregulation during nitrogen-limited conditions [14]. While the RNAseq sampling conditions as well as normalization methods are not comparable between our analysis and previously conducted analyses, we were still interested in how the promoter would work in BOT-A2 despite the genomic differences between BOT-A2 and NP11 that were uncovered (Fig. 1). Interestingly, the BOT-A2 *MEP2* gene did not fulfil all the promoter selection criteria in our analysis: while the adjusted p-value threshold was fulfilled, the fold change after nitrogen limitation was 1.7 for g2g1 and 5.9 for x2x1. The *MEP2* promoter sequence was identified in the NP11 genome assembly in the same way as previously described for the BOT-A2 promoters.

**Table 2:** BOT-A2 promoter table with DE number

Promoter name	Gene annotation	Simplified strain names	Glucose				Xylose			
			Counts_norm_ t1	Counts_norm_ t2	Log2 FC t2 vs t1	p-value t2 vs t1	Counts_norm_ t1	Counts_norm_ t2	Log2 FC t2 vs t1	p-value t2 vs t1
3877	Nitrate transporter	UP1a–UP1e	5.8 ± 0.6	1963.6 ± 161.6	8.4	0	3.5 ± 0.4	619.3 ± 9.9	7.7	0
4847	Alcohol oxidase Fao1p	UP2a–UP2e	32.4 ± 3.0	608.1 ± 91.7	4.2	9.5e-83	14.9 ± 3.5	281.5 ± 44.1	4.5	1.6e-89
0480	Carboxypeptidase Y inhibitor Tfs1p	UP3a–UP3e	8.0 ± 2.6	310.4 ± 14.4	5.3	2.5e-75	80.1 ± 12.8	444.4 ± 22.3	2.7	4.1e-22
2184	Protein yippee-like Moh1p	UP4a–UP4e	36.9 ± 2.8	547.3 ± 112.0	3.9	5.3e-49	70.4 ± 13.1	830.9 ± 143.4	3.8	5.0e-47
6908	Autophagy-related protein	UP5a–UP5e	281.2 ± 95.5	2580.8 ± 215.5	3.2	5.4e-25	780.5 ± 159.9	4270.3 ± 697.5	2.7	3.5e-18
2391	Alcohol dehydrogenase	UP6a–UP6e	14.9 ± 2.7	282.8 ± 27.7	4.2	1.1e-55	23.2 ± 5.6	386.8 ± 47.8	4.3	2.5e-57
6709	Fatty acid elongation protein	UP7a–UP7e	182.2 ± 78.5	1227.2 ± 83.6	2.7	4.6e-19	89.4 ± 18.1	1371.3 ± 24.1	4.2	1.4e-42
0568	Protein Orm1p	UP8a–UP8e	42.8 ± 9.6	282.1 ± 11.6	2.7	8.8e-23	65.9 ± 16.8	335.7 ± 14.3	2.6	9.8e-21
5111	Dehydrogenase/reductase	UP9a-UP9e	75.9 ± 11.3	463.6 ± 35.7	2.6	4.7e-24	14.9 ± 2.9	442.8 ± 90.8	5.1	8.7e-88
0530	Ribosome-associated molecular chaperone Ssb2p	DN1a–DN1e	297.1 ± 29.8	6.0 ± 0.8	-5.6	1.6e-57	185.9 ± 80.3	3.6 ± 1.4	-5.4	3.0e-51
3356	Diphosphomevalonate decarboxylase Mvd1p	DN2a–DN2e	25.1 ± 1.9	0.7 ± 0.2	-5.2	9.9e-50	21.2 ± 4.9	0.4 ± 0.3	-5.4	1.3e-38
5449	Epsin-3 Ent3p	DN3a–DN3e	9.5 ± 0.6	0.6 ± 0.1	-4.1	2.8e-13	7.4 ± 2.8	0.8 ± 0.7	-3.0	8.6e-8
4071	APC/C activator protein Cdc20p	DN4a–DN4e	19.3 ± 5.0	1.6 ± 0.3	-3.6	4.1e-19	17.3 ± 8.3	2.2 ± 0.3	-2.7	1.6e-11
5360	2-oxoglutarate dehydrogenase (mitoch.) Kgd1p	DN5a–DN5e	102.5 ± 18.3	9.4 ± 0.7	-3.5	1.8e-42	39.4 ± 5.0	6.0 ± 1.7	-2.5	1.3e-21
5958	Mitochondrial inner membrane protein Oxa1p	DN6a–DN6e	67.0 ± 7.5	3.9 ± 0.8	-4.1	5.5e-48	33.9 ± 6.7	1.3 ± 0.5	-4.4	5.94e-50

Promoters are named after four last digits of the locus tags of the genes they precede. Counts_norm_ are the average normalized counts from three biological replicates, normalized using the geTMM method [117]. The promoter from *MEP2* (RHTO_01680) in strain NP11 was also evaluated (strains UP10a-UP10e)

The selected candidate promoters were cloned in front of a *R. toruloides* codon-optimised green fluorescent protein (GFP) encoding gene [22]. The resulting reporter cassettes were integrated into BOT-A2 using random integration, since targeted integration is in general difficult to achieve in *R. toruloides* and has of yet not been successfully achieved in BOT-A2 (unpublished data). After transformation, five strains per randomly integrated candidate promoter construct were selected for assessment. A list of the assessed BOT-A2 promoters and the resulting strains can be found in Table 2; the strains with the NP11 *MEP2* promoter were named UP10a-e.

### Characterisation of promoter candidates upregulated upon nitrogen starvation

The strains containing the reporter cassettes were evaluated in two steps. Firstly, five strains per promoter construct were analysed with a BioLector microbioreactor for growth and GFP fluorescence. Secondly, one representative strain from each of the three most promising upregulated and three most promising downregulated promoter candidates was further characterized in shake-flask cultivations, which allowed for analysis of nitrogen and glucose consumption profiles. The assessment of the upregulated candidate promoters is described in this section, and the downregulated candidates in section "Characterisation of promoter candidates downregulated upon nitrogen starvation".

#### Assessment of upregulated promoter candidates using a microbioreactor

**Fig. 4:**
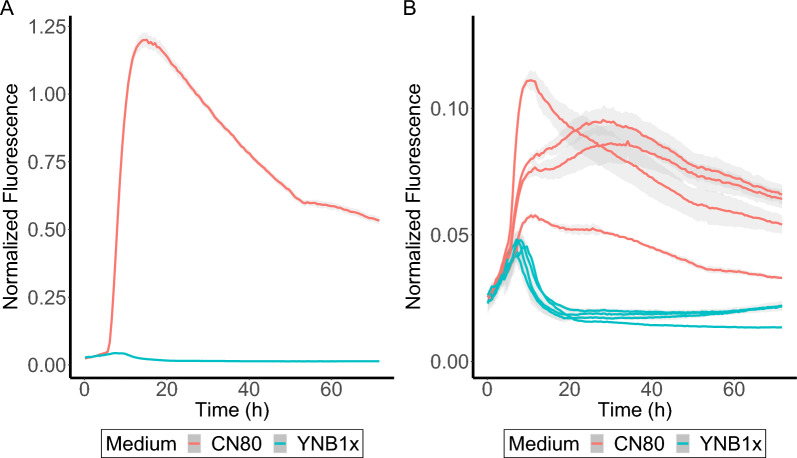
Fluorescence of strains transformed with the 3877p-GFP reporter cassette. Strains were grown in a BioLector in CN80 and YNB1x medium in triplicates in 96-well plates. Displayed are **A**: normalized fluorescence of strain UP1a over time, and **B**: normalized fluorescence of strain UP1b-e over time. Fluorescence was normalized to the scattered light values from the biomass (excitation at 620 nm). Results are an average of triplicates with shadows showing the standard deviation

Five strains for each of the ten upregulated promoter candidates (Table 2) were analysed for their GFP fluorescence response in two different conditions, each with ammonium sulphate as the nitrogen source: (1) YNB-CN80 with a g carbon:g nitrogen ratio of 80 (CN80), and (2) standard YNB1x medium with a CN ratio of 7.5. In most cases, no substantial differences between GFP expression in the control medium and in the induction medium could be observed (Additional file 1: Fig. S2). However, some strains did show an increased fluorescence in the induction medium compared to the control medium: UP1a (Fig. 4A), UP3e (Additional file 1: Fig. S3) and all strains of UP10 (Additional file 1: Fig. S4). UP1a specifically showed an interesting fluorescence profile (Fig. 4A) for upregulation during nitrogen starvation, with GFP expression starting approximately 6–8 h after the start of the CN80 cultivation. It was not possible to measure the nitrogen content in this setup, therefore, it was unclear from this experiment whether the induction was due to full or partial nitrogen depletion. Nitrogen depletion data from the RNAseq cultivation on the same CN80 medium however suggest that nitrogen is depleted at around 8–10 h, which could coincide with the start of GFP expression in UP1a. GFP expression started sooner in both UP3e and all UP10 strains, with fluorescence being observed immediately after the cultivation start, and increasing with cultivation time (Additional file 1: Fig. S3 and S4).

The analysis also revealed that the absolute GFP expression levels were not comparable between the different strains transformed with the same promoter construct (Fig. 4B, Additional file 1: Fig. S3), which can possibly be attributed to the random genomic integration as integration in different chromosomal regions has been shown to result in expression variability in *S. cerevisiae* [47]. For this reason, each strain was only compared with regard to its response to the two different media, and not with strains transformed with the same recombinant DNA. In this manner, a clear difference between the induction and control conditions was observed for some of the reporter cassettes (Fig. 4B). Strain UP1a was selected for further analysis since it showed the strongest GFP expression increase in the YNB-CN80 medium and the highest signal separation between the two different media among the five UP1 strains. Likewise, strain UP3e (with the pDB45 promoter cassette) and UP10a (with the pDB22 promoter cassette) were also selected for further characterization.

#### Characterisation of strains with the most promising upregulated promoters

**Fig. 5:**
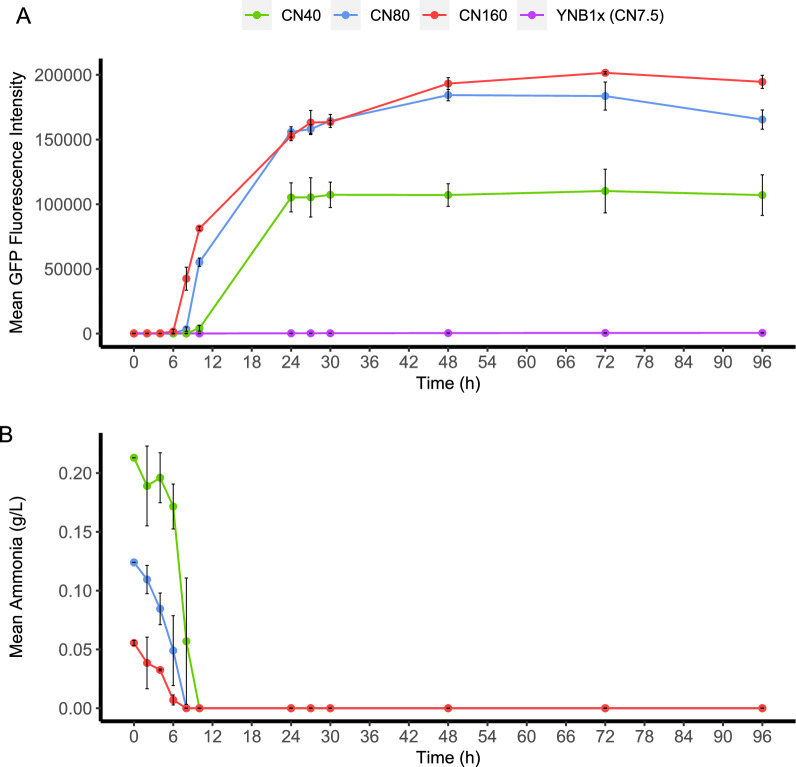
Analysis of strain UP1a. UP1a (promoter 3877p, from the gene RTBOTA2_003877 encoding a putative nitrate transporter) was cultivated in shake flasks in four different media (CN40, CN80, CN160, YNB1x). GFP fluorescence intensity (**A**) was analysed using flow cytometry; 100,000 events were captured. Ammonia concentration (**B**) was analysed using an enzymatic kit. OD_620nm_ and glucose consumption are available in Additional file 1: Figure S7, and ammonia concentration on the control medium YNB1x in Additional file 1: Figure S10. Error bars refer to standard deviation from two biological replicates

The three selected strains were further characterised during shake-flask cultivations to allow for sampling for additional measurements. Here, flow cytometry was used to evaluate the fluorescent signal on a population level. In addition to the YNB-CN80 and YNB1x media, the effect of the CN ratio on the induction profiles was also assessed by evaluating the strains in a YNB-CN40 medium, which had a slightly higher nitrogen content while still being considered nitrogen-limited. The most promising strain, UP1a, was also evaluated in YNB-CN160 medium to assess the effect of very low ammonium concentrations. All strains, including the BOT-A2 parental strain that was used as a control, depleted ammonium at the same rates, taking approximately 8 h to depletion in the CN160 and CN80 media, 10 h in CN40 medium, and 48 h in YNB1x control medium (Fig. 5; Additional file 1: Figs. S5, S6, S8, S10). The rates of glucose consumption and biomass formation varied based on the CN ratio of the medium but were otherwise comparable across all strains (Additional file 1: Figs. S5-S7).

**Fig. 6:**
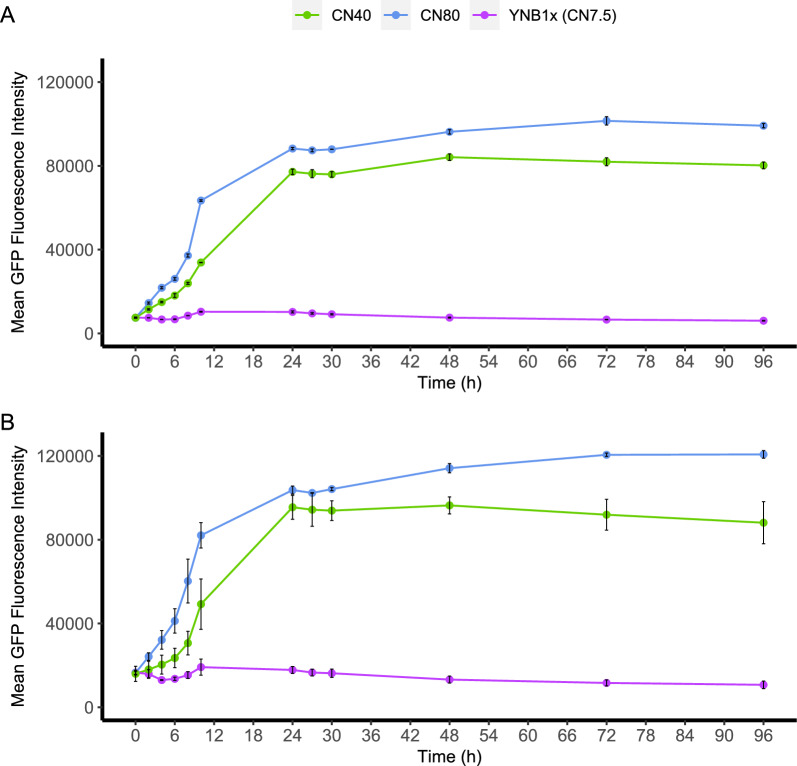
Fluorescence reporter analysis of strains UP3e and UP10a. UP3e (promoter 0480p, from the gene RTBOTA2_000480 putatively encoding the Carboxypeptidase Y inhibitor Tfs1p) (**A**) and UP10a (promoter *MEP2*p from NP11) (**B**) were cultivated in shake flasks in three different media (CN40, CN80, CN7.5). GFP was analysed using flow cytometry; 100,000 events were captured. Ammonia consumption profiles are available in Additional file 1: Figure S3 and glucose and OD_620nm_ in Additional file 1: Figure S3. Error bars refer to standard deviation from two biological replicates

The flow cytometry results were overall able to corroborate the BioLector results. GFP induction was found to be absent in all three strains when cultivated in the non-nitrogen limited YNB1x control medium (Figs. 5–6; Additional file 1: Figs. S11–S13). For strain UP1a, of the level of fluorescence that was observed during cultivation on YNB1x corresponded to the autofluorescence of the wild-type strain BOT-A2 (Additional file 1: Fig. S5). Fluorescence in all three strains was induced in the nitrogen-limited media and typically reached its peak in the sample taken at 24 h (Figs. 5–6; Additional file 1: Figs. S11–S13). There was a plateau in mean fluorescence signal following the peak at 24 h to the end of the cultivation (Figs. 5–6) that suggested that GFP levels remained constant during this time interval. This was corroborated by the GFP histograms (Additional file 1: Figs. S11–S13) which showed that the population remained at peak levels and did not revert back to the levels observed at the start of the experiment. Tendencies of subpopulation formation were observed in the GFP histograms from 24 h and onwards (Additional file 1: Figs. S11–S13). In all, this data suggests that the three assessed promoters were active in the induction media during the entire 96 h of cultivation.

Strain UP1a reached a substantially increased fluorescence in the shake flasks after only 6–10 h of cultivation, with the exact duration varying depending on the CN ratio of the medium (Fig. 5A). Depletion of nitrogen was faster in media with high CN ratios, which in turn resulted in earlier increases in the fluorescence signal. It was also observed that the absolute fluorescence levels in UP1a were substantially lower during CN40 cultivations than in the CN80 and CN160 cultivations. The ammonia measurements (Fig. 5B) indicated that ammonia depletion and upregulation of GFP (Fig. 5A) coincided, indicating that the promoter candidate (3877p) used in this reporter cassette responds very stringently to nitrogen availability, as GFP signal induction only was observed upon nitrogen depletion. Thus, we suggest that the promoter used in this construct is regulated by complete nitrogen-depletion and not just by nitrogen-limitation. Since no major difference could be seen between CN160 and CN80 in the UP1a cultivations, the other two strains, UP3a and UP10a, were only evaluated in the control medium YNB1x, and CN40 and CN80. Overall, strains UP3a and UP10a, behaved very similarly (Fig. 6). GFP expression started to increase immediately after inoculation, and thus is potentially connected to low nitrogen levels but not complete nitrogen depletion like UP1a. GFP expression was mainly repressed on the control medium, and highest on CN80.

**Fig. 7:**
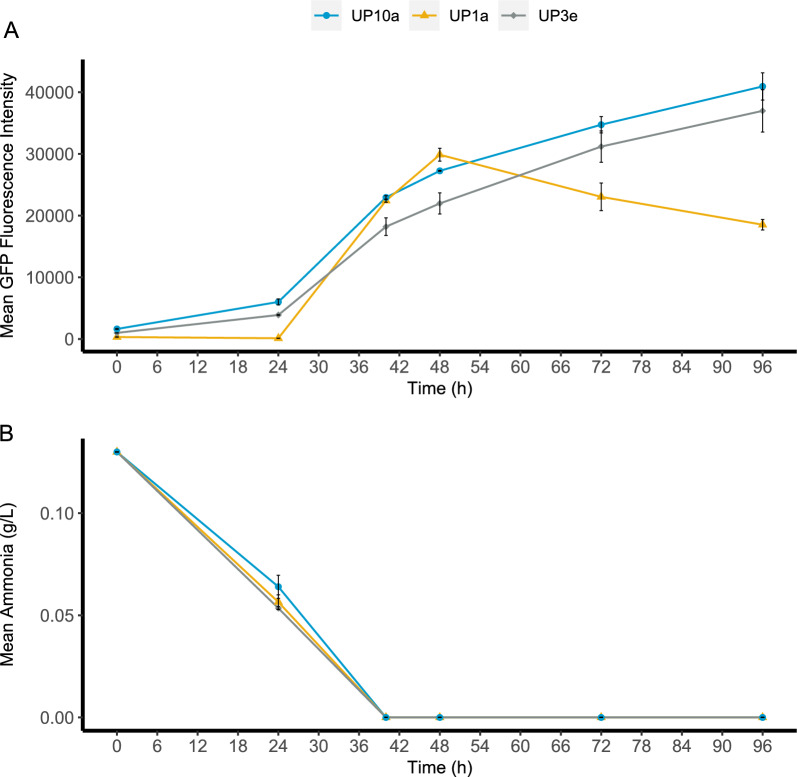
Analysis of strains UP1a (3877p), UP3e (0480p) and UP10a (*MEP2p*) in nitrogen-limited YNB media with 20 g/L xylose as carbon source (CN96). Displayed are (**A**) GFP fluorescence over time, and (**B**) the ammonia consumption profile. Strains were cultivated in shake flasks. GFP was analysed using flow cytometry, OD_620nm_, xylose consumption and xylitol formation profiles are available in Additional file 1: Figure SX5. Error bars refer to standard deviation from two biological replicates

The promoter candidates were initially picked not only for their high upregulation during nitrogen starvation on glucose, but also for upregulation during nitrogen starvation on xylose. Thus, shake-flask cultivations were also performed with xylose as a carbon source on CN80 for the three selected strains UP1a, UP3e and UP10a (Fig. 7). GFP expression was induced in a similar manner as in the respective glucose media, with expression starting at inoculation for UP3e and UP10a, but later for UP1a. However, xylose was consumed slower than glucose, which led to slower growth, and ultimately also slower nitrogen consumption. Nitrogen depletion therefore occurred at 38 h in CN80 medium when xylose was the carbon source, compared to depletion after 8 h for CN80 with glucose. In contrast to strains UP3e and UP10a, UP1a had a different GFP expression profile on xylose, with GFP levels decreasing after 48 h (Fig. 7A), which suggests that expression either decreased or completely ceased—in which case the observed decrease in fluorescence would correspond to the half-life of GFP.

### Characterisation of promoter candidates downregulated upon nitrogen starvation

BioLector microbioreactor cultivations were conducted for the six downregulated candidate promoter constructs in a similar way as for the upregulated ones, with the difference that with these reporter cassettes repression of GFP fluorescence was expected in the nitrogen-limited medium (CN80) and induction in the YNB1x medium. However, results from the experiment were inconclusive because of minimal differences between fluorescence of cells grown in induction or repression media. This was presumably due to differences in growth between the two media and the underlying mechanics of fluorescence and biomass measurements in the BioLector. Instead, a few strains from each of the six reporter constructs were grown in conical tubes for 24 h in the different media and evaluated by flow cytometry. In this manner, three strains, DN1a, DN2a and DN5c, that seemed to have a noticeably different fluorescence response in the two different conditions (data not shown) were identified and selected for further characterization in shake-flasks. OD_620nm_, glucose and ammonia measurements were comparable between all downregulated strains (Additional file 1: Figs. S14-S15), just like for the strains with the up-regulated reporter cassettes.

**Fig. 8:**
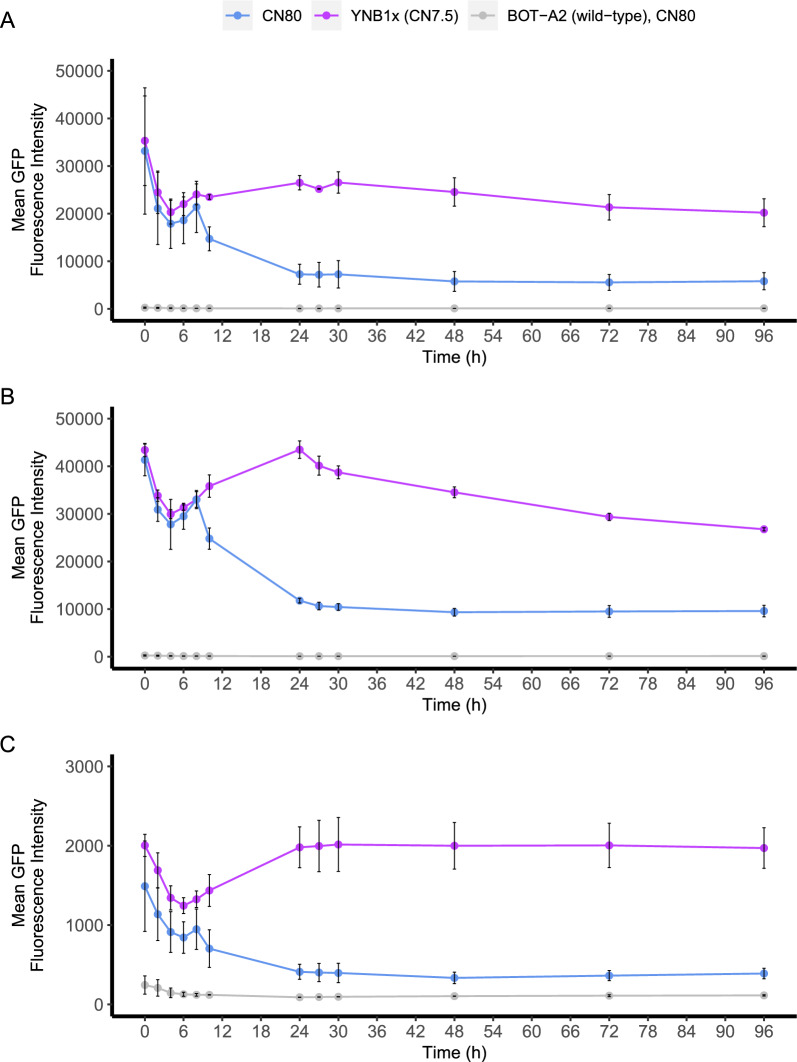
Fluorescence reporter analysis of strains (**A**) DN1a (0530p), (**B**) DN2a (3356p) and (**C**) DN5c (5360p). Strains were cultivated in shake flasks in two different media (CN80 and YNB1x). GFP was analysed using flow cytometry. The autofluorescence of the wild-type BOT-A2 strain was used as a control. Error bars refer to standard deviation from two biological replicates

Due to the persistence of the GFP protein in the cell, GFP reporter constructs are typically better at capturing upregulation conditions than downregulation conditions. This was also clear from our results (Fig. 8), as the GFP expression levels were high in the pre-cultures and showed high variability during the first 8 h of the experiment likely due to the different starting levels of GFP (Fig. 8). Attempts at improving the reproducibility of the GFP levels in the precultures were made by using YNB2x (13.4 g/L YNB; 20 g/L glucose) in the pre-cultures to limit growth, a phenomenon which we had observed for the upregulated strains where this medium was used for GFP repression by merit of its excess nitrogen levels. Despite this, the final biomass in the overnight cultures varied between OD_620nm_ 4–7 from culture to culture, and a direct corelation between pre-culture OD_620nm_ and the GFP levels at 0 h was observed. However, after 8–10 h of the shake flask cultivations, differences between the induction medium YNB1x and the control medium CN80 became clear for all three candidate strains (Fig. 8). It should be noted that the fluorescence levels of the repressing condition (CN80) never decreased all the way down to the levels of the autofluorescence of the wild-type strain BOT-A2 or the promoter-less control strain CTRL1a (Fig. 8; Additional file 1: Figs. S16–S20). This was especially pronounced for strains DN1a and DN2a that had overall higher GFP levels, which can likely be attributed to the genetic variation resulting from the random integration method used to generate these strains. While these results corroborate the RNAseq results that were used to select these promoters in the first place, they also imply that these promoters are not completely repressed under nitrogen-depletion, but continue to drive a low level of gene expression in the population.

## Discussion

### ANI analysis corroborated previous observations of major nucleotide differences between haploid *R. toruloides* strains of different mating types

Because of the history of taxonomical reclassification of strains from the *Rhodotorula* and *Rhodosporidium* genera [1], and the actual nucleotide level differences between sequenced *R. toruloides* strains (Fig. 1), there is still a lot to learn about DNA sequence compatibility between *Rhodotorula* strains. Sexual reproduction in *R. toruloides* is based on a bipolar mating type system in which haploid strains belong to either the MAT A1 or the MAT A2 mating type, and many of the known *R. toruloides* strains have been reported to have been isolated in haploid form [48]. It has previously been observed that genomes from haploid strains containing mating type loci from one of the mating types have high sequence identity to genomes from other strains of the same mating type, but can have a high level of nucleotide differences when compared to strains of the other mating type [44, 45]. The Average Nucleotide Identity (ANI) and pheromone receptor analysis of the currently available genome assemblies revealed that there are at least two different clusters within the *R. toruloides* species (Fig. 1), and that these two clusters correlate with the mating type loci present in the genome assemblies.

The MAT A1 cluster contained genome assemblies from the widely used NP11 strain, as well as 11 other *R. toruloides* strains, and the MAT A2 cluster contained genomes from seven strains, including BOT-A2. There were also three additional strains that did not cluster with any other strains at all, and one strain, CGMCC2.1609, that seemed to cluster in both the MAT A1 and the MAT A2 clusters. CGMCC2.1609 only had a MAT A1 homolog in our analysis though; however, the genome assembly size was 1.5 × that of the expected size (~ 33 Mb vs ~ 20 Mb), which might imply that this represents an aneuploid strain, or an incomplete assembly. The three strains that clustered outside the MAT A1 and MAT A2 clusters could belong to a different species of *Rhodotorula*, but further analysis of this was considered outside of the scope of this study. Strain CCT 0783 was reported to have been assembled as a diploid strain (genome size of ~ 40 Mb as compared to ~ 20 Mb) [49], and, interestingly, had homologous hits for both the A1 and the A2 pheromone receptors.

The ANI differences between MAT A1 and MAT A2 strains prompted us to analyse how pronounced the nucleotide differences were between strains for the promoter sequences identified in this study. Candidate sequences for the NP11 *MEP2*p and BOT-A2 3877p promoters were identified in all available genome assemblies and subjected to a multiple sequence alignment (MSA). Overall, the final MSAs revealed that there were several regions with high or identical nucleotide identity across all genomes. For the dissimilar regions, however, it was clear that the candidate promoter sequences from MAT A1 genomes clustered together and vice versa for the MAT A2 genomes (Additional file 4 & Additional file 5). The three strains that did not cluster in the MAT A1 and A2 clusters in the ANI analysis (Fig. 1) were also found to be outliers in terms of nucleotide sequence (Additional file 4& Additional file 5). Since the *MEP2*p promoter from NP11 (MAT A1) indeed functioned as expected when expressed in BOT-A2 (MAT A2) as shown in Fig. 6B, it is likely that key sequence motifs controlling the induction of the promoter are located within the regions that were conserved across all genomes.

Exactly how these genomic differences affect individual genes or might affect engineering endeavours within the species is not yet clear. Nevertheless, these results imply that an awareness of the nucleotide differences between *R. toruloides* strains will be useful for researchers working with this yeast species.

### RNA sequencing aided in generating a quality genome annotation for *R. toruloides* BOT-A2

In this study, a high-quality, functionally annotated genome assembly was generated for the oleaginous yeast *R. toruloides* BOT-A2, using long- and short read sequencing data. Of the 21 publicly available *R. toruloides* genomes in the NCBI database, only five have been annotated. With the BOT-A2 assembly, we present a sixth genome annotation for the species containing a gene model with 7001 predicted genes (mRNA, rRNA, and tRNA) and a 96.6% BUSCO completeness for core basidiomycete genes. The quality of the annotation can especially be attributed to the fact that RNAseq data of from the same strain was used as a guide for the gene prediction.

A few RNAseq studies have been conducted for *R. toruloides* previously, with various focuses: Phosphate limitation [5], stress tolerance in the presence of hydrolysate [11], effect of different carbon sources on growth [10, 39], and nitrogen limitation [14, 39]. The RNAseq data presented for nitrogen limitation in the current study adds an additional angle to the previous studies by being performed not only on glucose, but also on xylose—a pentose sugar of high industrial relevance due to its abundance in renewable feedstocks, i.e. plant waste hydrolysates. Overall, our transcriptomics results demonstrate similar trends as those that have been observed in the two previous nitrogen-limitation transcriptomics studies [14, 39]. Bomareddy et al. observed upregulation during nitrogen starvation of some TAG and acetyl-CoA synthesis related genes [39] that we also observed in our analysis, e.g. ATP-citrate lyase gene *ACL1* (RTBOTA2_005593), acetyl-CoA carboxylase gene *ACC1* (RTBOTA2_004388), the two fatty acid synthase subunit genes *FAS1* (RTBOTA2_004415) and *FAS2* (RTBOTA2_004570), and the diacylglycerol acyltransferase gene *DGA1* (RTBOTA2_000438). The aldehyde dehydrogenase gene *ALD2*, as well as the peroxisomal hydratase-dehydrogenase-epimerase gene *FOX2* were also highly upregulated in their study, whereas we observed a distinct downregulation during nitrogen starvation (*ALD2:* RTBOTA2_002577; *FOX2*: RTBOTA2_004652). The mitochondrial fumarate hydratase gene *FUM1* was reported to be downregulated during nitrogen starvation, but we observed a significant upregulation with a log_2_ fold change of 3.4 (RTBOTA2_002988). Zhu et al. likewise reported a significant upregulation for this gene in their analysis [14]. Discrepancies such as these are likely impacted by how the cells were cultured, when the samples were taken, the number of replicates used and media composition in the different studies, and the choice of data normalization and processing strategies.

Most of the differentially expressed genes in the central and lipid metabolism pathways reconstructed by Zhu et al. were found to be upregulated during nitrogen starvation [14], whereas we observed mainly downregulation, specifically for genes involved in the PPP and glycolysis, as well as the initial reactions of the carotenoid pathway. In addition to previous studies, our pathway reconstruction included putative genes from the phospholipid biosynthesis pathway. While further studies are needed to experimentally verify functions of the proposed genes and the carbon flux, the expression results suggest a general trend towards downregulation of the pathway during nitrogen starvation (Fig. 3).

### The genetic toolbox *R. toruloides* toolbox was expanded by identification of six promoters differentially active upon nitrogen depletion

The available promoter sequences for use in engineering of *R. toruloides* are steadily increasing, but the focus has typically been on identification of strong constitutive promoters [33–38]*.* Of the inducible promoters available for *R. toruloides* [37, 38], *DAO1p* and *ICL1p* were shown to be somewhat leaky during the tested repression conditions, and *CTR1p* had high variability between replicates unless a copper chelator was added [38]. The only promoter that has been reported to be regulated by nitrogen, *NAR1*, was not regulated by nitrogen depletion, but rather by the type of nitrogen source [38]. An ideal inducible promoter should be easy to induce by an altering environmental parameter, such as addition or depletion of a preferably cheap chemical compound or by changing a physiochemical parameter (temperature, pH, aeration, etc.); likewise, it should ideally be just as easy to repress, for instance to avoid expression of the gene of interest during certain process steps, such as pre-cultivations for biomass propagation; finally, an ideal inducible promoter should not be leaky, that is, not exhibit any expression at all—or as little as possible—under repressed conditions. The 3877 promoter performed very well in all of these regards due to its clear repression as long as nitrogen remained in the medium. This makes this promoter particularly interesting for metabolic engineering strategies desiring to control the expression of a particular gene or pathway to coincide with lipid accumulation.

After analysing 16 promoter candidates suggested by RNAseq data to be regulated by nitrogen availability, we were able to identify six promoters that were either induced or repressed during cultivation in nitrogen-limited media: three that were upregulated during nitrogen starvation and three that were downregulated during nitrogen starvation. The genes the upregulated promoters were derived from were annotated with functions that can all be connected to either nitrogen starvation directly, or stress conditions in general. Among the genes controlled by the upregulated promoters, the ammonium permease gene *MEP2* from NP11 and the nitrate transporter gene RTBOTA2_003877 have a clear relationship to nitrogen by putatively coding for transport proteins. The carboxypeptidase Y inhibitor gene *TFS1* (RTBOTA2_000480) has been shown to be related to DNA replication stress in general in *S. cerevisiae* [50]; it has, however, also been suggested as a signature gene for prediction of nitrogen deficiency [51]. When it comes to the downregulated promoters, their relationship to nitrogen is a little more complex. SSb2p (RTBOTA2_000530) is a ribosome-associated chaperone that has been suggested to control ribosome biogenesis in *S. cerevisiae* [52], and downregulation of ribosomal biogenesis genes has been observed during nitrogen starvation conditions in oleaginous yeasts [14, 53, 54]. It remains to be elucidated if this gene has the same function in *R. toruloides* as in *S. cerevisiae*, though. Diphosphomevalonate decarboxylase Mvd1p (RTBOTA2_003356) can indirectly be connected to nitrogen starvation as it catalyses one of the first steps in the carotenoid pathway, which in general was downregulated during nitrogen starvation in BOT-A2 (Fig. 3). This might suggest a redirection of the carbon flux from carotenoids to TAGs. It has also been shown in other naturally carotenoid producing yeasts that carotenoid production decreases when lipid production increases under stress conditions [55]. The final promoter was taken from the *KGD1* gene (RTBOTA2_005360), which presumably encodes an enzyme of the TCA cycle that catalyses the conversion of alpha-ketoglutarate to succinyl-CoA. Nitrogen starvation has been reported to negatively affect the activity of the Idh1p isocitrate dehydrogenase [56], which controls the reaction directly upstream of the reaction catalysed by Kgd1p (Fig. 3). This results in the accumulated isocitrate being converted back to citrate, transported out of the mitochondria via Ctp1p and converted into acetyl-CoA by Acl1p and thus made available for TAG synthesis [56]. Downregulation of *KGD1* (RTBOTA2_005360) thus fits with the current understanding of how nitrogen starvation affects the metabolism of oleaginous yeasts.

Due to the challenges of achieving targeted integration in *R. toruloides*, some of the previous studies on promoter characterization have, just like in the BOT-A2 case, relied on random chromosomal integration of the cassettes used for promoter evaluation [34, 38]. Other *R. toruloides* promoter studies have used KU70Δ strains to achieve targeted integration and have thus been able to analyse relative promoter strength by integrating single copies in the same integration locus for all their strains [33, 36, 37, 49]. Since targeted integrations, including deletions, have yet to be achieved in BOT-A2, we were not able to make any conclusions about promoter strength, but instead focused on the induction and repression dynamics of the promoters. Based on a recent observation that the electroporation protocol developed for BOT-A2 sometimes resulted in transformants with exponentially higher GFP expression levels and copy numbers of the reporter gene (unpublished data), we analysed five transformants for each GFP construct to assess the variability in fluorescence signal caused by the random integration. Indeed, a wide range of fluorescence intensity was observed between the different strains. In addition to copy number effects, we also hypothesise that the variability could be caused by differences in the integration sites. Random integration has been shown to result in integration in any region on the chromosomes in other yeasts with efficient NHEJ systems [57], and gene expression has been shown to vary across different chromosomal loci in the model yeast *S. cerevisiae* [47]. We noticed that the previous *R. toruloides* promoter studies that relied on random integration did not report on expression variability across strains transformed with the same constructs [34, 38]. These studies used *Agrobacterium tumefaciens* mediated transformation instead of electroporation, and it is possible that the high DNA amounts required for electroporation (~ 5 µg per GFP reporter) could lead to instances where multiple DNA fragments were taken up and integrated by the same cell and resulted in copy number effects in our strains.

The induction and repression dynamics in the nitrogen-limited and non-nitrogen limited media were in general comparable in each set of five strains when differences in signal strength were disregarded (Fig. 4; Additional file 1: Fig. S3, S4). There were, however, some examples where differences in expression patterns were observed for strains with the same construct (Additional file 1: Fig. S2). While the cause of these differences is yet to be understood, we speculate that the random integration can result in cases where the promoter is inactive. This could for instance be due to silencing of the integration locus (e.g. at centromeric or telomeric regions), truncation of the reporter cassette during integration, or integration into a native promoter region that resulted in a chimeric promoter with altered expression patterns.

Inclusion of the first intron from the 5′ untranslated region directly upstream of the start codon has been shown to improve expression levels when using *R. toruloides* promoters [36, 38], and therefore all the assessed promoter sequences in this study were selected to start directly in front of the start codon. Although the 16 promoter sequences analysed in this study were picked based on the same principles, not all promoter candidates seemed to be functional, as measured by their ability to drive GFP expression during the tested conditions. These promoter sequences were limited to approximately -1000 bases upstream of the stop codon for all constructs. A previous study on the *R. toruloides GPD1* promoter was able to show that using longer promoter sequences resulted in the inclusion of additional regulatory elements, which led to higher expression levels as assayed with GFP [58]. It is therefore possible that a longer sequence for the non-functional promoter sequences in the current study could result in GFP expression that better represents the RNAseq results from the corresponding genes.

## Conclusions

In the present study, we present six novel *R. toruloides* promoters regulated by nitrogen starvation or nitrogen depletion during growth on glucose, and also on xylose. Three of the promoters were upregulated by nitrogen depletion, and three promoters were downregulated. The promoters were identified using a high-quality genome sequence generated from Oxford Nanopore and Illumina reads, and a high-quality genome annotation of our natural *R. toruloides* isolate BOT-A2, as well as transcriptomics data for growth on nitrogen starvation for this yeast. While we want to stress that the random integration does not allow us to conclude anything about promoter strength, the results from the RNAseq, ammonium consumption, and GFP signal analyses corroborate that the identified promoters are upregulated or downregulated when cultivated in nitrogen-limited media. We showed that these novel promoters can potentially be used in different strains of the *R. toruloides* species and foresee that they will be a useful addition to the genetic toolbox of this emerging cell factory. Specifically 3877p was found to act like a switch, with GFP expression increasing just as ammonium sulphate is depleted, and could thus be used to induce expression of a desired gene during the nitrogen depleted phase. For instance, using this promoter it would be possible to selectively overexpress an enzyme from the fatty acid biosynthesis pathway only upon nitrogen depletion. Likewise, carotenoid production could be boosted during the nitrogen depletion phase by using the 3877 promoter, while promoters of specific genes of the fatty acid synthesis pathway are exchanged with the once repressed during the nitrogen depletion phase, thus allowing acetyl-CoA flux to redirect towards carotenoids. Of course, these promoters can also be used for production of non-native products.

## Materials and methods

### Strains and cultivation conditions

The natural yeast isolate *Rhodotorula toruloides* BOT-A2 [43] and its derived strains were used in all experiments and cultivated at 30 °C. A list of all strains can be found in Table 3. The strains were kept on YPD (20 g/L yeast extract (Sigma Aldrich), 10 g/L peptone from meat (Merck-Millipore), 20 g/L glucose) plates with 20 g/L agar. The BOT-A2 cryostock solution was stored in 25% (w/w) glycerol at -80 °C.

**Table 3:** *Rhodotorula toruloides* strains used in this study

Strain name	Simplified strain name	Relevant genotype	Reference
BOT-A2	–	Wild-type	[43]
TMB DB031–TMB DB035	UP10a–UP10e	BOT-A2 random integration of the reporter gene cassette from pDB22 (*MEP2p*-EGFP reporter gene, KanMX)	This study
TMB DB074–TMB DB078	CTRL1a–CTRL1e	BOT-A2 random integration of the reporter gene cassette from pDB23 (EGFP gene without promoter, KanMX)	This study
TMB DB079–TMB DB083	UP1a–UP1e	pDB43_2 (random integration)	This study
TMB DB084–TMB DB088	UP2a–UP2e	pDB44_3 (random integration)	This study
TMB DB089–TMB DB093	UP3a–UP3e	pDB45_2 (random integration)	This study
TMB DB094–TMB DB098	UP4a–UP4b	pDB46_2 (random integration)	This study
TMB DB099–TMB DB103	UP5a–UP5e	pDB47_2 (random integration)	This study
TMB DB104–TMB DB108	UP6a–UP6e	pDB48_2 (random integration)	This study
TMB DB109–TMB DB113	UP7a–UP7e	pDB49_2 (random integration)	This study
TMB DB114–TMB DB118	UP8a–UP8e	pDB50 (random integration)	This study
TMB DB119–TMB DB123	UP9a–UP9e	pDB51 (random integration)	This study
TMB DB124–TMB DB128	DN1a–DN1e	pDB52 (random integration)	This study
TMB DB129–TMB DB133	DN2a–DN2e	pDB53 (random integration)	This study
TMB DB159–TMB DB163	DN3a–DN3e	pDB55 (random integration)	This study
TMB DB164–TMB DB168	DN4a–DN4e	pDB56 (random integration)	This study
TMB DB169–TMB DB173	DN5a–DN5e	pDB57 (random integration)	This study
TMB DB174–TMB DB178	DN6a–DN6e	pDB58 (random integration)	This study

#### Cultivations for differential gene expression experiments

To study differential gene expression before and after nitrogen starvation, a defined medium, YNB-CN80, consisting of YNB (without ammonium sulphate and without amino acids) supplemented with glucose and ammonium sulphate to a carbon-to-nitrogen (CN) ratio of 80 (g/g) was used. The CN ratio of 80 was calculated as previously described for *Rhodotorula* yeasts [59] based on mass. The final medium was composed of 1.7 g/L YNB (without ammonium sulphate), 20 g/L glucose, a potassium buffer (2.299 g/L K_2_HPO_4_; 11.83 g/l KH_2_PO_4_) at pH 5.5, and 0.47 g/L ammonium sulphate for C/N 80.

BOT-A2 was pre-cultured in 50 mL of YPD in 250 mL shake flasks at 30 °C in a rotary shaker at 210 rpm after inoculation from single colonies grown on YPD plates. Pre-cultures were harvested by centrifugation, and the cell pellet was washed with sterile deionized water. Cells were then used to inoculate 120 mL of YNB-CN80 medium in 500 mL shake flasks to an optical density (OD_600nm_) of 0.1. Cultures were incubated at 30 °C and 210 rpm on a rotary shaker and sampled for differential gene expression at two consecutive time points with t_1_: exponential growth (non-depleted nitrogen levels) and t_2_: nitrogen starvation (determined by full nitrogen depletion). The experiment was conducted for two different carbon sources (glucose and xylose), leading to four conditions: glucose t_1_ and t_2_; xylose t_1_ and t_2_. Time points varied between the two sugars. The YNB-CN80 cultivations were performed in biological triplicates. Cultures were sampled for OD_600nm_ (Genesys 20, Thermo Fisher, Waltham, MA, USA), cell dry weight (CDW), ammonium levels, pH, total RNA and total lipid content. CDW was determined for all RNAseq time points in duplicates using a pre-weighed 0.45 μm polyethersulfone membrane filter (Sartorius AG, Göttingen, Germany). One mL of sample containing biomass was added to a filter, the filter was subsequently washed with water and then dried in a microwave oven for 15 min at 350 W. Immediately after drying, the filters were weighed using a micro scale (Precisa Gravimetrics AG, Dietikon, Switzerland). Levels of ammonium were measured using the Rapid Ammonia Assay Kit (Megazyme, Ireland). RNA, HPLC and lipid procedures are described in separate sections below.

#### Cultivations for BioLector fluorescence screening experiments

Pre-cultures for all tested strains were inoculated from single colonies and incubated overnight (approx. 16 h) in 5 mL YNB2x in 50 mL Falcon tubes and placed on a shaker at 200 rpm. YNB2x consists of 1.7 g/L YNB (without ammonium sulphate), 20 g/L glucose, a potassium buffer (2.299 g/L K_2_HPO_4_; 11.83 g/l KH_2_PO_4_) at pH 5.5, and 10 g/L ammonium sulphate. All BioLector cultivations were performed in biological triplicates. The medium used (either YNB-CN80 [see above] or YNB1x, a medium consisting of 1.7 g/L YNB (without ammonium sulphate), 20 g/L glucose, a potassium buffer (2.299 g/L K_2_HPO_4_; 11.83 g/l KH_2_PO_4_) at pH 5.5, and 5 g/L ammonium sulphate for C/N 7.5) was inoculated to an OD_600nm_ of 0.25 from the corresponding pre-culture and 200 μL of inoculated medium was added to each well of a 96-well polystyrene cell culture microplate (Greiner Bio-One International GmbH, Kremsmünster, Austria). The plate was sealed with a sterile AeraSeal film (Excel Scientific Inc., Victorville, CA, USA) and placed into the BioLector. Fluorescence and biomass were measured every 30 min for 72 h.

#### Cultivations for flow cytometry

The wildtype strain BOT-A2 and selected constructed strains were used for the flow cytometry experiments. Single colonies were used to inoculate 25 mL of YNB2x in 250 mL baffled shake flasks. For these experiments, optical density was measured at 620 nm using a Ultrospec 2100 Pro spectrophotometer (Amersham Biosciences, Uppsala, Sweden). Pre-cultures were cultivated overnight at 200 rpm and used to inoculate the main cultivations in either YNB-CN80 or YNB1x at an OD_620nm_ of 0.25. For the strain UP1a two additional media were used: YNB-CN40 and YNB-CN160, with the same composition as the previously described YNB-CN80 medium, but with 0.94 g/L and 0.24 g/L ammonium sulphate, respectively. Samples were taken regularly for flow cytometry, OD_620nm_, HPLC, and ammonia measurements.

### Extraction and sequencing of genomic DNA and total RNA

The Invitrogen Easy-DNA gDNA Purification Kit (Thermo Fisher) was used to extract high molecular weight genomic DNA (gDNA) from an overnight culture of *R. toruloides* BOT-A2 grown in YPD medium for long-read sequencing. Quantification of the extracted gDNA was done using a Qubit v3 fluorometer. The MinION sequencer (Oxford Nanopore Technologies) was selected for long-read sequencing, along with the Ligation Sequencing Kit (SQK-LSK110), using a R9.4.1 flow cell. 12.8 fmol of gDNA were used for sample preparation, which was in the required range for the Ligation Sequencing Kit. Additional short-read sequencing for polishing the long-read assembly was performed on the Illumina MiSeq (2 × 250 bp; ‘Version2' chemistry) platform at the SciLifeLab National Genomics Infrastructure, Sweden to complement the long reads generated from the MinION.

Total RNA was extracted from the different YNB-CN80 cultivations previously described; 12 RNAseq samples were processed in total (two different sugars at two different time points, three biological replicates per condition). At t_1_ a 7.5 mL sample was taken from the cultivations, and a 4-mL sample at t_2_. The samples were immediately centrifuged at 3,800xg and 4 °C for 5 min. After discarding the supernatant, the pellets were immediately quenched in liquid nitrogen and stored at -80 °C until extraction. Before mechanical lysis of the cells the pellet was resuspended in 500 μL TRIzol (Invitrogen, Waltham, MA, USA). Cells were then lysed using a Precellys evolution bead mill (Bertin Instruments, Montigny-le-Bretonneux, France) for 4 cycles à 45 s at 7200 rpm (OD_600nm_ 5–10), samples were kept on ice in between cycles. After lysis, another 500 μL of TRIzol was added to each sample, thoroughly vortexed, and incubated for 2–3 min at room temperature. The samples were then centrifuged for 10 min at 12,000 rpm at 4 °C (Centrifuge 5417R, Eppendorf AG, Hamburg, Germany). The supernatant was collected and transferred to a new RNAse-free tube. 200 μL of chloroform was added and the mixture was vortexed vigorously for 20 s, and then incubated for 2–3 min at room temperature. Samples were centrifuged again for 15 min at 12,000 rpm at 4 °C. After centrifugation, the top clear aqueous phase was removed carefully (approx. 350 μL) and transferred to a new RNAse-free tube. An equal amount of 100% absolute ethanol was added and mixed. The sample was loaded onto an RNeasy column, and from here on the RNeasy Mini Kit extraction protocol was followed (Qiagen, Germany; *Purification of Total RNA from Yeast* protocol), the suggested optional DNase clean-up step was included. RNAzap was used to clear the workspace from any RNases and DNases. The RNA quality was analysed using an Agilent 2100 Bioanalyzer. All samples had a RIN value of > 5. The RNA samples were sequenced at the SciLifeLab National Genomics Infrastructure, Sweden using the Illumina TruSeq Stranded mRNA library kit with Poly-A selection and sequencing on an Illumina NovaSeq6000 (2 × 150 bp).

### Fatty acid extraction and analysis

The KOH/ethanol extraction method [60] was used to extract fatty acids from the cultivations for RNA sequencing, with slight changes as previously described [43]. In short, after freeze-drying of the samples, biomass was determined using a micro scale. An internal standard of fatty acid 17:0 TAG at an initial concentration of 4 mg/mL in toluene was added to the dried biomass samples, volumes of internal standard added were adjusted to the amount of dried biomass. Subsequently, 2.5 mL of a 2.14 M KOH in 12% EtOH solution were mixed. Samples were incubated in a heat block for 2 h at 70 °C. Samples were acidified to a pH of 2 by addition of 1.25 mL of 5 M HCl. Then, fatty acids were extracted by adding hexane in 3 steps with 2 + 1.5 + 1.5 mL. The upper phases were collected, and pooled extracts were evaporated under a flow of nitrogen gas at 40 °C. Fatty acids were methylated by addition of 1 mL of 10% acetyl chloride in methanol and 1 mL of toluene. The samples were incubated again in a heat block for 2 h at 70 °C, and then resuspended in 0.4 ml milliQ and 2 ml petroleum ether/diethyl ether (80:20) and vigorously mixed. The upper phase was collected and then evaporated under a flow of nitrogen gas at 40 °C. Final extracts were then resuspended in 1 mL isooctane before analysis and storage.

Analysis of the fatty acid methyl esters (FAME) was performed with a GC–MS (Agilent technologies, USA: GC 7890A, MSD 5975C) with a DB-WAX column (0.25 × 30 mm, 0.25 µm film thickness) at a constant flow of 1 mL/min and helium as carrier gas. The internal C17:0 standard was used for calculation of the fatty acid content.

### Analysis of glucose, xylose and xylitol using HPLC

Analysis of the samples from the RNA sequencing experiment was performed with a JASCO UV/RI HPLC system (JASCO, Easton, MD, USA). Both, a Rezex ROA-Organic Acid H + (8%) column (Phenomenex, Torrance, CA, USA) and a guard column (Phenomenex, Torrance, CA, USA) were used with 5 mM H_2_SO_4_ as eluent with a flow rate of 0.8 mL/min and a 5% methanol wash buffer. Five μL of sample was injected and run for 18 min at 80 °C and 46 bar. Peaks were detected with a refractive index (RI) detector. The HPLC was equilibrated for approximately one hour before each run. Peaks were analysed with the ChromNAV program. Samples were prepared before injection onto the column by filtering through 0.2 μm nylon filters (VWR International, Radnor, PY, USA) and followed by a 2 × dilution with Milli-Q water.

Analysis of the samples from the flow cytometry experiments was performed with a Waters HPLC system (Milford, MA, USA) with an Aminex HPX-87H ion exchange column (Bio-Rad, Hercules, CA, USA). The column temperature was 60 °C, 5 mM H_2_SO_4_ flowing at 0.6 mL/min was used as mobile phase and a refractive index detector (Waters model 2414) was used for detection. Chromatograms were analysed with the Empower 3 software (Waters, Milford, MA, USA).

### Genome assembly

The genome and transcriptome data were analysed using a bioinformatics workflow that we previously developed for another basidiomycete yeast [54]. The details of how the workflow was applied to BOT-A2 are presented in Sects. 5.5–5.8. A de novo assembly was generated using the MinION reads from strain BOT-A2. Guppy (v4.2.2 + effbaf8; Oxford Nanopore Technologies) was used to basecall the reads with the dna_r9.4.1_450bps_hac.cfg config, and FastQC (v0.11.9; [61]) was used to analyse read quality. Trimming and adapter removal was performed using Porechop (v0.2.4; [62]) with *–discard_middle* settings, and Nanofilt (v2.7.1; [63]). To find the most suitable assembler for the long-read BOT-A2 data four different assemblers were tested: miniasm (v0.3_r179; [64]) with the minimap2 mapper (v2.11; [65]); Canu (v1.5; [66]); Flye (v2.8.2; [67]) and Shasta (v0.6.0; [68]). Assembly quality was evaluated with Quast (v5.0.2; [69]). The miniasm assembly was found to give the best assembly results and was chosen for further polishing. Racon (v1.4.13; [70]) followed by Medaka (v0.5.2; Oxford Nanopore Technologies) was used for error-correction using the base-called MinION reads. Nanopolish (v0.13.2; [71]) was then used with the non-basecalled raw signals (fast5) from the sequencer for another round of polishing; Nanopolish was run together with bwa (v0.7.17; [72]) and samtools (v1.10; [73]). The outcome of each error-correction step was assessed with MUMmer-dnadiff (v4.0.0rc1; [74]). Finally, Illumina short-read data from BOT-A2 was used to polish the assembly, using POLCA from the MaSuRCA package (v.3.4.2; [75, 76]).

### RNAseq data processing and transcriptome assembly

The 12 RNAseq samples from the 4 different conditions in triplicates were pre-processed in preparation for downstream analysis (assembly of the transcriptome as well as differential gene expression analysis). Read quality was assessed using FastQC (v0.11.9; [61]) and MultiQC (v1.10.1; [77]) was used to get an overview of the sequencing quality of all 12 samples. Two samples were over-sequenced during the initial sequencing run (400 M and 69 M reads respectively) and had overtaken the flow cell, diminishing the reads produced by the other samples. To correct for this, the remaining 10 were sequenced again, and the over-sequenced samples were downsampled to 40 M reads (the range of the highest of the other 10 samples) with seqtk (v1.2-r101; [78]); all following analyses were done on the 10 resequenced and 2 downsampled samples. The reads were quality trimmed with TrimGalore (v0.6.1; [79]) and adapter removal was performed running cutadapt (v2.3; [80]). The trimmed reads were mapped to the final BOT-A2 genome assembly with Hisat2 (v2.2.1; [81]) using the *–dta* option. The resulting alignments were sorted and indexed with samtools (v1.10; [73]), and mapping statistics were assessed with RSeQC (v2.6.4; [82]) for quality control.

StringTie (v2.1.4; [83]) was used to assemble the transcriptomes from all 12 samples, with the Hisat2 alignments as indata. All 12 transcriptome assemblies were then used to generate a non-redundant transcriptome using the StringTie *–merge* option. The final transcriptome was utilised as transcript evidence for the genome annotation pipeline.

### Genome annotation

The MAKER pipeline (v3.01.2-beta; [84]) was used to build gene models for the final BOT-A2 assembly. MAKER was then run iteratively in three rounds. The following indata was used to build the initial gene model: (1) the final BOT-A2 assembly; (2) the StringTie-assembled transcripts from BOT-A2 (11425 transcripts including isoforms) (3) protein sequences from the IFO0880_v4 gene model (8490 sequences; [85]) and proteome data from the to BOT-A2 genetically similar strain ATCC 204091 (2816 sequences, UniProt: UP000006141; [86]), and (4) filtered repeat sequences identified in the BOT-A2 assembly (described below; used to soft-mask the genome during the MAKER runs). Repeat sequences were identified and masked using RepeatModeler (2.0.1; [87]) calling on RepeatMasker (v4.1.1) and RMBlast (v2.9.0-p2). The RepeatModeler option “-engine ncbi”, the RepBaseRepeatMaskerEdition-20181026 repetitive DNA elements database [88]), and the Dfam database [89] included with RepeatMasker (v4.1.1). Transposon detection was performed with TransposonPSI (v1.0.0; [90]) and the putative sequences were removed from the assembly using fasta_removeSeqFromIDlist.pl (GAAS v1.2.0; https://github.com/NBISweden/GAAS), blastx (v2.2.29 + ; [91]) and ProtExcluder (v1.2; [92]). The identified repeats from the BOT-A2 assembly were also to remove putative transposons sequences from the IFO0880_v4 and ATCC 204091 proteomes before running MAKER.

Three different ab initio gene predictors were used in the MAKER pipeline: SNAP (v2013_11_29; [93]), GeneMark (v4.62 [94]) and Augustus (v3.2.3; [95]). Braker2 (v2.1.5–20210115-e98b812; [96, 97]) was used to train the Augustus model. The Braker2 pipeline was aided by a GeneMark (v4.62 [94]) model built with Hisat2 mapped RNAseq data and the option—fungus; the Braker2 filtering step was done using Diamond (v0.9.31; [98]). The MAKER pipeline was iterated using the following inputs: Round one was run with only the indata listed in the previous paragraph. In round two, three additional gene models were trained and used as auxiliary input to MAKER: SNAP was trained on the gene model produced by round one of MAKER; Augustus and GeneMark were trained on the Hisat2 mapped RNAseq data. In the third round, a new Hisat2 mapping was performed by aligning the RNAseq reads the gene model produced by round two of MAKER, and the new mapping was used for training of new Augustus and GeneMark models. The quality of the gene models produced by the three rounds of the MAKER pipeline were assessed by three different metrics: (1) the number and average length the predicted genes; (2) the AED (Annotation Edit Distance) annotation quality scores [99]; and 3) the BUSCO completeness in terms of essential basidiomycota genes (v5.0.0; basidiomycota_odb10 (v2020-09-10) database; [100]). Eventually, the gene model produced by the second round of MAKER was selected as the best performing model, and used in the subsequent analyses.

The protein sequences of the final gene model were translated from the predicted genes and used for functional annotation of the genome assembly. The proteins were annotated by homology to proteins in the Uniprot database [101] using local blastp databases (v2.11.0 + ; [91]). InterproScan (v5.30–69.0; [102]) was used to assign Gene Ontology, Pfam, Superfamily and Interpro annotations to each protein. As previously described [54], the putative gene annotations were harmonized to those of other yeasts by prioritizing the use of names from the model yeast *S. cerevisiae*. The local blast databases were built using *S. cerevisiae* proteins (Uniprot proteome: UP000002311), basidiomycota proteins (Uniprot KB query “taxonomy:basidiomycota”) and *Rhodosporidium* proteins (Uniprot proteome: UP000006141). The blast results were filtered to only include hits with a blast e-value ≥ 1e-06 and blast score of > 100, and the best hit for each queried putative BOT-A2 protein was applied to the annotation file using AGAT (v0.6.0; [103]). Orthofinder (v 2.5.2; [104]) was used to validate the results of the blastp analysis against the proteomes of *S. cerevisiae* (Uniprot proteome: UP000002311), *Y. lipolytica* (Uniprot proteome: UP000001300), and *R. toruloides* NBRC0880 (Uniprot proteome: UP000239560).

### Differential gene expression

The differential gene expression levels of the predicted BOT-A2 genes were determined in all four different conditions: nitrogen depletion on glucose compared to exponential growth on glucose (g2g1); nitrogen depletion on xylose compared to exponential growth on xylose (x2x1); exponential growth on xylose compared to exponential growth on glucose (x1g1); nitrogen depletion on xylose compared to nitrogen depletion on glucose (x2g2). The read counts per gene were quantified using subread-featureCounts (v2.0.0; [105]) and the previously generated Hisat2 read mappings. DESeq2 (v1.26.0; [106]) was used to calculate differential gene expression using Rstudio v1.1.456 and R (v3.6.1, 2019–07-05 [107]). A threshold was set for genes to have a minimum of five reads in at least three of the 12 analysed samples, and genes were filtered out and removed from the subsequent analysis if they did not fulfil those requirements. Expression counts were normalised using the built-in median of ratios normalisation function in DESeq2 before the actual differential expression analysis. Expression count data was normalised with VST (variance-stabilizing-transformation) for PCA and cluster analysis. Gene dispersion was analysed with DESeq2::estimateDispersions and tested with Wald’s test (nbinomWaldTest). EnhancedVolcano (v1.4.0; [108]) was used to generate volcano plots of the differential expression data and the different analysed conditions.

### Average nucleotide identity and pheromone receptor analysis

Average Nucleotide Identity (ANI) analysis [109] was used to assess the nucleotide similarity of all the 22 currently publicly available *R. toruloides* genomes. The analysis was done with pyani (v0.2.12; [110]) using MUMmer (v4.0.0rc1; [74]) for the alignment. All genome assemblies, except the BOT-A2 assembly generated in this study, were downloaded from the NCBI Genome Database. The accession numbers for the specific versions of the downloaded assemblies were: GCA_001542265.1; GCA_001542305.1; GCA_000222205.2; GCA_921037615.1; GCA_016808315.1; GCA_003234015.1; GCA_001456015.1; GCA_023968905.1; GCA_000988805.1; GCA_001255795.1; GCA_001600115.1; GCA_001600135.1; GCA_001600155.1; GCA_001600215.1; GCA_000258745.1; GCA_007990605.1; GCA_000320785.2; GCA_000988875.2; GCA_005387725.1; GCA_023078535.1; GCA_024734855.1.

For the pheromone receptor analysis, the MAT A1 protein (M7X934 from strain NP11) and the MAT A2 protein (G0T0M8 from strain ATCC 204091) were used. The proteins were used as input for a tblastn analysis and were compared against all on NCBI available *R. toruloides* genome sequences. The sequence identity for either MAT A1 or MAT A2 was never 100% (not even in NP11 or ATCC 204091) since tblastn converts amino acids to the corresponding DNA coding sequence, which results in an intron-less sequence. Therefore, the matches cannot be perfect since the genome sequences used do contain introns. During the analysis of the CBS14 genome assembly, it was found that there were two contigs named “scaffold 27” that had identical blast results for the query proteins. These were thus suspected to be duplicates, and one of them was disregarded during the analysis: only the results from contig CAKLCE020000050.1 were used.

### Promoter identification and multiple sequence alignment analysis

Fifteen significantly differentially expressed genes with an annotation other than *hypothetical protein* fulfilling the additional |log_2_ fold change|≥ 2 threshold for both g2g1 and x2x1 were selected for promoter characterisation. The putative promoter sequences were taken directly upstream of the start codon of each selected gene in the BOT-A2 assembly. The final promoter sequence lengths were all in the range of 600 bp to 1000 bp. A candidate *MEP2* promoter sequence was extracted from the NP11 genome (GenBank: GCF_000320785.1; [14]) by taking 1000 bp directly upstream of the *MEP2* gene (locus tag RHTO_01680). The sequences of all candidate promoters identified in this study are available in Additional file 3.

The *MEP2* candidate promoter sequence from NP11 and the 3877 candidate promoter sequence from BOT-A2 were used as input for a nucleotide Blast analysis [111] to identify the corresponding sequences in the other *R. toruloides* genomes. Identified sequences were then aligned with muscle [112] using the multiple sequence alignment *msa* package (v1.30.1; [113]) in R.

### Plasmid construction

A plasmid named pDB22 containing the *MEP2* promoter from NP11 as part of a GFP expression cassette and a KanMX selection marker was designed in silico and synthesised (GenScript, Netherlands). The fluorescent reporter cassette contained a *R. toruloides* codon-optimized GFP (GenBank: JQ806388.1; [22]), and a *Agrobacterium tumefaciens* nopaline synthase (NOS) terminator (using the sequence from the map of the *R. toruloides* plasmid NM1-5S-tRNA-SgH; Addgene plasmid # 128178; [26]). A *R. toruloides* KanMX antibiotic selection marker cassette [38] was placed directly downstream of the reporter cassette. The nucleotide sequence for the KanMX cassette was obtained from the NM9-SpCas9-NLS3 plasmid map (Addgene plasmid # 128177; [26]). A cloning plasmid was constructed by taking pDB22 and exchanging the *MEP2* promoter sequence for a Eco72I blunt restriction site. Primers RtEGFP_1F_phos and RtCAR2_750R_Eco72I_phos, containing tails with the Eco72I site and 5’-phosphorylations, were used to PCR amplify the backbone of pDB22 without the promoter. Phusion polymerase with the GC buffer (Thermo Fisher) was used for the amplification. The PCR product was treated with DpnI to digest the original template plasmid, and the resulting DNA was self-ligated using the T4 ligase (Thermo Fisher) to form a circular plasmid. The verified plasmid was named pDB23, and was used to construct the reporter cassettes for the BOT-A2 promoter sequences.

*R. toruloides* BOT-A2 genomic DNA was extracted from using the Yeast DNA Extraction Kit (Thermo Fisher) and used as template for cloning the promotor sequences. Primers for cloning the sequences were ordered with 5’-phosphorylations, and PCR amplification was performed using the Phusion polymerase and its GC buffer (Thermo Fisher). The PCR products were cleaned using the GeneJet PCR Purification kit (Thermo Fisher). The cloning plasmid pDB23 was digested using Eco72I and each PCR product was ligated using blunt ligation using the T4 ligase (Thermo Fisher).

Plasmids were subcloned in *Escherichia coli* NEB5α (New England Biolabs, MA, US) using the Inoue transformation protocol [114]. *E. coli* were grown on lysogeny broth medium (LB; 10 g/L tryptone, 5 g/L yeast extract, 10 g/L NaCl) supplemented with 100 µg/ml ampicillin with required. *E. coli* colony PCR [115] was used to identify correct ligations, using Dreamtaq polymerase with the recommended default settings from the manufacturer (Thermo Fisher). Agarose electrophoresis [116] was used to analyse the PCR products. Plasmids were extracted from overnight cultivations of the verified colonies using the GeneJet plasmid MiniPrep kits (Thermo Fisher). All plasmids were verified with Sanger sequencing (Eurofins Genomics, Germany). All plasmids used in this study can be found in Additional file 1: Table S3, and all primer sequences in Additional file 1: Table S4.

### Yeast transformation

Wild-type BOT-A2 was transformed with electroporation using a recently developed in-house protocol  (unpublished data). DNA fragments containing reporter gene and selection marker cassettes were inserted in the genome using the endogenous non-homologous end-joining system. The linear DNA fragments containing the reporter cassettes with the different promoters and the selection marker were amplified from each corresponding plasmid with the primers RtCAR2_731F and RtCAR2_730R, and the PCR products were purified as described above. The DNA fragments were added to the transformation mix at an amount of approximately 1 µg/kb of DNA fragment. Transformants were selected for on YPD plates with geneticin (G418, 200 µg/mL; Sigma-Aldrich, MO, US) after incubation at 30 °C for 3–4 days. Presence of the DNA fragment in the transformant genomes was verified by colony PCR [115].

### Flow cytometry

The fluorescence intensity of the GFP-containing cells was analysed on a single-cell level with a BD Accuri C6 Plus flow cytometer (Becton–Dickinson, NJ, US). The fluidics were set to a flow rate of 14 μL/min and a core size of 10 μm. 100,000 events were collected for each sample. Cells were excited by laser with a wavelength of 488 nm and a 533/30 nm bandpass filter was used to detect the GFP signal. A threshold of ≥ 80,000 on the forward scatter-height (FSC-H) channel was used to distinguish events from noise. Data analysis was performed with FlowJo (v10.8.1; Treestar Inc., CA, US). The geometrical mean of the GFP channel (533/30 nm) was used to calculate the average GFP signal intensity of the population of each sample.

### Supplementary Information

**Additional file 1:** Supplementary figures and tables.

**Additional file 2.** The master table Excel sheet with all annotations and differential expression results for each predicted ORF.

**Additional file 3.** A multi-FASTA file containing the promoter sequences used in this study.

**Additional file 4.** Multiple sequence alignment results are for the *MEP2* promoter.

**Additional file 5.** Multiple sequence alignment results are for the 3877 promoter.

## Data Availability

The final annotated genome was deposited at DDBJ/ENA/GenBank under the accession JAOEGQ000000000. The version described in this paper is version JAOEGQ010000000. The raw genome reads generated by MinION and Illumina were uploaded to the Sequence Read Archive (SRA) with accession numbers SRR17138794 and SRR17138793. The RNAseq datasets have been deposited in NCBI's Gene Expression Omnibus and are accessible through GEO Series accession number GSE217097. The transcriptome assembly has been deposited at DDBJ/EMBL/GenBank in Bioproject PRJNA882640. The BOT-A2 strain is available upon request from Chalmers University of Technology, Sweden, and is currently being deposited at the German Collection of Microorganisms and Cell Cultures (DSMZ).

## References

[1] Wang QM, Yurkov AM, Göker M, Lumbsch HT, Leavitt SD, Groenewald M, Theelen B, Liu XZ, Boekhout T, Bai FY (2015). Phylogenetic classification of yeasts and related taxa within Pucciniomycotina. Stud Mycol.

[2] Park Y-K, Nicaud J-M, Ledesma-Amaro R (2018). The engineering potential of *Rhodosporidium toruloides* as a workhorse for biotechnological applications. Trends Biotechnol.

[3] Wen Z, Zhang S, Odoh CK, Jin M, Zhao ZK. *Rhodosporidium toruloides*—a potential red yeast chassis for lipids and beyond. FEMS Yeast Res. 2020. 10.1093/femsyr/foaa038. 10.1093/femsyr/foaa038 PMC7334043 32614407

[4] Abeln F, Chuck CJ (2021). The history, state of the art and future prospects for oleaginous yeast research. Microb Cell Fact.

[5] Wang Y, Zhang S, Zhu Z, Shen H, Lin X, Jin X, Jiao X, Zhao ZK (2018). Systems analysis of phosphate-limitation-induced lipid accumulation by the oleaginous yeast Rhodosporidium toruloides. Biotechnol Biofuels.

[6] Wiebe MG, Koivuranta K, Penttilä M, Ruohonen L (2012). Lipid production in batch and fed-batch cultures of *Rhodosporidium toruloides* from 5 and 6 carbon carbohydrates. BMC Biotechnol.

[7] Wu S, Zhao X, Shen H, Wang Q, Zhao ZK (2011). Microbial lipid production by *Rhodosporidium toruloides* under sulfate-limited conditions. Biores Technol.

[8] Yaegashi J, Kirby J, Ito M, Sun J, Dutta T, Mirsiaghi M, Sundstrom ER, Rodriguez A, Baidoo E, Tanjore D (2017). *Rhodosporidium toruloides*: a new platform organism for conversion of lignocellulose into terpene biofuels and bioproducts. Biotechnol Biofuels.

[9] Lee JJL, Chen L, Shi J, Trzcinski A, Chen W-N (2014). Metabolomic profiling of *Rhodosporidium toruloides* grown on glycerol for carotenoid production during different growth phases. J Agric Food Chem.

[10] Jagtap SS, Deewan A, Liu J-J, Walukiewicz HE, Yun EJ, Jin Y-S, Rao CV (2021). Integrating transcriptomic and metabolomic analysis of the oleaginous yeast *Rhodosporidium toruloides* IFO0880 during growth under different carbon sources. Appl Microbiol Biotechnol.

[11] Qi F, Zhao X, Kitahara Y, Li T, Ou X, Du W, Liu D, Huang J (2017). Integrative transcriptomic and proteomic analysis of the mutant lignocellulosic hydrolyzate-tolerant *Rhodosporidium toruloides*. Eng Life Sci.

[12] Hu C, Zhao X, Zhao J, Wu S, Zhao ZK (2009). Effects of biomass hydrolysis by-products on oleaginous yeast *Rhodosporidium toruloides*. Biores Technol.

[13] Zhao X, Peng F, Du W, Liu C, Liu D (2012). Effects of some inhibitors on the growth and lipid accumulation of oleaginous yeast *Rhodosporidium toruloides* and preparation of biodiesel by enzymatic transesterification of the lipid. Bioprocess Biosyst Eng.

[14] Zhu Z, Zhang S, Liu H, Shen H, Lin X, Yang F, Zhou YJ, Jin G, Ye M, Zou H (2012). A multi-omic map of the lipid-producing yeast *Rhodosporidium toruloides*. Nat Commun.

[15] Zhang S, Ito M, Skerker JM, Arkin AP, Rao CV (2016). Metabolic engineering of the oleaginous yeast *Rhodosporidium toruloides* IFO0880 for lipid overproduction during high-density fermentation. Appl Microbiol Biotechnol.

[16] Fillet S, Gibert J, Suárez B, Lara A, Ronchel C, Adrio JL (2015). Fatty alcohols production by oleaginous yeast. J Ind Microbiol Biotechnol.

[17] Fillet S, Ronchel C, Callejo C, Fajardo M-J, Moralejo H, Adrio JL (2017). Engineering *Rhodosporidium toruloides* for the production of very long-chain monounsaturated fatty acid-rich oils. Appl Microbiol Biotechnol.

[18] Cao M, Tran VG, Qin J, Olson A, Mishra S, Schultz John C, Huang C, Xie D, Zhao H (2022). Metabolic engineering of oleaginous yeast *Rhodotorula toruloides* for overproduction of triacetic acid lactone. Biotechnol Bioeng.

[19] Geiselman GM, Zhuang X, Kirby J, Tran-Gyamfi MB, Prahl J-P, Sundstrom ER, Gao Y, Munoz NM, Nicora CD, Clay DM (2020). Production of ent-kaurene from lignocellulosic hydrolysate in *Rhodosporidium toruloides*. Microb Cell Fact.

[20] Zhuang X, Kilian O, Monroe E, Ito M, Tran-Gymfi MB, Liu F, Davis RW, Mirsiaghi M, Sundstrom E, Pray T (2019). Monoterpene production by the carotenogenic yeast *Rhodosporidium toruloides*. Microb Cell Fact.

[21] Wehrs M, Gladden JM, Liu Y, Platz L, Prahl J-P, Moon J, Papa G, Sundstrom E, Geiselman GM, Tanjore D (2019). Sustainable bioproduction of the blue pigment indigoidine: expanding the range of heterologous products in *R. toruloides* to include non-ribosomal peptides. Green Chem.

[22] Liu Y, Koh CMJ, Sun L, Hlaing MM, Du M, Peng N, Ji L (2013). Characterization of glyceraldehyde-3-phosphate dehydrogenase gene RtGPD1 and development of genetic transformation method by dominant selection in oleaginous yeast *Rhodosporidium toruloides*. Appl Microbiol Biotechnol.

[23] Takahashi S, Okada H, Abe K, Kera Y (2014). Genetic transformation of the yeast Rhodotorula gracilis ATCC 26217 by electroporation. Appl Biochem Microbiol.

[24] Tsai Y-Y, Ohashi T, Kanazawa T, Polburee P, Misaki R, Limtong S, Fujiyama K (2017). Development of a sufficient and effective procedure for transformation of an oleaginous yeast, *Rhodosporidium toruloides* DMKU3-TK16. Curr Genet.

[25] Liu H, Jiao X, Wang Y, Yang X, Sun W, Wang J, Zhang S, Zhao ZK. Fast and efficient genetic transformation of oleaginous yeast *Rhodosporidium toruloides* by using electroporation. FEMS Yeast Res. 2017. 10.1093/femsyr/fox017. 10.1093/femsyr/fox017 28369336

[26] Schultz JC, Cao M, Zhao H (2019). Development of a CRISPR/Cas9 system for high efficiency multiplexed gene deletion in *Rhodosporidium toruloides*. Biotechnol Bioeng.

[27] Jiao X, Zhang Y, Liu X, Zhang Q, Zhang S, Zhao ZK (2019). Developing a CRISPR/Cas9 system for genome editing in the basidiomycetous yeast *Rhodosporidium toruloides*. Biotechnol J.

[28] Otoupal PB, Ito M, Arkin AP, Magnuson JK, Gladden JM, Skerker JM (2019). Multiplexed CRISPR-Cas9-based genome editing of *Rhodosporidium toruloides*. mSphere.

[29] Castañeda MT, Nuñez S, Garelli F, Voget C, De Battista H (2018). Comprehensive analysis of a metabolic model for lipid production in *Rhodosporidium toruloides*. J Biotechnol.

[30] Kim J, Coradetti ST, Kim Y-M, Gao Y, Yaegashi J, Zucker JD, Munoz N, Zink EM, Burnum-Johnson KE, Baker SE (2021). Multi-omics driven metabolic network reconstruction and analysis of lignocellulosic carbon utilization in *Rhodosporidium toruloides*. Front Bioeng Biotechnol.

[31] Tiukova IA, Prigent S, Nielsen J, Sandgren M, Kerkhoven EJ (2019). Genome-scale model of *Rhodotorula toruloides* metabolism. Biotechnol Bioeng.

[32] Dinh HV, Suthers PF, Chan SHJ, Shen Y, Xiao T, Deewan A, Jagtap SS, Zhao H, Rao CV, Rabinowitz JD, Maranas CD (2019). A comprehensive genome-scale model for *Rhodosporidium toruloides* IFO0880 accounting for functional genomics and phenotypic data. Metabolic Eng Commun.

[33] Nora LC, Wehrs M, Kim J, Cheng J-F, Tarver A, Simmons BA, Magnuson J, Harmon-Smith M, Silva-Rocha R, Gladden JM (2019). A toolset of constitutive promoters for metabolic engineering of *Rhodosporidium toruloides*. Microb Cell Fact.

[34] Wang Y, Lin X, Zhang S, Sun W, Ma S, Zhao ZK (2016). Cloning and evaluation of different constitutive promoters in the oleaginous yeast *Rhodosporidium toruloides*. Yeast.

[35] Liu Y, Yap SA, Koh CMJ, Ji L (2016). Developing a set of strong intronic promoters for robust metabolic engineering in oleaginous Rhodotorula (*Rhodosporidium*) yeast species. Microb Cell Fact.

[36] Guo X, Bai Z, Zhang Y, Zhao H, Shi S (2023). Mining and application of constitutive promoters from *Rhodosporidium toruloides*. AMB Express.

[37] Liu Y, Koh CMJ, Ngoh ST, Ji L (2015). Engineering an efficient and tight d-amino acid-inducible gene expression system in *Rhodosporidium*/*Rhodotorula* species. Microb Cell Fact.

[38] Johns A, Love J, Aves SJ (2016). Four inducible promoters for controlled gene expression in the oleaginous yeast *Rhodotorula toruloides*. Front Microbiol.

[39] Bommareddy RR, Sabra W, Zeng A-P (2017). Glucose-mediated regulation of glycerol uptake in *Rhodosporidium toruloides*: Insights through transcriptomic analysis on dual substrate fermentation. Eng Life Sci.

[40] Qi F, Shen P, Hu R, Xue T, Jiang X, Qin L, Chen Y, Huang J (2020). Carotenoids and lipid production from *Rhodosporidium toruloides* cultured in tea waste hydrolysate. Biotechnol Biofuels.

[41] Zheng X, Hu R, Chen D, Chen J, He W, Huang L, Lin C, Chen H, Chen Y, Zhu J (2021). Lipid and carotenoid production by the *Rhodosporidium toruloides* mutant in cane molasses. Biores Technol.

[42] Protzko RJ, Hach CA, Coradetti ST, Hackhofer MA, Magosch S, Thieme N, Geiselman GM, Arkin AP, Skerker JM, Dueber JE, Benz JP (2019). Genomewide and enzymatic analysis reveals efficient D-Galacturonic acid metabolism in the basidiomycete yeast *Rhodosporidium toruloides*. mSystems.

[43] Qvirist L, Mierke F, Vazquez Juarez R, Andlid T (2022). Screening of xylose utilizing and high lipid producing yeast strains as a potential candidate for industrial application. BMC Microbiol.

[44] Hu J, Ji L (2016). Draft genome sequences of *Rhodosporidium toruloides* strains ATCC 10788 and ATCC 10657 with compatible mating types. Genome Announc.

[45] Sambles C, Middelhaufe S, Soanes D, Kolak D, Lux T, Moore K, Matoušková P, Parker D, Lee R, Love J, Aves SJ (2017). Genome sequence of the oleaginous yeast Rhodotorula toruloides strain CGMCC 2.1609. Genomics Data.

[46] Akada R, Kai J, Yamashita I, Miyakawa T, Fukui S (1989). Genomic organization of multiple genes coding for rhodotorucine A, a lipopeptide mating pheromone of the basidiomycetous yeast *Rhodosporidium toruloides*. Arch Microbiol.

[47] Bai Flagfeldt D, Siewers V, Huang L, Nielsen J (2009). Characterization of chromosomal integration sites for heterologous gene expression in *Saccharomyces cerevisiae*. Yeast.

[48] Coelho MA, Rosa A, Rodrigues N, Fonseca Á, Gonçalves P (2008). Identification of Mating Type Genes in the Bipolar Basidiomycetous Yeast *Rhodosporidium toruloides*: first insight into the MAT Locus structure of the *Sporidiobolales*. Eukaryot Cell.

[49] Bonturi N, Pinheiro MJ, de Oliveira PM, Rusadze E, Eichinger T, Liudžiūtė G, De Biaggi JS, Brauer A, Remm M, Miranda EA (2022). Development of a dedicated golden gate assembly platform (RtGGA) for *Rhodotorula toruloides*. Metabolic Eng Commun.

[50] Tkach JM, Yimit A, Lee AY, Riffle M, Costanzo M, Jaschob D, Hendry JA, Ou J, Moffat J, Boone C (2012). Dissecting DNA damage response pathways by analysing protein localization and abundance changes during DNA replication stress. Nat Cell Biol.

[51] Mendes-Ferreira A, del Olmo M, García-Martínez J, Jiménez-Martí E, Leão C, Mendes-Faia A, Pérez-Ortín JE (2007). *Saccharomyces cerevisiae* signature genes for predicting nitrogen deficiency during alcoholic fermentation. Appl Environ Microbiol.

[52] Koplin A, Preissler S, Ilina Y, Koch M, Scior A, Erhardt M, Deuerling E (2010). A dual function for chaperones SSB–RAC and the NAC nascent polypeptide–associated complex on ribosomes. J Cell Biol.

[53] Pomraning KR, Kim Y-M, Nicora CD, Chu RK, Bredeweg EL, Purvine SO, Hu D, Metz TO, Baker SE (2016). Multi-omics analysis reveals regulators of the response to nitrogen limitation in *Yarrowia lipolytica*. BMC Genomics.

[54] Mierke F, Brink DP, Norbeck J, Siewers V, Andlid T (2023). Functional genome annotation and transcriptome analysis of* Pseudozyma hubeiensis* BOT-O, an oleaginous yeast that utilizes glucose and xylose at equal rates. Fungal Gene Biol.

[55] Elfeky N, Elmahmoudy M, Zhang Y, Guo J, Bao Y (2019). Lipid and carotenoid production by *Rhodotorula glutinis* with a combined cultivation mode of nitrogen, sulfur, and aluminium stress. Appl Sci.

[56] Ratledge C, Wynn JP (2002). The biochemistry and molecular biology of lipid accumulation in oleaginous microorganisms. Adv Appl Microbiol.

[57] Liu X, Liu M, Zhang J, Chang Y, Cui Z, Ji B, Nielsen J, Qi Q, Hou J (2022). Mapping of nonhomologous end joining-mediated integration facilitates genome-scale trackable mutagenesis in *Yarrowia lipolytica*. ACS Synth Biol.

[58] Liu Y, Koh CMJ, Sun L, Hlaing MM, Du M, Peng N, Ji L (2013). Characterization of glyceraldehyde-3-phosphate dehydrogenase gene Rt GPD1 and development of genetic transformation method by dominant selection in oleaginous yeast *Rhodosporidium toruloides*. Appl Microbiol Biotechnol.

[59] Braunwald T, Schwemmlein L, Graeff-Hönninger S, French WT, Hernandez R, Holmes WE, Claupein W (2013). Effect of different C/N ratios on carotenoid and lipid production by *Rhodotorula glutinis*. Appl Microbiol Biotechnol.

[60] Andlid T, Larsson C, Liljenberg C, Marison I, Gustafsson L (1995). Enthalpy content as a function of lipid accumulation in *Rhodotorula glutinis*. Appl Microbiol Biotechnol.

[61] Andrews S (2010). FastQC: a quality control tool for high throughput sequence data.

[62] Wick RR, Judd LM, Gorrie CL, Holt KE (2017). Completing bacterial genome assemblies with multiplex MinION sequencing. Microbial Geno.

[63] De Coster W, D’Hert S, Schultz DT, Cruts M, Van Broeckhoven C (2018). NanoPack: visualizing and processing long-read sequencing data. Bioinformatics.

[64] Li H (2016). Minimap and miniasm: fast mapping and *de novo* assembly for noisy long sequences. Bioinformatics.

[65] Li H (2018). Minimap2: pairwise alignment for nucleotide sequences. Bioinformatics.

[66] Koren S, Walenz BP, Berlin K, Miller JR, Bergman NH, Phillippy AM (2017). Canu: scalable and accurate long-read assembly via adaptive k-mer weighting and repeat separation. Genome Res.

[67] Kolmogorov M, Yuan J, Lin Y, Pevzner PA (2019). Assembly of long, error-prone reads using repeat graphs. Nat Biotechnol.

[68] Shafin K, Pesout T, Lorig-Roach R, Haukness M, Olsen HE, Bosworth C, Armstrong J, Tigyi K, Maurer N, Koren S (2020). Nanopore sequencing and the Shasta toolkit enable efficient *de novo* assembly of eleven human genomes. Nature Biotechnol.

[69] Mikheenko A, Prjibelski A, Saveliev V, Antipov D, Gurevich A (2018). Versatile genome assembly evaluation with QUAST-LG. Bioinformatics.

[70] Vaser R, Sović I, Nagarajan N, Šikić M (2017). Fast and accurate *de novo* genome assembly from long uncorrected reads. Genome Res.

[71] Loman NJ, Quick J, Simpson JT (2015). A complete bacterial genome assembled *de novo* using only nanopore sequencing data. Nat Methods.

[72] Li H, Durbin R (2009). Fast and accurate short read alignment with Burrows-Wheeler transform. Bioinformatics.

[73] Li H, Handsaker B, Wysoker A, Fennell T, Ruan J, Homer N, Marth G, Abecasis G, Durbin R (2009). The sequence alignment/map format and SAMtools. Bioinformatics.

[74] Marçais G, Delcher AL, Phillippy AM, Coston R, Salzberg SL, Zimin A (2018). MUMmer4: a fast and versatile genome alignment system. PLoS Comput Biol.

[75] Zimin AV, Marçais G, Puiu D, Roberts M, Salzberg SL, Yorke JA (2013). The MaSuRCA genome assembler. Bioinformatics.

[76] Zimin AV, Salzberg SL (2020). The genome polishing tool POLCA makes fast and accurate corrections in genome assemblies. PLoS Comput Biol.

[77] Ewels P, Magnusson M, Lundin S, Käller M (2016). MultiQC: summarize analysis results for multiple tools and samples in a single report. Bioinformatics.

[78] Li H: seqtk: Toolkit for processing sequences in FASTA/Q formats. 2012.

[79] Krueger F: Trim galore v0.6.3. A wrapper tool around Cutadapt and FastQC to consistently apply quality and adapter trimming to FastQ files. Zenodo. https://zenodo.org/record/7598955#.ZC2EP_ZBxPY; 2019.

[80] Martin M (2011). Cutadapt removes adapter sequences from high-throughput sequencing reads. EMBnet J.

[81] Kim D, Paggi JM, Park C, Bennett C, Salzberg SL (2019). Graph-based genome alignment and genotyping with HISAT2 and HISAT-genotype. Nat Biotechnol.

[82] Wang L, Wang S, Li W (2012). RSeQC: quality control of RNA-seq experiments. Bioinformatics.

[83] Pertea M, Pertea GM, Antonescu CM, Chang T-C, Mendell JT, Salzberg SL (2015). StringTie enables improved reconstruction of a transcriptome from RNA-seq reads. Nat Biotechnol.

[84] Holt C, Yandell M (2011). MAKER2: an annotation pipeline and genome-database management tool for second-generation genome projects. BMC Bioinform.

[85] Coradetti ST, Pinel D, Geiselman GM, Ito M, Mondo SJ, Reilly MC, Cheng Y-F, Bauer S, Grigoriev IV, Gladden JM (2018). Functional genomics of lipid metabolism in the oleaginous yeast* Rhodosporidium toruloides*. Elife.

[86] Paul D, Magbanua Z, Arick M, French T, Bridges SM, Burgess SC, Lawrence ML (2014). Genome sequence of the oleaginous yeast *Rhodotorula glutinis* ATCC 204091. Genome Announc.

[87] Flynn JM, Hubley R, Goubert C, Rosen J, Clark AG, Feschotte C, Smit AF (2020). RepeatModeler2 for automated genomic discovery of transposable element families. Proc Natl Acad Sci.

[88] Bao W, Kojima KK, Kohany O (2015). Repbase update, a database of repetitive elements in eukaryotic genomes. Mob DNA.

[89] Hubley R, Finn RD, Clements J, Eddy SR, Jones TA, Bao W, Smit AFA, Wheeler TJ (2016). The Dfam database of repetitive DNA families. Nucleic Acids Res.

[90] Haas B: TransposonPSI: an application of PSI-Blast to mine (retro-) transposon ORF homologies. In *Broad Institute, Cambridge, MA, USA. *2007.

[91] Altschul SF, Gish W, Miller W, Myers EW, Lipman DJ (1990). Basic local alignment search tool. J Mol Biol.

[92] Campbell MS, Law M, Holt C, Stein JC, Moghe GD, Hufnagel DE, Lei J, Achawanantakun R, Jiao D, Lawrence CJ (2014). MAKER-P: a tool kit for the rapid creation, management, and quality control of plant genome annotations. Plant Physiol.

[93] Korf I (2004). Gene finding in novel genomes. BMC Bioinform.

[94] Lomsadze A, Burns PD, Borodovsky M (2014). Integration of mapped RNA-Seq reads into automatic training of eukaryotic gene finding algorithm. Nucleic Acids Res.

[95] Stanke M, Keller O, Gunduz I, Hayes A, Waack S, Morgenstern B (2006). AUGUSTUS: ab initio prediction of alternative transcripts. Nucleic Acids Res.

[96] Brůna T, Hoff KJ, Lomsadze A, Stanke M, Borodovsky M (2021). BRAKER2: automatic eukaryotic genome annotation with GeneMark-EP+ and AUGUSTUS supported by a protein database. NAR Geno Bioinform.

[97] Hoff KJ, Lomsadze A, Borodovsky M, Stanke M: Whole-genome annotation with BRAKER. In* Gene Prediction.* Springer; 2019: 65–95. 10.1007/978-1-4939-9173-0_5 PMC6635606 31020555

[98] Buchfink B, Xie C, Huson DH (2015). Fast and sensitive protein alignment using DIAMOND. Nat Methods.

[99] Eilbeck K, Moore B, Holt C, Yandell M (2009). Quantitative measures for the management and comparison of annotated genomes. BMC Bioinform.

[100] Seppey M, Manni M, Zdobnov EM, Kollmar M (2019). BUSCO: assessing genome assembly and annotation completeness. Gene Prediction.

[101] UniProt Consortium (2019). UniProt: a worldwide hub of protein knowledge. Nucleic Acids Res.

[102] Jones P, Binns D, Chang H-Y, Fraser M, Li W, McAnulla C, McWilliam H, Maslen J, Mitchell A, Nuka G (2014). InterProScan 5: genome-scale protein function classification. Bioinformatics.

[103] Dainat J, Hereñú D, Pucholt P: AGAT: Another Gff Analysis Toolkit to handle annotations in any GTF. Zenodo. https://zenodo.org/record/4637977; 2021.

[104] Emms DM, Kelly S (2019). OrthoFinder: phylogenetic orthology inference for comparative genomics. Genome Biol.

[105] Liao Y, Smyth GK, Shi W (2014). featureCounts: an efficient general purpose program for assigning sequence reads to genomic features. Bioinformatics.

[106] Love MI, Huber W, Anders S (2014). Moderated estimation of fold change and dispersion for RNA-seq data with DESeq2. Genome Biol.

[107] R Core Team (2022). R a language and environment for statistical computing. R foundation for statistical computing.

[108] Blighe K, Rana S, Lewis M: EnhancedVolcano: Publication-ready volcano plots with enhanced colouring and labeling. *R package version* 2019, 1.

[109] Goris J, Konstantinidis KT, Klappenbach JA, Coenye T, Vandamme P, Tiedje JM (2007). DNA–DNA hybridization values and their relationship to whole-genome sequence similarities. Int J Syst Evol Microbiol.

[110] Pritchard L, Glover RH, Humphris S, Elphinstone JG, Toth IK (2016). Genomics and taxonomy in diagnostics for food security: soft-rotting enterobacterial plant pathogens. Anal Methods.

[111] Camacho C, Coulouris G, Avagyan V, Ma N, Papadopoulos J, Bealer K, Madden TL (2009). BLAST+: architecture and applications. BMC Bioinform.

[112] Edgar RC (2004). MUSCLE: multiple sequence alignment with high accuracy and high throughput. Nucleic Acids Res.

[113] Bodenhofer U, Bonatesta E, Horejš-Kainrath C, Hochreiter S (2015). msa: an R package for multiple sequence alignment. Bioinformatics.

[114] Inoue H, Nojima H, Okayama H (1990). High efficiency transformation of *Escherichia coli* with plasmids. Gene.

[115] Bergkessel M, Guthrie C: Chapter Twenty Five - Colony PCR. In *Methods in Enzymology. Volume* 529. Edited by Lorsch J: Academic Press; 2013: 299–309. 10.1016/B978-0-12-418687-3.00025-2 24011056

[116] Sambrook J, Russell DW (2001). Molecular cloning: a laboratory manual.

[117] Smid M, Coebergh van den Braak RRJ, van de Werken HJG, van Riet J, van Galen A, de Weerd V, van der Vlugt-Daane M, Bril SI, Lalmahomed ZS, Kloosterman WP (2018). Gene length corrected trimmed mean of M-values (GeTMM) processing of RNA-seq data performs similarly in intersample analyses while improving intrasample comparisons. BMC Bioinform.

