# Supplementary material for: Expanding the genetic toolbox of Rhodotorula toruloides by identification and validation of six novel promoters induced or repressed under nitrogen starvation
Source: Microb Cell Fact. 2023 Aug 19;22:160. doi: 10.1186/s12934-023-02175-2 (PMC10440040; doi:10.1186/s12934-023-02175-2)
Supplement: Supplementary file 1 — Additional file 1: Supplementary figures and tables. [file 12934_2023_2175_MOESM1_ESM.pdf]

Supplemental File S1 for

Expanding the genetic toolbox of *Rhodotorula toruloides* by identification and validation of six novel promoters induced or repressed under nitrogen starvation

Daniel P. Brink[†,a,b], Friederike Mierke[†,a,c], Joakim Norbeck[a], Verena Siewers[a,]* and Thomas Andlid[c]

[a] Systems and Synthetic Biology, Department of Life Sciences, Chalmers University of Technology, Gothenburg, Sweden

[b] Applied Microbiology, Department of Chemistry, Lund University, Lund, Sweden

[c] Food and Nutrition Science, Department of Life Sciences, Chalmers University of Technology, Gothenburg, Sweden

[†]These authors contributed equally to this work.

*Corresponding author: siewers@chalmers.se

Supplemental Figures

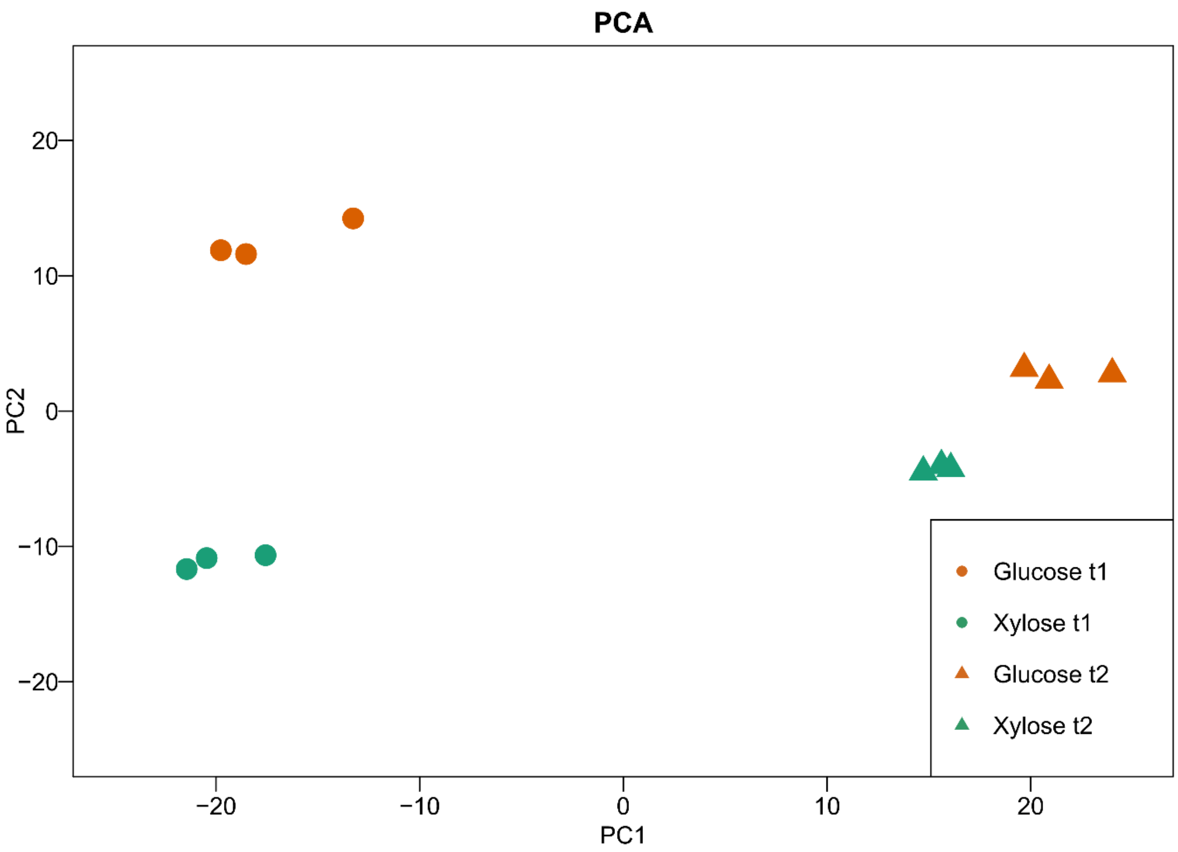

Supplemental Figure S1. Principal Component Analysis of the twelve *R. toruloides* BOT-A2 RNAseq samples. Biological replicates are represented by the same colour and shape.

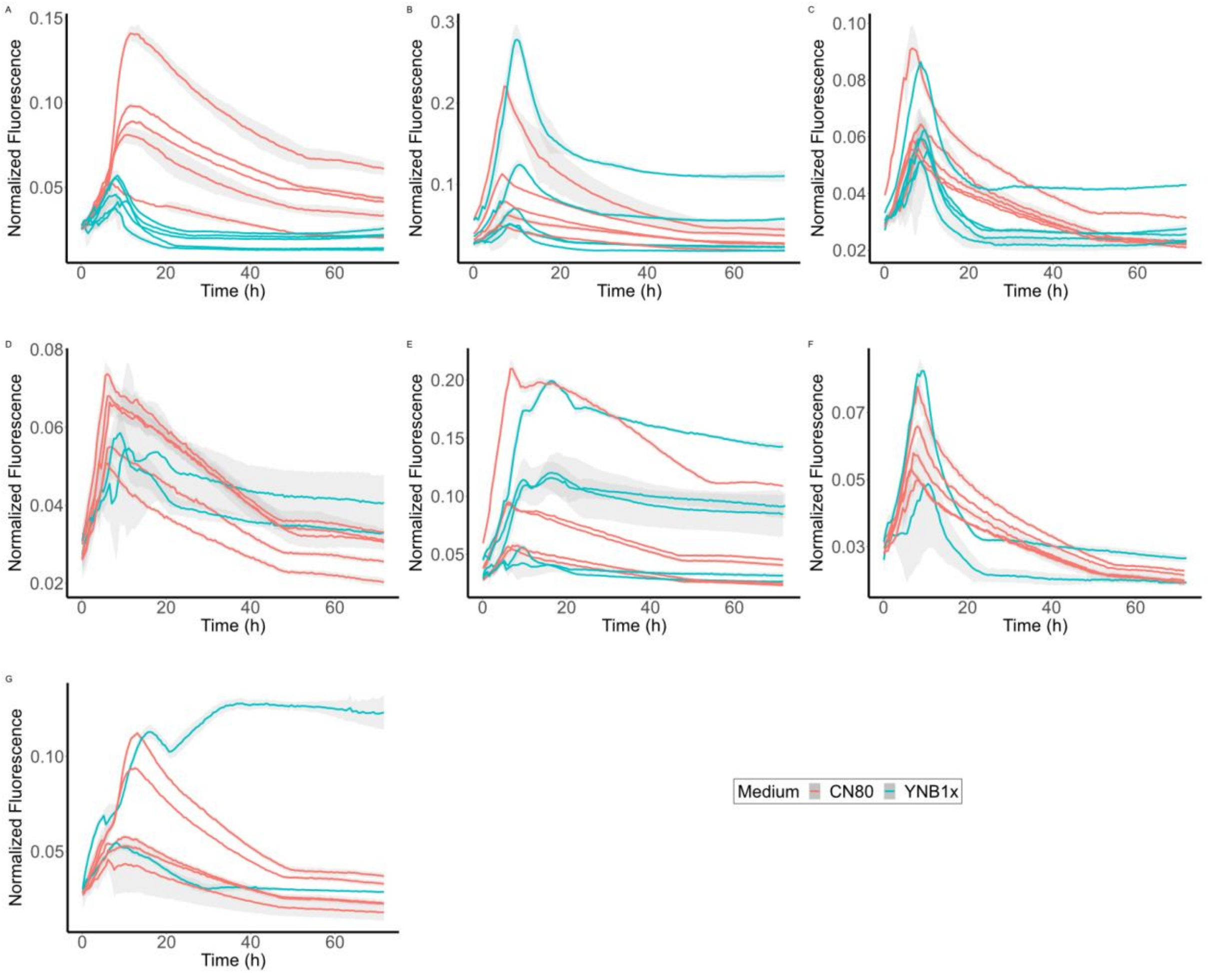

Supplemental Figure S2. BioLector assessment of strains Up2 (A), Up4 (B), Up5 (C), Up6 (D), Up7 (E), Up8 (F), and Up9 (G). The experiment was performed in triplicates and with CN80 (red) as GFP induction medium and YNB1x (blue) as GFP repression medium. Fluorescence was normalised to biomass measured as scattered light. Errors are presented as shadows.

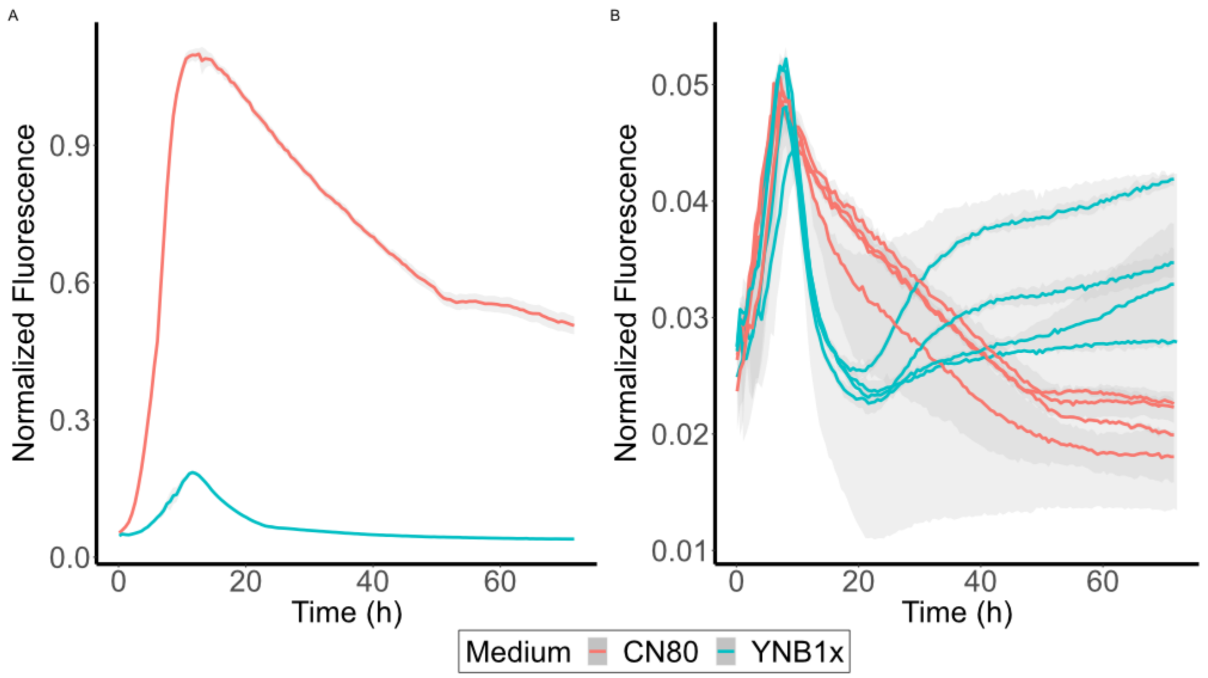

Supplemental Figure S3. BioLector assessment of Up3 strains. A shows Up3e, and B shows UP3a-d. The experiment was performed in triplicates and with CN80 (red) as GFP induction medium and YNB1x (blue) as GFP repression medium. Fluorescence was normalised to biomass measured as scattered light. Errors are presented as shadows.

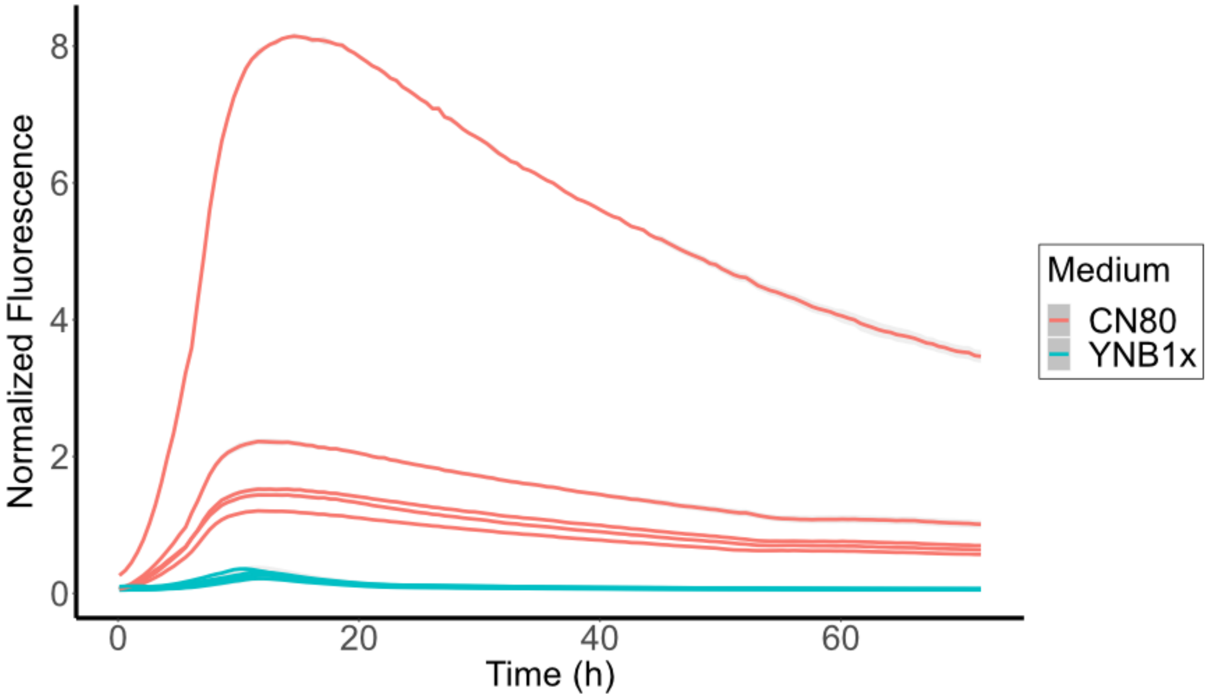

Supplemental Figure S4. BioLector assessment of Up10 strains. The experiment was performed in triplicates and with CN80 (red) as GFP induction medium and YNB1x (blue) as GFP repression medium. Fluorescence was normalised to biomass measured as scattered light. Errors are presented as shadows.

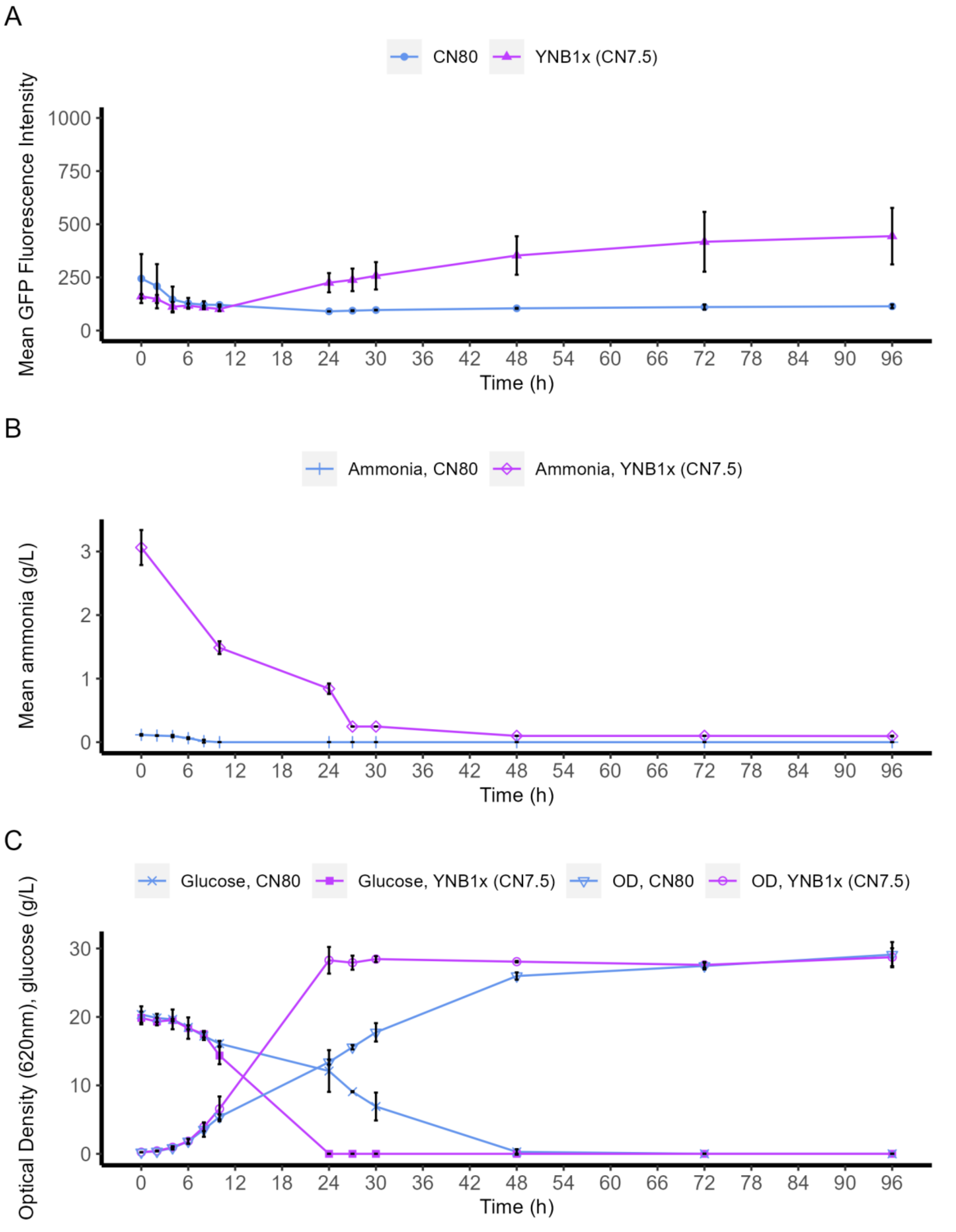

Supplemental Figure S5. Control experiment for the promoter analyses, using the wild-type strain BOT-A2 that lacks GFP. Cultivation was performed in shake flasks with 20 g/L glucose in either YNB medium (YNB1x), or in nitrogen-limited YNB (CN80). A: autofluorescence on the GFP channel (533/30 nm) B. ammonium consumption profile. C: biomass formation (OD_{620nm}) and glucose consumption profiles. Error bars refer to standard deviation from two biological replicates.

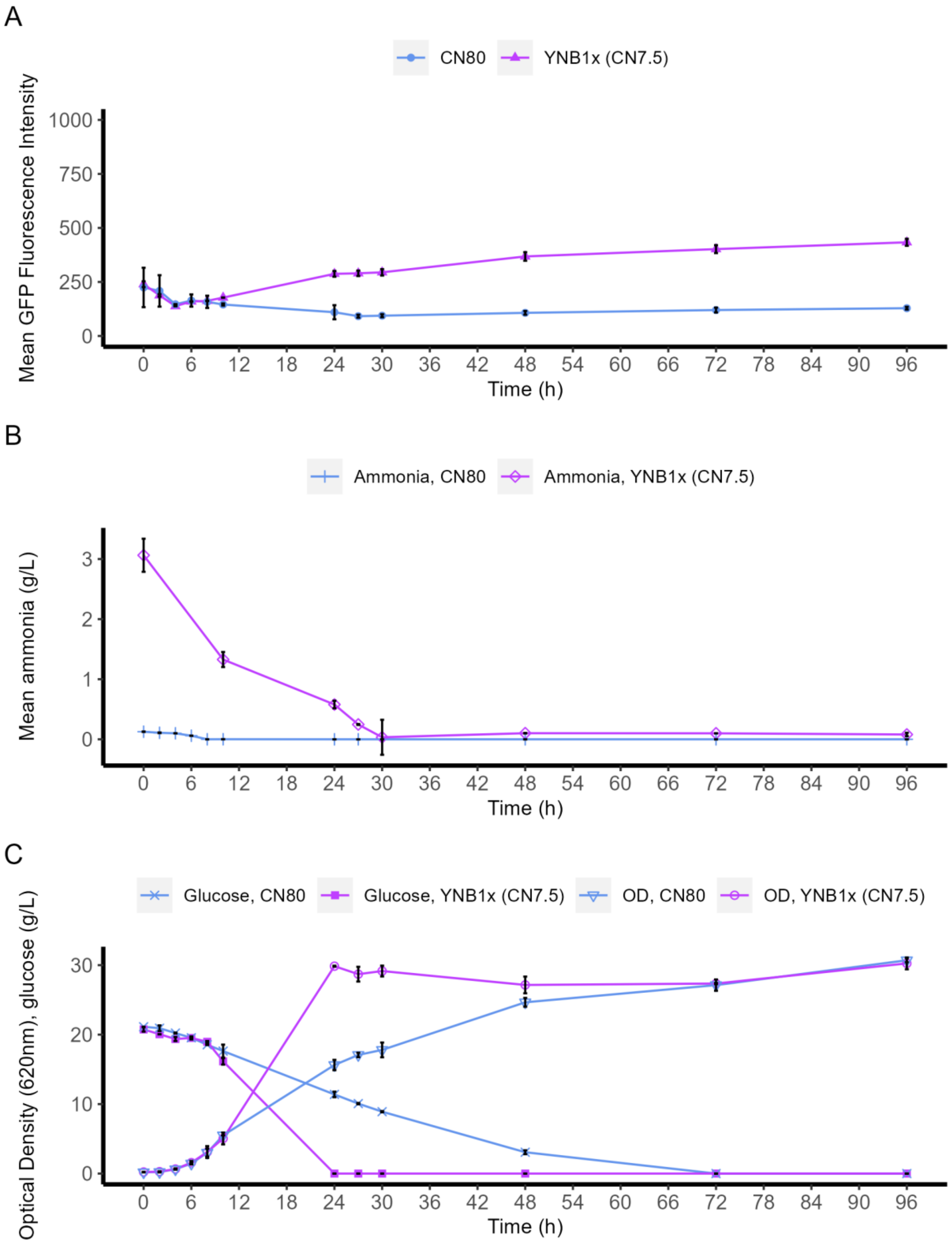

Supplemental Figure S6. Control experiment for the promoter analyses, using the strain CTRL1a wild-type containing a GFP cassette without any promoter. Cultivation was performed in shake flasks with 20 g/L glucose in either YNB medium (YNB1x), or in nitrogen-limited YNB (CN80). A: fluorescence on the GFP channel (533/30 nm) B. ammonium consumption profile. C: biomass formation (OD_{620nm}) and glucose consumption profiles. Error bars refer to standard deviation from two biological replicates.

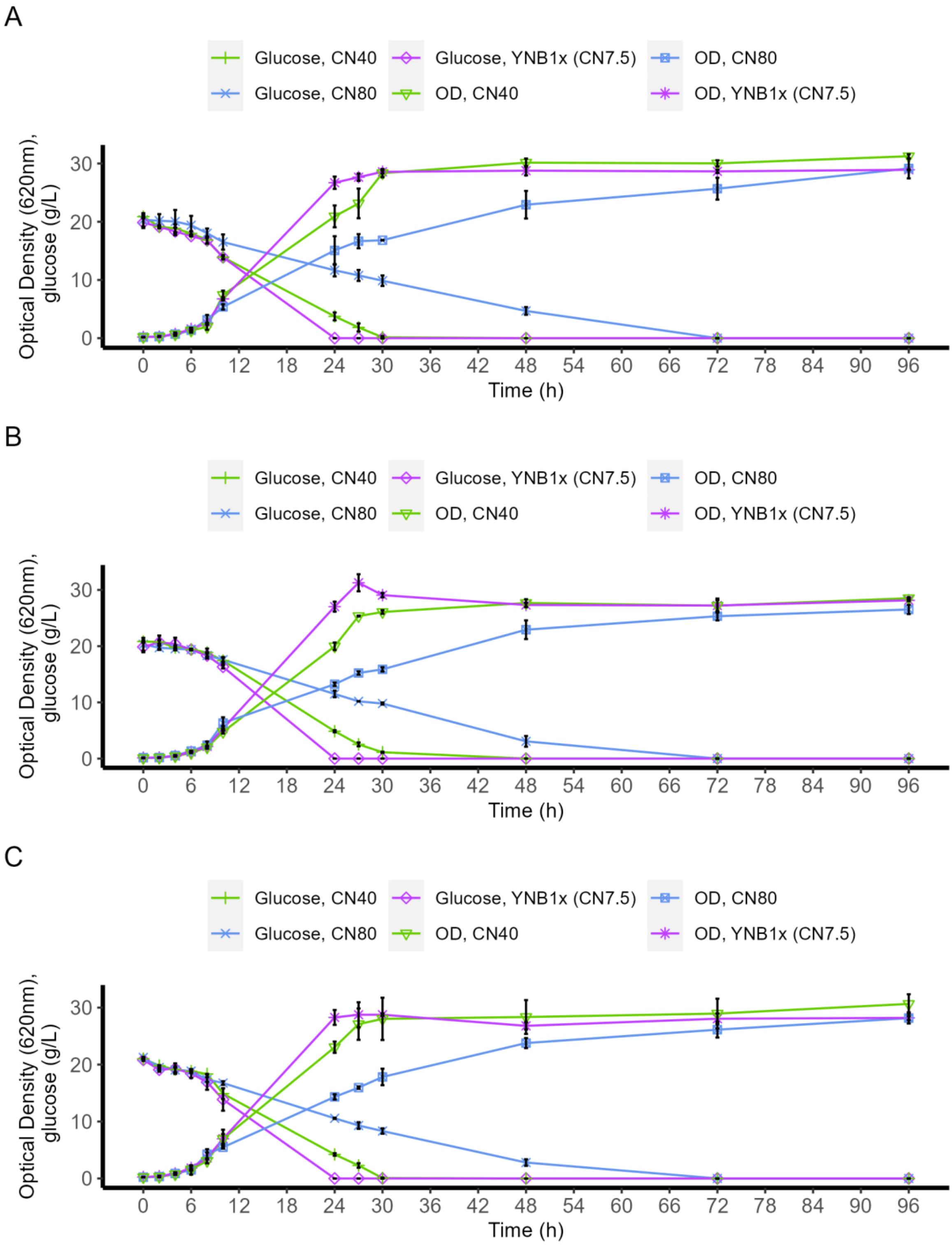

Supplemental Figure S7. Biomass formation (OD_{620nm}) and glucose consumption profiles OD_{620nm} from shake-flask cultivations with 20 g/L glucose in either YNB medium (YNB1x), or in nitrogen-limited YNB (CN80, and CN40). A: UP1a; B: UP3e; C: UP10a. Error bars refer to standard deviation from two biological replicates.

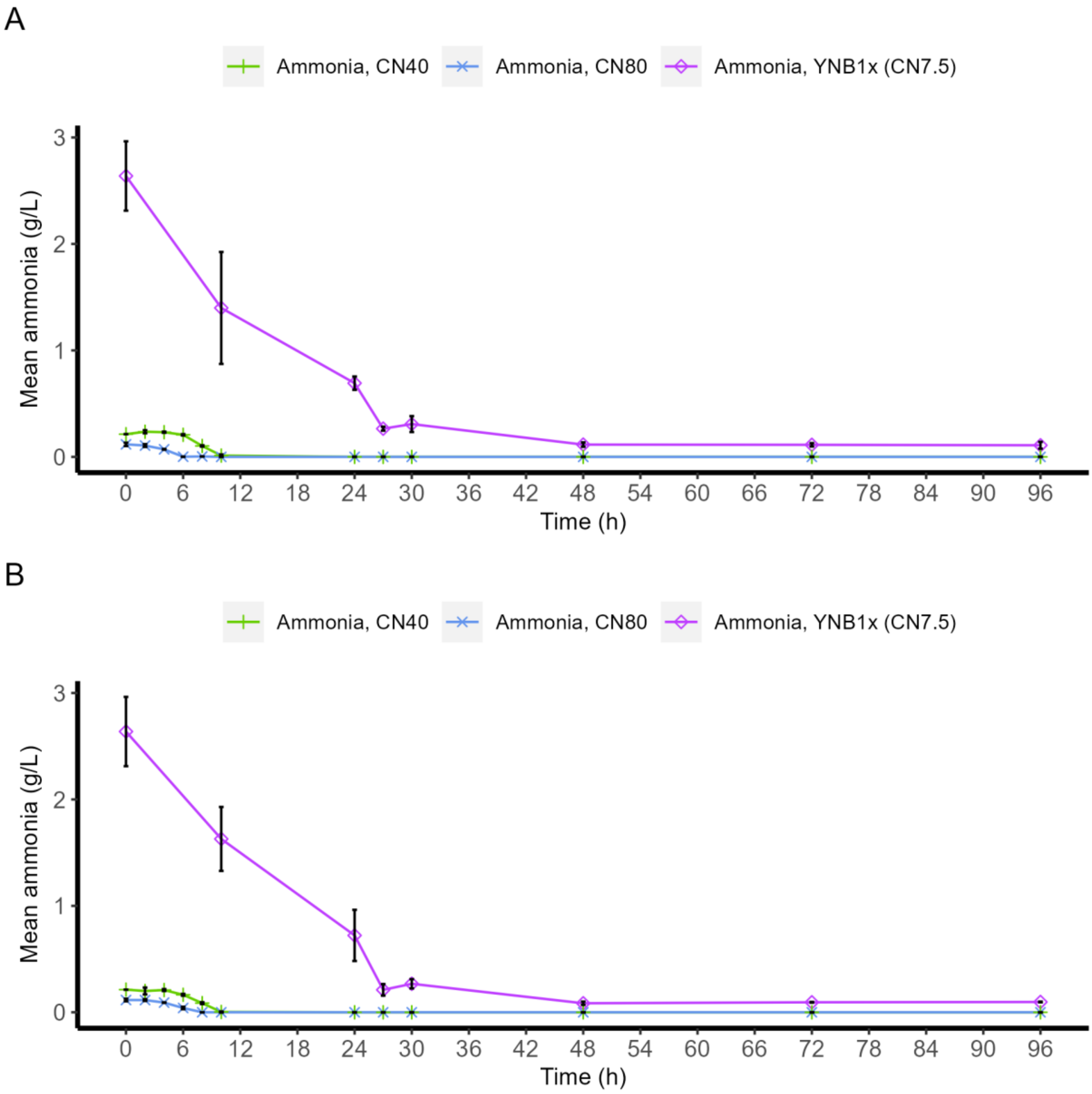

Supplemental Figure S8. Ammonia consumption profiles of A: strain UP3e; B: strain UP10a. The strains were evaluated in shake flasks with 20 g/L glucose in either YNB medium (YNB1x), or in nitrogen-limited YNB (CN80, and CN40). Error bars refer to standard deviation from two biological replicates.

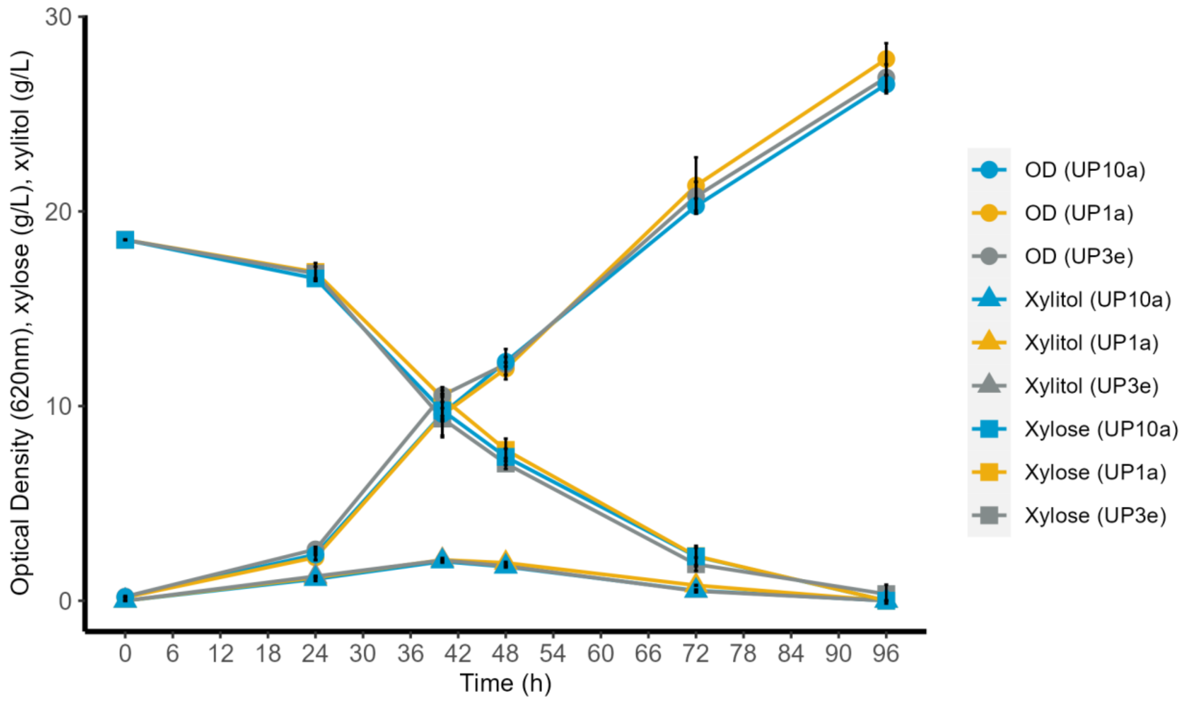

Supplemental Figure S9. Biomass formation (OD_{620nm}), xylose consumption and xylitol metabolism profiles from shake-flask cultivations with 20 g/L xylose in in nitrogen-limited YNB (CN80). Error bars refer to standard deviation from two biological replicates.

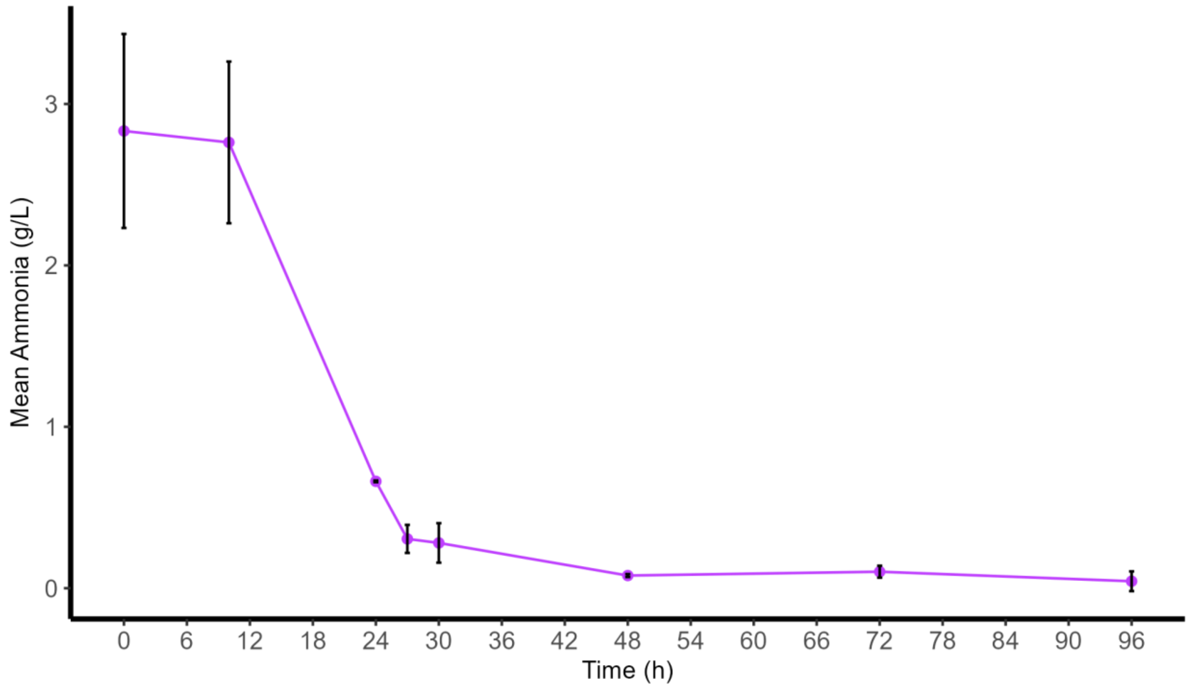

Supplemental Figure S10. Ammonia consumption profile of strain UP1a in shake flasks in non-nitrogen limited YNB medium with 20 g/L glucose (YNB1x). Error bars refer to standard deviation from two biological replicates.

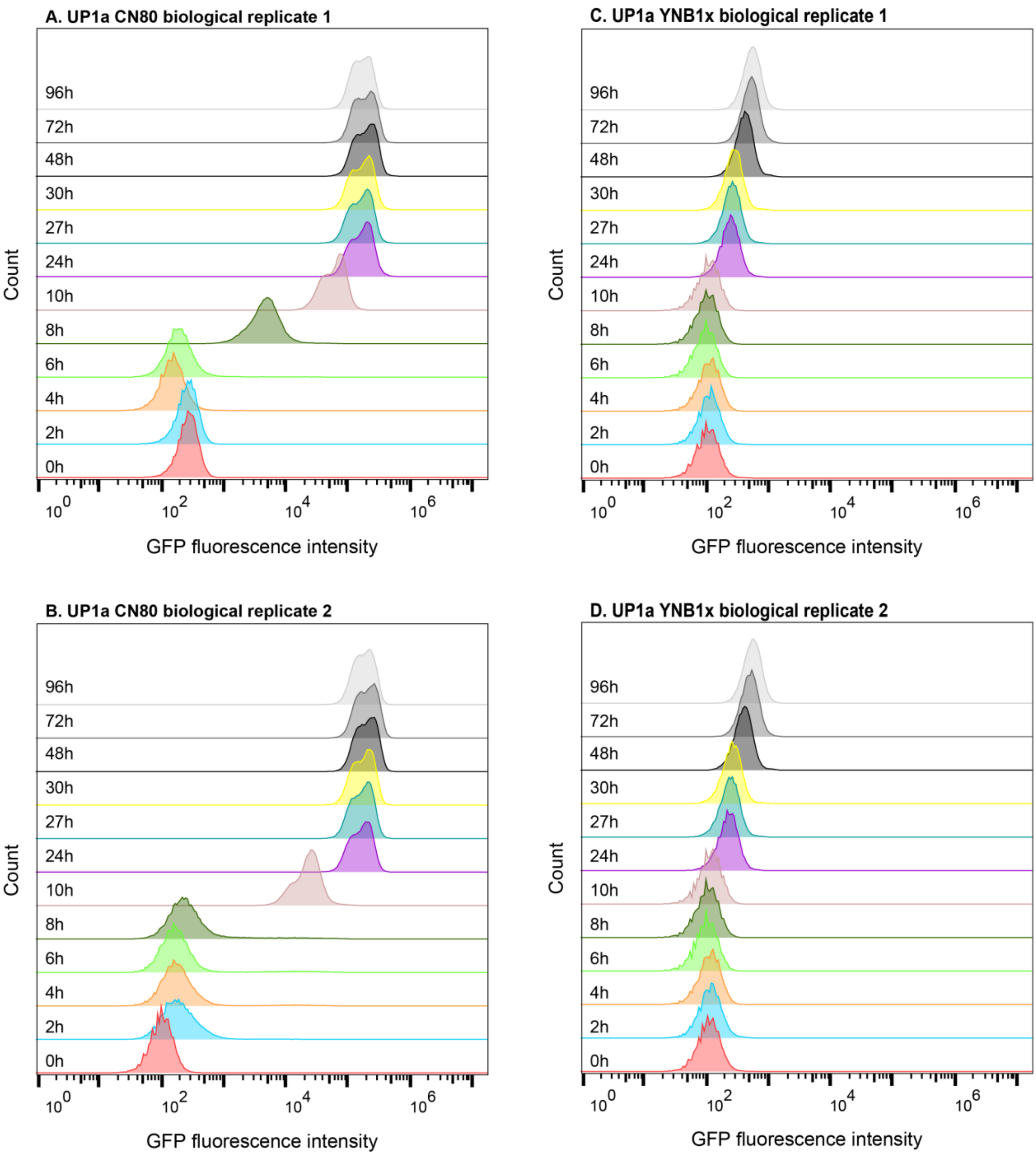

Supplemental Figure S11. Histograms from the flow cytometry analysis of strain Up1a, cultivated in shake-flasks with 20 g/L glucose. A and B: two biological replicates from cultivation in nitrogen-limited YNB medium (CN80). C and D: two biological replicates from cultivation in non-nitrogen limited YNB medium (YNB1x).

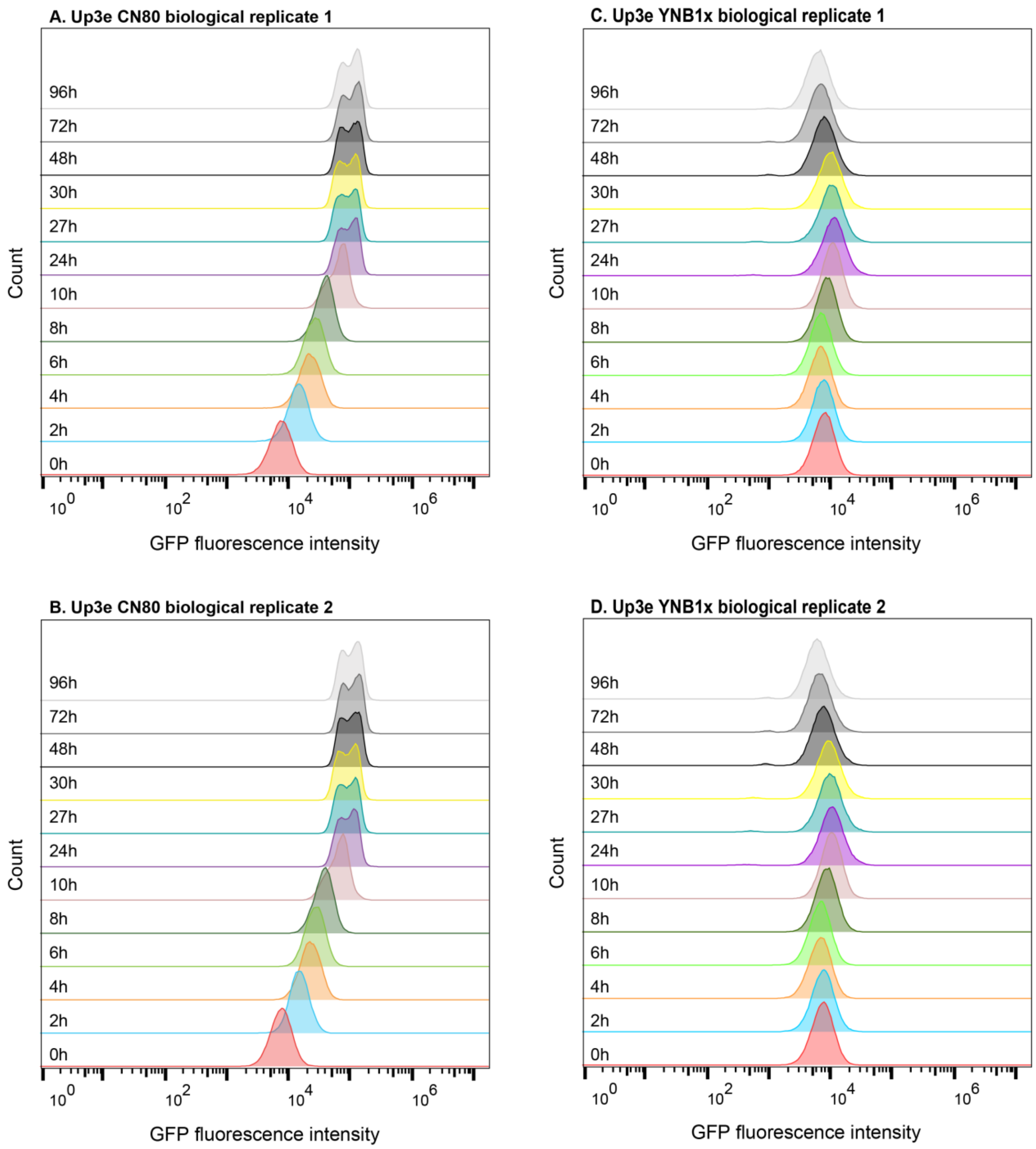

Supplemental Figure S12. Histograms from the flow cytometry analysis of strain Up3e, cultivated in shake-flasks with 20 g/L glucose. A and B: two biological replicates from cultivation in nitrogen-limited YNB medium (CN80). C and D: two biological replicates from cultivation in non-nitrogen limited YNB medium (YNB1x).

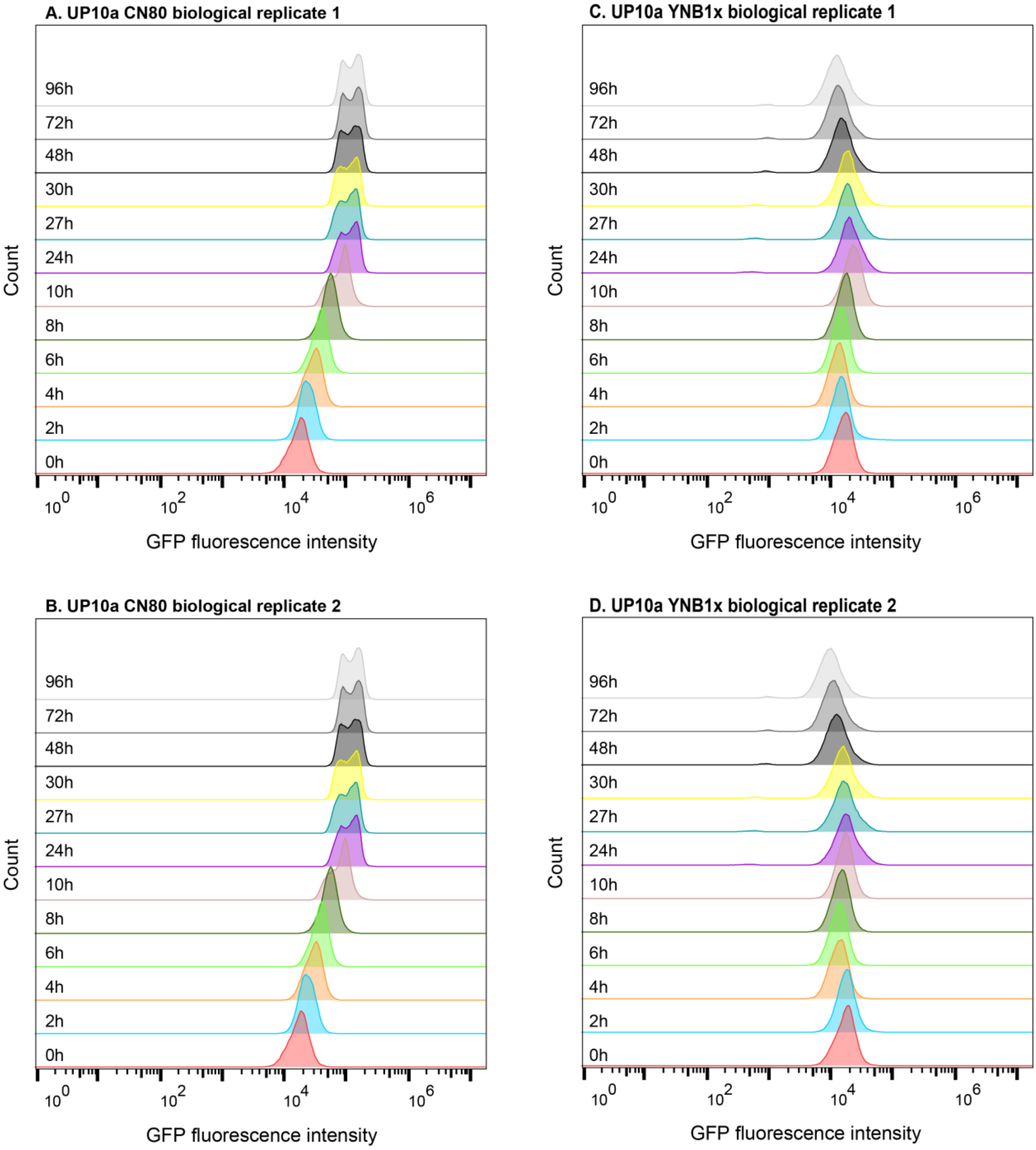

Supplemental Figure S13. Histograms from the flow cytometry analysis of strain Up10a, cultivated in shake-flasks with 20 g/L glucose. A and B: two biological replicates from cultivation in nitrogen-limited YNB medium (CN80). C and D: two biological replicates from cultivation in non-nitrogen limited YNB medium (YNB1x).

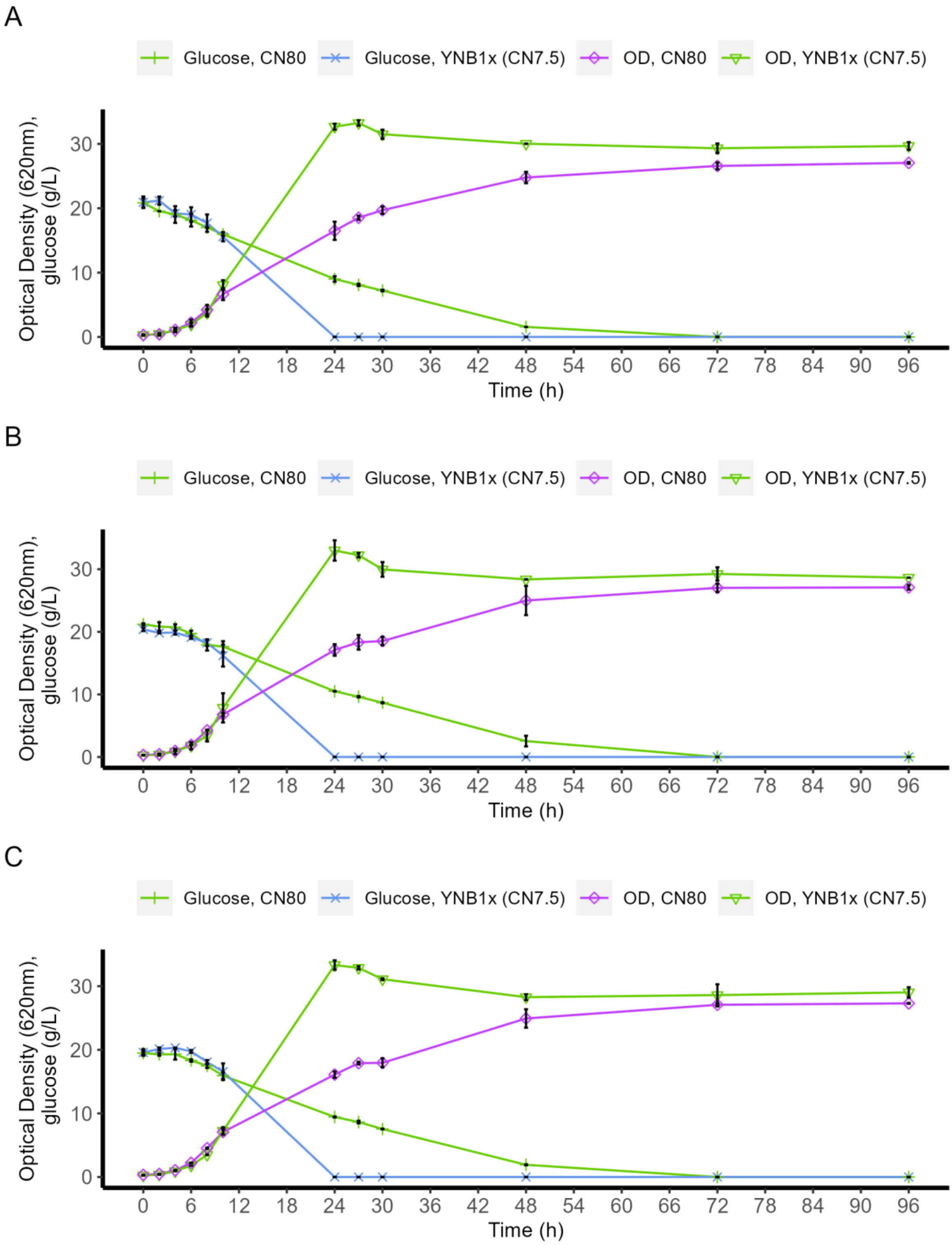

Supplemental Figure S14. Biomass formation (OD_{620nm}) and glucose consumption profiles OD_{620nm} from shake-flask cultivations with 20 g/L glucose in either YNB medium (YNB1x), or in nitrogen-limited YNB (CN80, and CN40). A: DN1a; B: DN2a; C: DN5c. Error bars refer to standard deviation from two biological replicates.

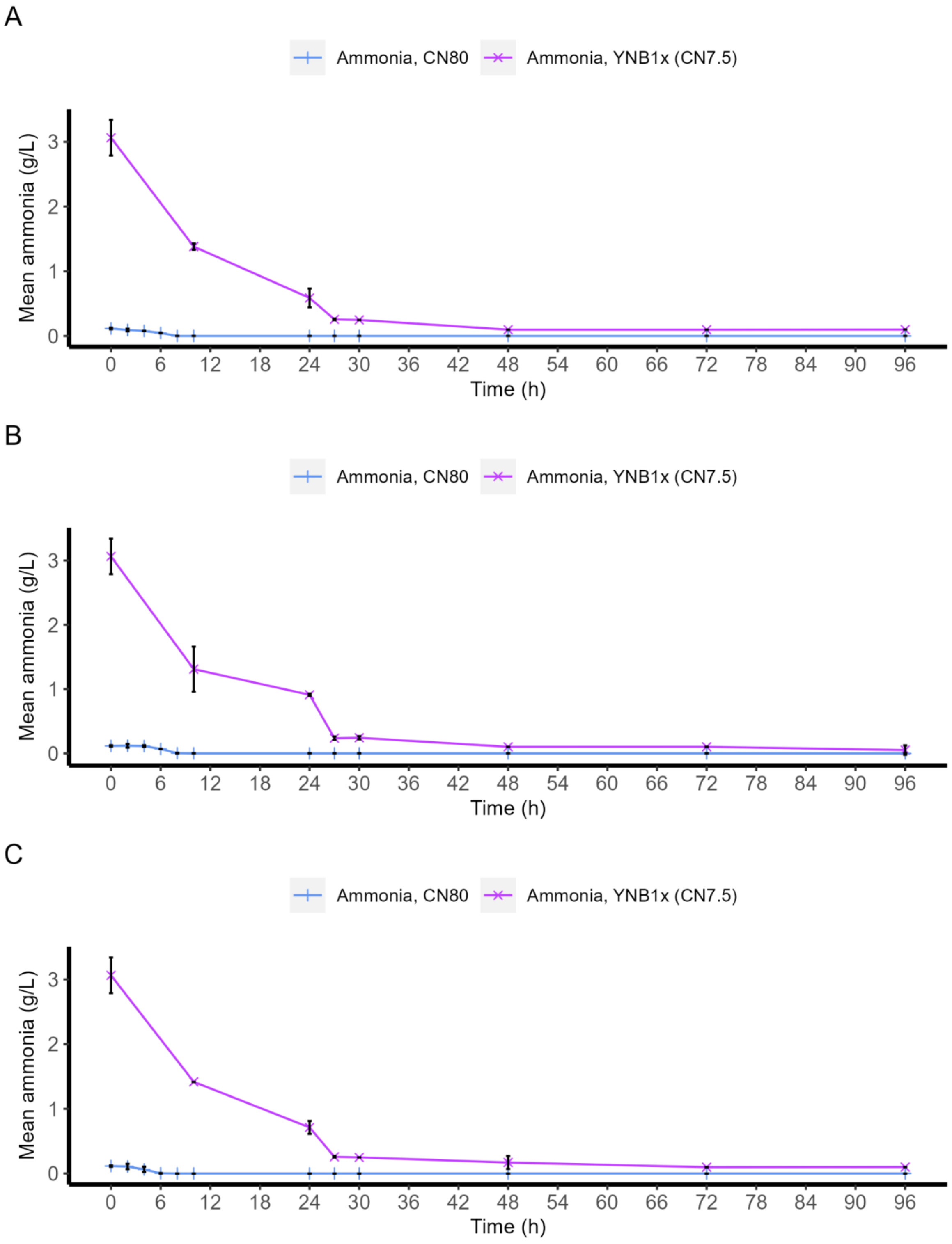

Supplemental Figure S15. Ammonia consumption profiles of A: strain DN1a; B: strain DN2a; B: strain DN5c. The strains were evaluated in shake flasks with 20 g/L glucose in either YNB medium (YNB1x), or in nitrogen-limited YNB (CN80, and CN40). Error bars refer to standard deviation from two biological replicates.

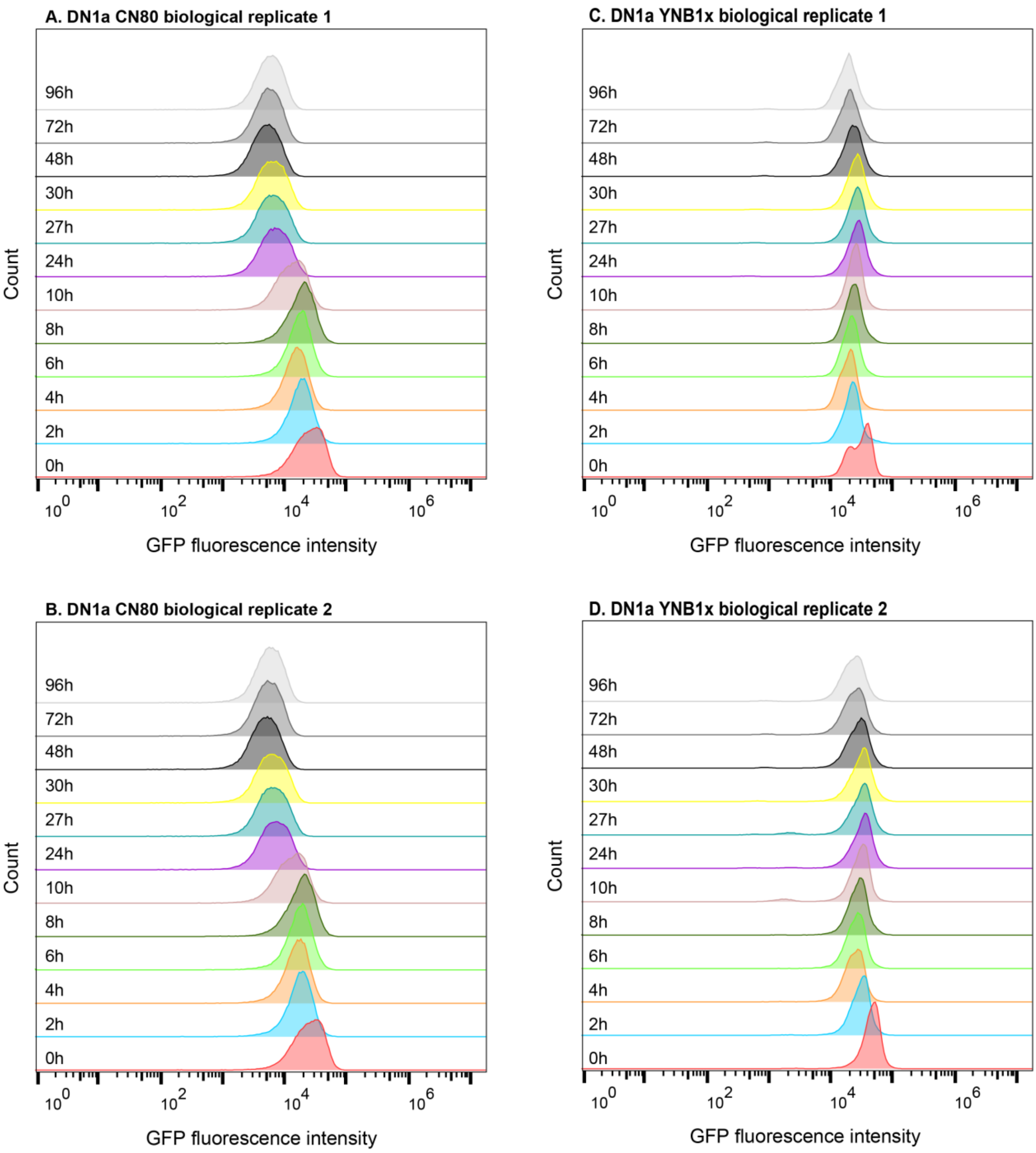

Supplemental Figure S16. Histograms from the flow cytometry analysis of strain DN1a, cultivated in shake-flasks with 20 g/L glucose. A and B: two biological replicates from cultivation in nitrogen-limited YNB medium (CN80). C and D: two biological replicates from cultivation in non-nitrogen limited YNB medium (YNB1x).

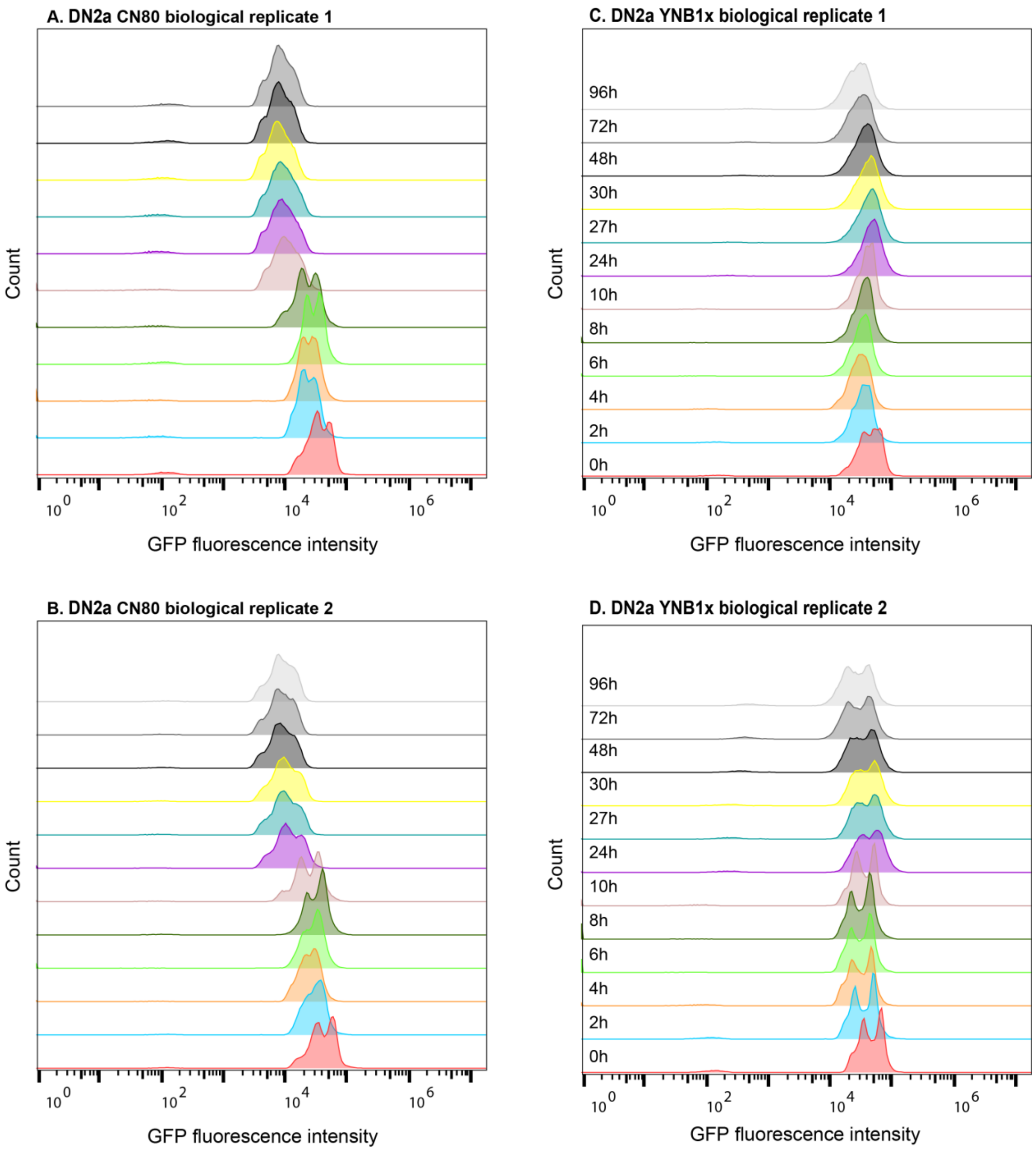

Supplemental Figure S17. Histograms from the flow cytometry analysis of strain DN2a, cultivated in shake-flasks with 20 g/L glucose. A and B: two biological replicates from cultivation in nitrogen-limited YNB medium (CN80). C and D: two biological replicates from cultivation in non-nitrogen limited YNB medium (YNB1x).

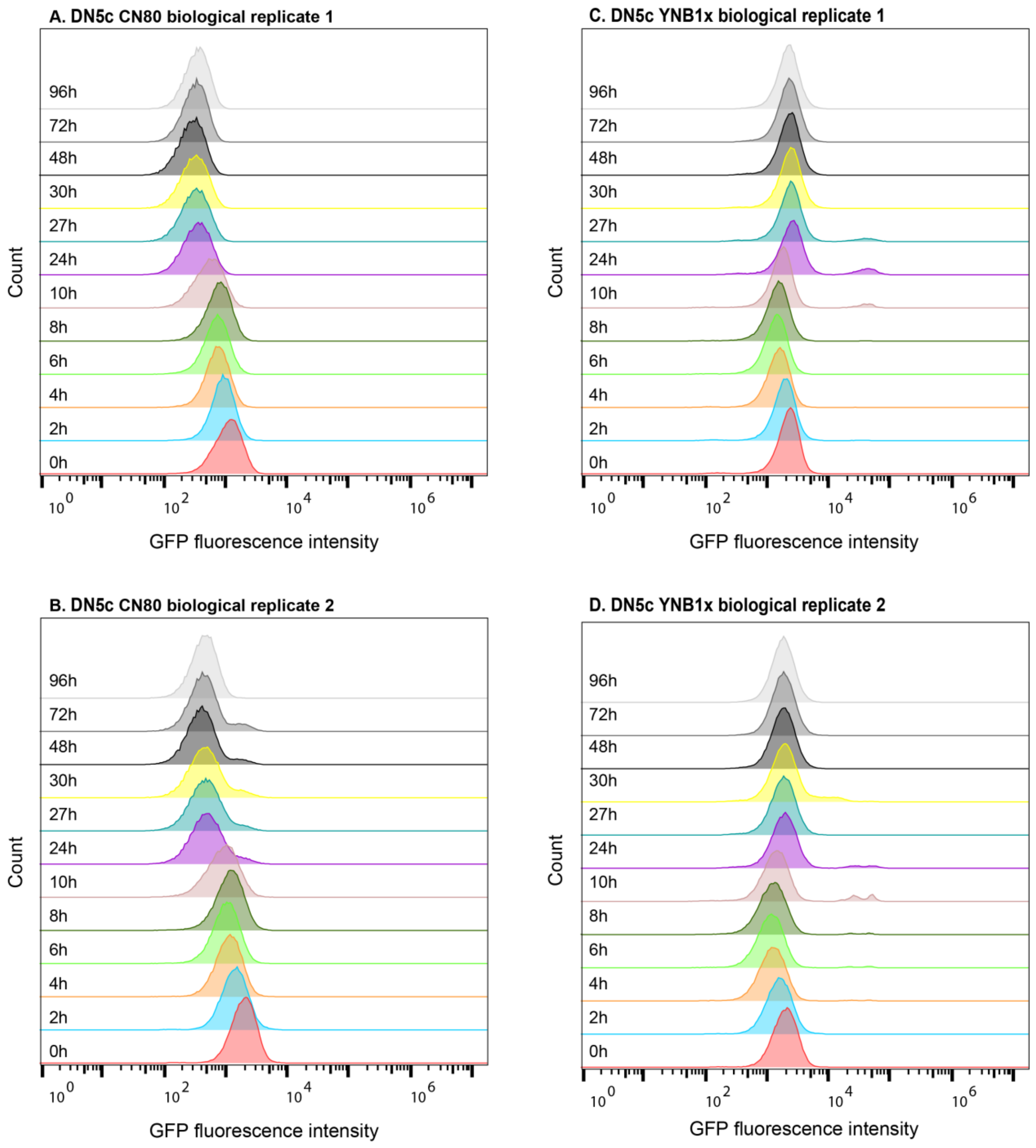

Supplemental Figure S18. Histograms from the flow cytometry analysis of strain DN5c, cultivated in shake-flasks with 20 g/L glucose. A and B: two biological replicates from cultivation in nitrogen-limited YNB medium (CN80). C and D: two biological replicates from cultivation in non-nitrogen limited YNB medium (YNB1x).

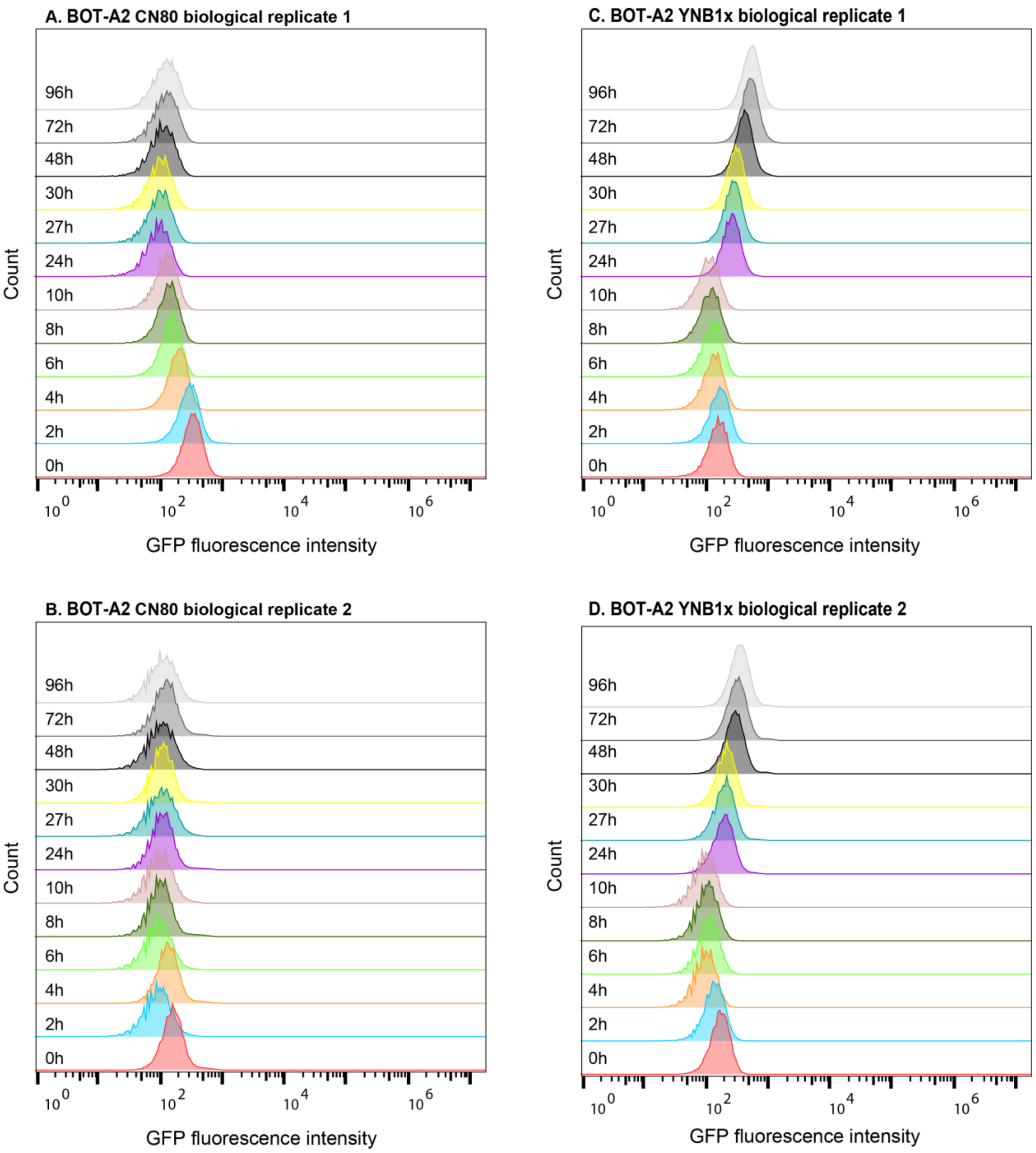

Supplemental Figure S19. Histograms from the flow cytometry analysis of the wild-type strain BOT-A2, cultivated in shake-flasks with 20 g/L glucose. A and B: two biological replicates from cultivation in nitrogen-limited YNB medium (CN80). C and D: two biological replicates from cultivation in non-nitrogen limited YNB medium (YNB1x).

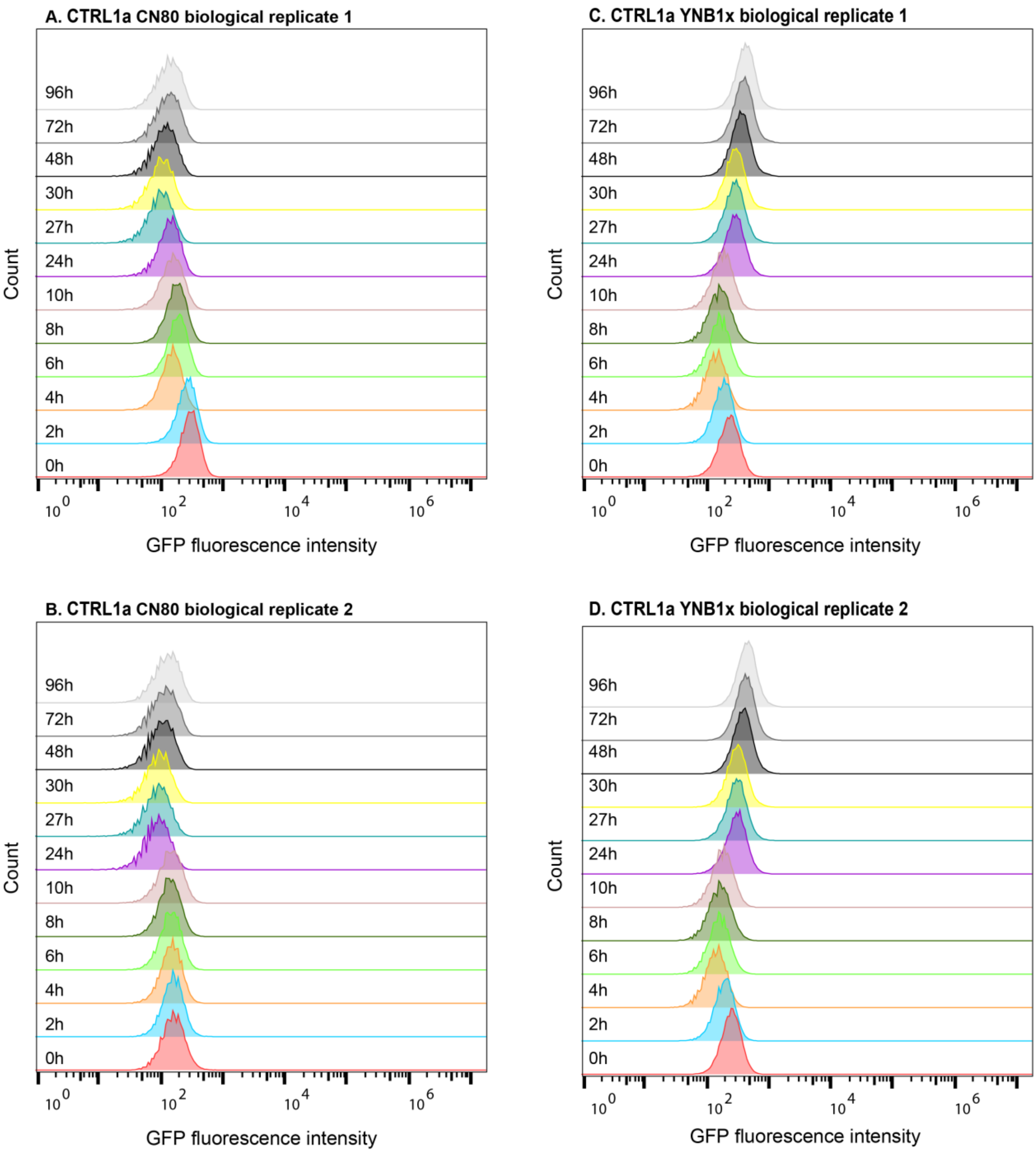

Supplemental Figure S20. Histograms from the flow cytometry analysis of the CTRL1a strain that contains a GFP cassette without a promoter, cultivated in shake-flasks with 20 g/L glucose. A and B: two biological replicates from cultivation in nitrogen-limited YNB medium (CN80). C and D: two biological replicates from cultivation in non-nitrogen limited YNB medium (YNB1x).

Supplemental Tables

Supplemental Table S1. Pheromone receptor analysis results using tblastn. The origin of M7X934 was strain NP11 and for G0T0M8 strain ATCC 204091.

Strain	Pheromone receptor Query (Uniprot accession)	% query coverage	Evalue	% identity	Contig	Coordinates in contig
**ATCC 10657**	A1 (M7X934)	54%	5,00E-21	28.39%	LNKU01000018.1	328835-329542
	A2 (G0T0M8)	100%	5,00E-165	76.82%	LNKU01000018.1	328643-329548; 329613-329978
**ATCC 10788**	A1 (M7X934)	95%	1,00E-129	79.51%	LNQQ01000012.1	302053-302667; 301608-301946; 301406-301606
	A2 (G0T0M8)	62%	9,00E-19	37.50%	LNQQ01000012.1	301629-301979; 302092-302412
**ATCC 204091**	A1 (M7X934)	54%	5,00E-21	28.39%	AEVR02000017.1	477807-477100
	A2 (G0T0M8)	100%	5,00E-165	76.82%	AEVR02000017.1	477999-477094; 477029-476664
**BOT-A2**	A1 (M7X934)	54%	2,00E-22	28.39%	utg000005l	478755-478048
	A2 (G0T0M8)	100%	2,00E-166	76.82%	utg000005l	478947-478042; 477977-477612
**CBS14**	A1 (M7X934)	95%	3,00E-129	79.51%	CAKLCE020000050.1	1600174-1600788; 1599729-1600067; 1599527-1599727
	A2 (G0T0M8)	62%	2,00E-18	37.50%	CAKLCE020000050.1	1599750-1600100; 1600213-1600533
**CCT 0783**	A1 (M7X934)	95%	2,00E-127	79.02%	JABGON010000578.1	1647-2261; 1202-1540; 1000-1200
	A1 (M7X934)	49%	9,00E-20	35.05%	JABGON010000455.1	20277-19987; 19876-19682; 20392-20312;
	A2 (G0T0M8)	100%	2,00E-117	51.42%	JABGON010000455.1	20584-19244;
	A2 (G0T0M8)	62%	4,00E-18	37.50%	JABGON010000578.1	1223-1573; 1686-2006
**CECT 1137**	A1 (M7X934)	95%	1,00E-129	79.51%	LK052954.1	50635-50021; 51080-50742; 51282-51082
	A2 (G0T0M8)	62%	9,00E-19	37.50%	LK052954.1	51059-50709; 50596-50276
**CGMCC.2**	A1 (M7X934)	95%	2,00E-129	79.51%	LKER01000158.1	52313-51699; 52758-52420; 52960-52760
	A2 (G0T0M8)	62%	2,00E-18	37.50%	LKER01000158.1	52737-52387; 52274-51954
**delta dao 1e**	A1 (M7X934)	54%	5,00E-21	28.39%	JAMRMT010000023.1	97089-96382
	A2 (G0T0M8)	100%	5,00E-165	76.82%	JAMRMT010000023.1	97281-96376; 96311-95946

**IFO 0559**	A1 (M7X934)	95%	1,00E-129	79.51%	LCTU01000114.1	10610-11224; 10165-10503; 9963-10163
	A2 (G0T0M8)	62%	1,00E-18	37.50%	LCTU01000114.1	10186-10536; 10649-10969
**IFO 0880**	A1 (M7X934)	54%	5,00E-21	28.39%	CWKI01000013.1	477730-477023
	A2 (G0T0M8)	100%	5,00E-165	76.82%	CWKI01000013.1	477922-477017; 476952-476587
**JCM 10020**	A1 (M7X934)	95%	1,00E-129	79.51%	BCIY01000019.1	52121-51507; 52566-52228; 52768-52568
	A2 (G0T0M8)	62%	1,00E-18	37.50%	BCIY01000019.1	52545-52195; 52082-51762
**JCM 10021**	A1 (M7X934)	54%	5,00E-21	28.39%	BCIZ01000012.1	299637-300344
	A2 (G0T0M8)	100%	5,00E-165	76.82%	BCIZ01000012.1	299445-300350; 300415-300780
**JCM 10049**	A1 (M7X934)	95%	1,00E-127	79.02%	BCJA01000015.1	301278-301892; 300833-301171; 300631-300831
	A2 (G0T0M8)	62%	2,00E-18	37.50%	BCJA01000015.1	300854-301204; 301317-301637
**JCM 24501**	A1 (M7X934)	50%	1,00E-20	36.08%	BCJE01000020.1	307130-307420; 307531-307722; 307006-307095
	A2 (G0T0M8)	95%	4,00E-158	68.34%	BCJE01000020.1	307564-308160; 307142-307456; 306825-306971; 307021-307092
**MTCC 457**	A1 (M7X934)	95%	1,00E-129	79.51%	AJMJ01000189.1	13670-14284; 13225-13563; 13023-13223
	A2 (G0T0M8)	62%	1,00E-18	37.50%	AJMJ01000189.1	13246-13596; 13709-14029
**NBRC 0880**	A1 (M7X934)	54%	5,00E-21	28.39%	LCTV02000013.1	477748-477041
	A2 (G0T0M8)	100%	5,00E-165	76.82%	LCTV02000013.1	477940-477035; 476970-476605
**NBRC 10032**	A1 (M7X934)	95%	2,00E-150	74.59%	BJWK01000015.1	186238-185696; 185589-185149; 185113-184970
	A2 (G0T0M8)	75%	5,00E-25	31.32%	BJWK01000015.1	186205-185660; 185550-185230
**NP11**	A1 (M7X934)	95%	1,00E-129	79.51%	NW_015622982.1	302151-302765; 301706-302044; 301504-301704
	A2 (G0T0M8)	62%	9,00E-19	37.50%	NW_015622982.1	301727-302077; 302190-302510
**VN1**	A1 (M7X934)	95%	3,00E-146	79.56%	SJTE01000146.1	4183-3641; 3534-3082; 3080-2922
	A2 (G0T0M8)	87%	9,00E-25	31.43%	SJTE01000146.1	4132-3608; 3492-3064
**Z1**	A1 (M7X934)	95%	2,00E-129	79.51%	JANQAS010000009.1	10006-10620; 9561-9899; 9359-9559

	A2 (G0T0M8)	73%	1,00E-18	37.50%	JANQAS010000009.1	9582-9932; 10045-10365; 9410-9559
**Z11**	A1 (M7X934)	95%	2,00E-129	79.51%	JAKRWS020000009.1	10006-10620; 9561-9899; 9359-9559
	A2 (G0T0M8)	73%	1,00E-18	37.50%	JAKRWS020000009.1	9582-9932; 10045-10365; 9410-9559

Supplemental Table S2. Key metrics from the BOT-A2 genome annotation process.

Gene model	Number of predicted genes (including tRNA)	Average length of the predicted CDSs (bp)[a]	Complete BUSCOs[b]	Fragmented BUSCOs	Missing BUSCOs
BOT-A2 assembly (before annotation)	-	-	1546 (87.7%)	41 (2.3%)	177 (10.0%)
Annotation round 01	7129	2713.22	1642 (93.1%)	17 (0.7%)	110 (6.2%)
Annotation round 02 (selected as the final model)	7001	2814.28	1703 (96.6%)	10 (0.6%)	51 (2.8%)
Annotation round 03	6968	2827.66	1702 (96.5%)	8 (0.5%)	54 (3.0%)

[a] The number of genes and the length of their coding sequences (CDSs) were identified by counting the *gene* features in the gff annotation file.

[b] Annotation completeness was assessed by screening the DNA sequences for evolutionary conserved Basidiomycota genes, so called BUSCOs (Benchmarking Universal Single-Copy Orthologs). The Basidiomycota BUSCO database used in this analysis contained 1764 BUSCOs.

Table S3. List of plasmids used in this study. The sequences for the promoters (RTBOTA2_00XXXXp) are available in Supplementary File S3.

**Plasmid**	**Relevant genotype**
pDB22	Rt*CAR2*_US-homology; *MEP2*p-Rt*EGFP*-At*NOS*t; *GPD1*p (RtCBS14) - kanMX-NcBetaTub_terminator; Rt*CAR2*_DS-homology
pDB23	pDB22; *MEP2*p::Eco72I
pDB43_2	pDB22; RTBOTA2_003877p-Rt*EGFP*-At*NOS*t
pDB44_3	pDB22; RTBOTA2_004847p-Rt*EGFP*-At*NOS*t
pDB45_2	pDB22; RTBOTA2_000480p-Rt*EGFP*-At*NOS*t
pDB46_2	pDB22; RTBOTA2_002184p-Rt*EGFP*-At*NOS*t
pDB47_2	pDB22; RTBOTA2_006908p-Rt*EGFP*-At*NOS*t
pDB48_2	pDB22; RTBOTA2_002391p-Rt*EGFP*-At*NOS*t
pDB49_2	pDB22; RTBOTA2_006709p-Rt*EGFP*-At*NOS*t
pDB50	pDB22; RTBOTA2_000568p-Rt*EGFP*-At*NOS*t
pDB51	pDB22; RTBOTA2_005111p-Rt*EGFP*-At*NOS*t
pDB52	pDB22; RTBOTA2_000530p-Rt*EGFP*-At*NOS*t
pDB53	pDB22; RTBOTA2_003356p-Rt*EGFP*-At*NOS*t
pDB55	pDB22; RTBOTA2_005449p-Rt*EGFP*-At*NOS*t
pDB56	pDB22; RTBOTA2_004071p-Rt*EGFP*-At*NOS*t
pDB57	pDB22; RTBOTA2_005360p-Rt*EGFP*-At*NOS*t
pDB58	pDB22; RTBOTA2_005958p-Rt*EGFP*-At*NOS*t

Supplemental Table S4. List of the PCR primers used in this study.

Primer name	Sequence	Comment
RtEGFP_1F_phos	ATGGCGGATCCGGTCTC GAA	Primer pair used to exchange MEP2p for ECO72I
RtCAR2_750R_Eco72I_phos	CACGTGCTCGCTGAACG CACCAAAGC	‘’
RTBOTA2_003877p_1F	AGCATTACAGCAAGCCG GAAC	Cloning of the 3877p promoter
RTBOTA2_003877p_1000R	GGTGAAAGTCGGGGAG GTCG	‘’
RTBOTA2_004847_R729_phos	GTGAGTCTGCTCGCTTG CCT	Cloning of the 4847p promoter
RTBOTA2_004847_R_phos	TGTGAATGCGCGTTGTG GGC	‘’
RTBOTA2_000480_F785_phos	GTGCGTTCTCGCTTCATG AGCTC	Cloning of the 0480p promoter
RTBOTA2_000480_R1_phos	TGTATGTCGTTTGATGTC GAGTAGC	‘’
RTBOTA2_002184_F584_phos	CTGCATGGCAAGCGCAA GC	Cloning of the 2184p promoter
RTBOTA2_002184_R1_phos	CGTCGGGTGTCGCGAGA TGAA	‘’
RTBOTA2_006908_F1000_phos	AGAGCAGTGTCTGAATA CTCTACGAC	Cloning of the 6908p promoter
RTBOTA2_006908_R1_phos	CGCAGCGTGTCTAGCAA GTCAG	‘’
RTBOTA2_002391_F759_phos	TGTTGTCTTGATGACCAT AAGGTTG	Cloning of the 2391p promoter
RTBOTA2_002391_R1_phos	GACTTATTGTCGGTGATG AAAATGC	‘’
RTBOTA2_006709_F710_phos	GCGAGGAGAGGCTGGA AAGG	Cloning of the 6709p promoter
RTBOTA2_006709_R1_phos	GGCAGTGTGCTGCCAAC GTC	‘’
RTBOTA2_000568_F723_phos	CTGTAACGCAGTCTCGG CGC	Cloning of the 0568p promoter
RTBOTA2_000568_R1_phos	GCTCGCTGCGAGTCCTG GC	‘’
RTBOTA2_005111_F_743_phos	GGAAGTCGGTCCTGGTG CTG	Cloning of the 5111p promoter
RTBOTA2_005111_R1_phos	GATGCAAGTGCTGCTGT TGGA	‘’
RTBOTA2_000530_F952_phos	CTGTACTGCTGTACGAA CAGCTTGC	Cloning of the 0530p promoter
RTBOTA2_000530_R1_phos	GGTTGCTGGACTGGGGG AAAG	‘’
RTBOTA2_003356_F1000_phos	ACAGAGACAACGGGAC GCACT	Cloning of the 0356p promoter

RTBOTA2_003356_R1_phos	CGTTCGAGGGGAAGGA GGGA	‘’
RTBOTA2_005449_F834_phos	CGTTCCAAACACAGCAA ACAGC	Cloning of the 5449p promoter
RTBOTA2_005449_R1_phos	GACCTCGTTGAAGGTTT GGCT	‘’
RTBOTA2_004071_F997_phos	TTCGGCTGCTTGACAAG TGGGAA	Cloning of the 4071p promoter
RTBOTA2_004071_R1_phos	CGCGAGCCAGTCAGTGA GC	‘’
RTBOTA2_005360_F997_phos	ATGGCTGACGACGAGGT TGTGA	Cloning of the 5360p promoter
RTBOTA2_005360_R1_phos	GGTCGGCGTTGGGTGGT GAG	‘’
RTBOTA2_005958_R957_phos	AACTAGACGAGGACCGC CTCGA	Cloning of the 5958p promoter
RTBOTA2_005958_R1_phos	CGTAGCTGTCTGGCACG CAC	‘’
RtCAR2_731F	GCTTTGGTGCGTTCAGC GAG	Amplification of the linear DNA fragments used for the transformation
RtCAR2_730R	CGCCTCGATCTGCTTGTT CGA	‘’
