# Supplementary material for: Expanding the genetic toolbox of Rhodotorula toruloides by identification and validation of six novel promoters induced or repressed under nitrogen starvation
Source: Microb Cell Fact. 2023 Aug 19;22:160. doi: 10.1186/s12934-023-02175-2 (PMC10440040; doi:10.1186/s12934-023-02175-2)
Supplement: Supplementary file 4 — Additional file 4. Multiple sequence alignment results are for the MEP2 promoter. [file 12934_2023_2175_MOESM4_ESM.pdf]

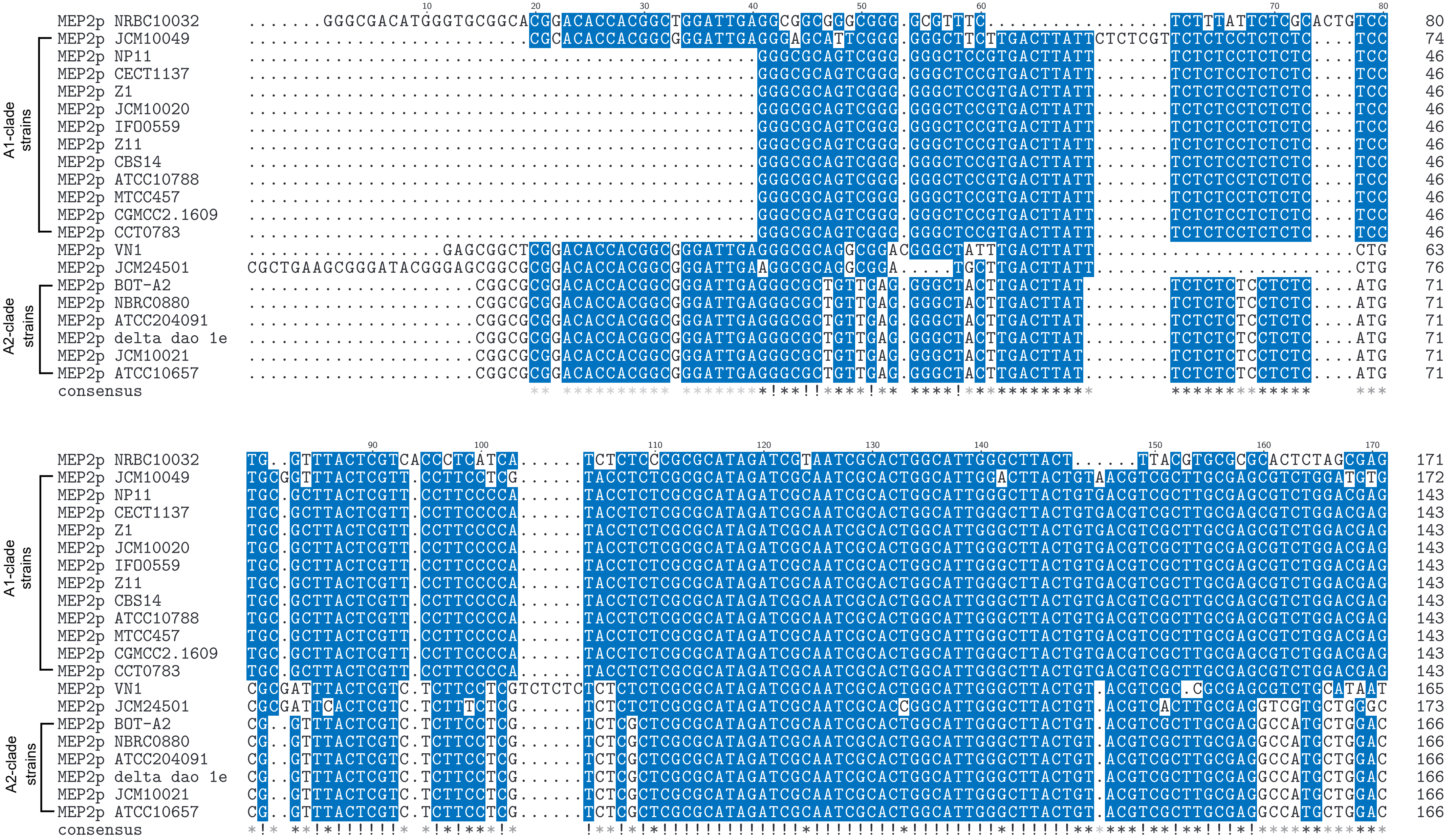

Multiple Sequence Alignment (MSA) of the putative MEP2p promoter sequences from all the currently available *R. toruloides* genomes. The candidate sequence from strain NP11 was identified as the 1000 kb directly upstream of the start codon of the gene, and this sequence was used as input for a nucleotide Blast analysis to identify the corresponding sequences in the other genomes. See the Materials and Methods section for details on the MSA. The brackets indicate the established clades from the Average Nucleotide Identity analysis (see Figure 1 in the main text).

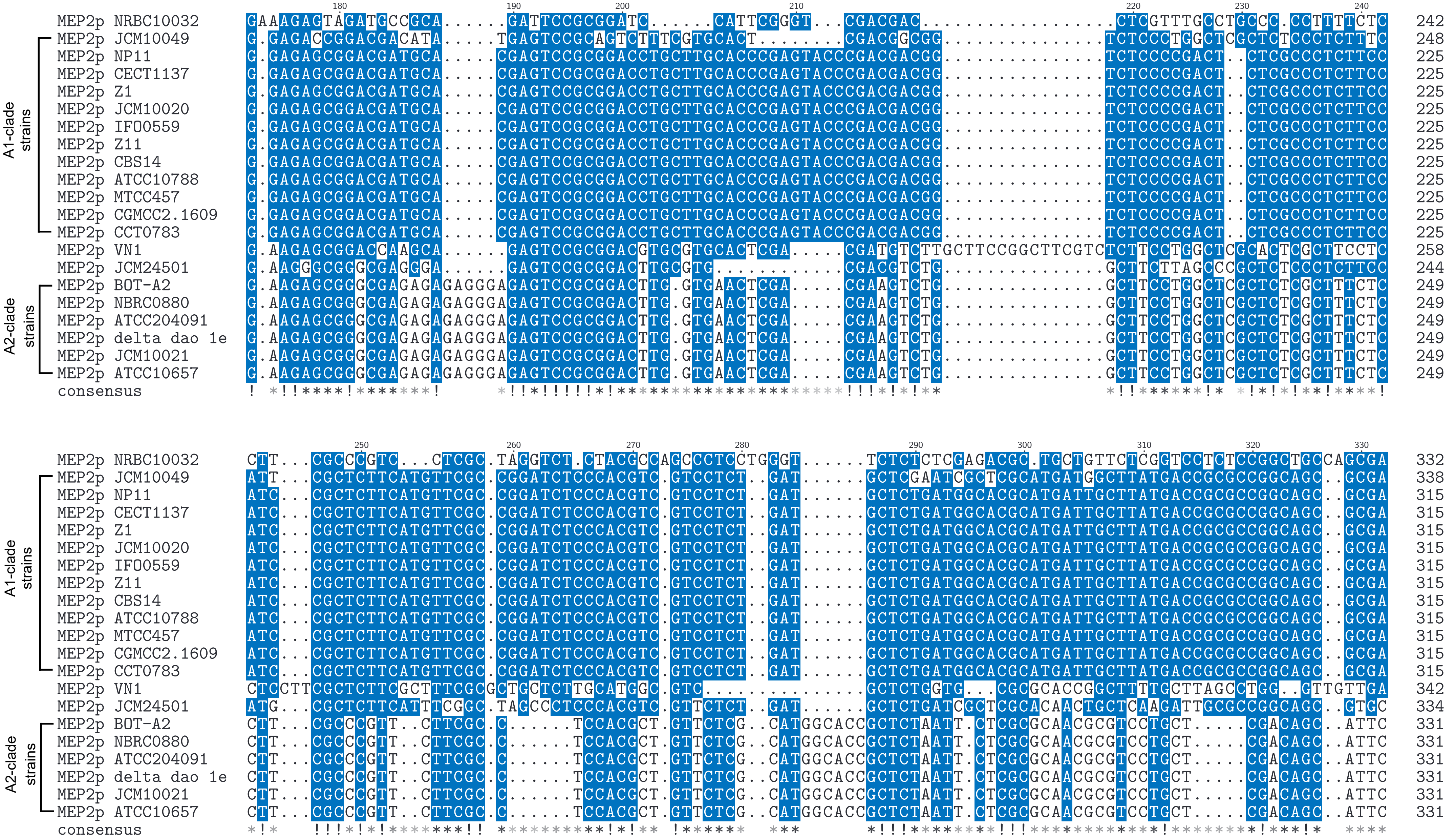

180
190
200
210
220
230
240
A1-clade strains
A2-clade strains
MEP2p NRBC10032 242
MEP2p JCM10049 248
MEP2p NP11 225
MEP2p CECT1137 225
MEP2p Z1 225
MEP2p JCM10020 225
MEP2p IFO0559 225
MEP2p Z11 225
MEP2p CBS14 225
MEP2p ATCC10788 225
MEP2p MTCC457 225
MEP2p CGMCC2.1609 225
MEP2p CCT0783 225
MEP2p VN1 258
MEP2p JCM24501 244
MEP2p BOT-A2 249
MEP2p NBRC0880 249
MEP2p ATCC204091 249
MEP2p delta dao 1e 249
MEP2p JCM10021 249
MEP2p ATCC10657 249
consensus
250
260
270
280
290
300
310
320
330
MEP2p NRBC10032 332
MEP2p JCM10049 338
MEP2p NP11 315
MEP2p CECT1137 315
MEP2p Z1 315
MEP2p JCM10020 315
MEP2p IFO0559 315
MEP2p Z11 315
MEP2p CBS14 315
MEP2p ATCC10788 315
MEP2p MTCC457 315
MEP2p CGMCC2.1609 315
MEP2p CCT0783 315
MEP2p VN1 342
MEP2p JCM24501 334
MEP2p BOT-A2 331
MEP2p NBRC0880 331
MEP2p ATCC204091 331
MEP2p delta dao 1e 331
MEP2p JCM10021 331
MEP2p ATCC10657 331
consensus

```
                        340             350                  360                   370                 380                 390                  400                410                 420
MEP2p NRBC10032       GACATCGTCAGAGGCGGATCCTTTC........CAGGACG...AGTGGGCGGAGTCGGCGGTAGAGGTGGTT...CGAGGGGCGCCATTTCGCTTTCCCACCGA   423
MEP2p JCM10049        .ACGGC..........GATTCAACC...T...GCGGAGCG...GAGGGTGGAGAGGAAGGAG.AG.GGTGATT...CGACGGGCGCCG.CTCGCACTGGTGCCGA   417
MEP2p NP11            .ACGGC..........GATTCATCC...T...GCGGAGCG...GAGGGAGCAAAGGAAGGAG.AG.GGTGATT...CGAGCGGGACCG.CTCGCATGCGTGCCGA   394
MEP2p CECT1137        .ACGGC..........GATTCATCC...T...GCGGAGCG...GAGGGAGCAAAGGAAGGAG.AG.GGTGATT...CGAGCGGGACCG.CTCGCATGCGTGCCGA   394
MEP2p Z1              .ACGGC..........GATTCATCC...T...GCGGAGCG...GAGGGAGCAAAGGAAGGAG.AG.GGTGATT...CGAGCGGGACCG.CTCGCATGCGTGCCGA   394
MEP2p JCM10020        .ACGGC..........GATTCATCC...T...GCGGAGCG...GAGGGAGCAAAGGAAGGAG.AG.GGTGATT...CGAGCGGGACCG.CTCGCATGCGTGCCGA   394
MEP2p IFO0559         .ACGGC..........GATTCATCC...T...GCGGAGCG...GAGGGAGCAAAGGAAGGAG.AG.GGTGATT...CGAGCGGGACCG.CTCGCATGCGTGCCGA   394
MEP2p Z11             .ACGGC..........GATTCATCC...T...GCGGAGCG...GAGGGAGCAAAGGAAGGAG.AG.GGTGATT...CGAGCGGGACCG.CTCGCATGCGTGCCGA   394
MEP2p CBS14           .ACGGC..........GATTCATCC...T...GCGGAGCG...GAGGGAGCAAAGGAAGGAG.AG.GGTGATT...CGAGCGGGACCG.CTCGCATGCGTGCCGA   394
MEP2p ATCC10788       .ACGGC..........GATTCATCC...T...GCGGAGCG...GAGGGAGCAAAGGAAGGAG.AG.GGTGATT...CGAGCGGGACCG.CTCGCATGCGTGCCGA   394
MEP2p MTCC457         .ACGGC..........GATTCATCC...T...GCGGAGCG...GAGGGAGCAAAGGAAGGAG.AG.GGTGATT...CGAGCGGGACCG.CTCGCATGCGTGCCGA   394
MEP2p CGMCC2.1609     .ACGGC..........GATTCATCC...T...GCGGAGCG...GAGGGAGCAAAGGAAGGAG.AG.GGTGATT...CGAGCGGGACCG.CTCGCATGCGTGCCGA   394
MEP2p CCT0783         .ACGGC..........GATTCATCC...T...GCGGAGCG...GAGGGAGCAAAGGAAGGAG.AG.GGTGATT...CGAGCGGGACCG.CTCGCATGCGTGCCGA   394
MEP2p VN1             .ACGCG..........GTTTCCATCGTCC...GCAGAGCA...........GAGGGAGGAG....GGTGGTT...CGAG.GGCGCCG.CTCGTACTCGTGGCGA   412
MEP2p JCM24501        .ACGGC..........GAGTCACTCGTCTACAGCAGAGCGGTAGAGGGGACAGAGGAA........GGTGGTT...CGAGGGGCGCCG.CTCGCGCTCGTGCCGA   416
MEP2p BOT-A2          GGAGGC..........GATACAAGCGGAG...GCAG.GCG...AGGGAG..GAGCAAGGAGGAG.AATCGTTCGACGAGGGGCGCCG.CTCGCACTCGTGCCGA   415
MEP2p NBRC0880        GGAGGC..........GATACAAGCGGAG...GCAG.GCG...AGGGAG..GAGCAAGGAGGAG.AATCGTTCGACGAGGGGCGCCG.CTCGCACTCGTGCCGA   415
MEP2p ATCC204091      GGAGGC..........GATACAAGCGGAG...GCAG.GCG...AGGGAG..GAGCAAGGAGGAG.AATCGTTCGACGAGGGGCGCCG.CTCGCACTCGTGCCGA   415
MEP2p delta dao 1e   GGAGGC..........GATACAAGCGGAG...GCAG.GCG...AGGGAG..GAGCAAGGAGGAG.AATCGTTCGACGAGGGGCGCCG.CTCGCACTCGTGCCGA   415
MEP2p JCM10021        GGAGGC..........GATACAAGCGGAG...GCAG.GCG...AGGGAG..GAGCAAGGAGGAG.AATCGTTCGACGAGGGGCGCCG.CTCGCACTCGTGCCGA   415
MEP2p ATCC10657       GGAGGC..........GATACAAGCGGAG...GCAG.GCG...AGGGAG..GAGCAAGGAGGAG.AATCGTTCGACGAGGGGCGCCG.CTCGCACTCGTGCCGA   415
consensus              *****          !**!*!***!    *   *!*!**!*   ***********!!*******  **  **!**!!   !!!**!!**!!*  *!!!**********!!!
```

A1-clade strains: JCM10049 – CCT0783
A2-clade strains: BOT-A2 – ATCC10657

```
                          430             440                450                460                470                480                                  490                500
MEP2p NRBC10032       TCCGGGCGAACCATCGACGG...GCACGAGCAGTGTCGTGCGGATCGCAGGGTCTTGCG...........ACGAAACGAGGGACGATCCTCCCG......CTCT   508
MEP2p JCM10049        TCCGGGCGAATGCGCGACCGATTGTGCAATTGCTGTCGCACA...TCGCAGGGCC.CGCAGAAGGAGGAGCGCGCCAG............CACGACGCGGTTTC   505
MEP2p NP11            TCCGGGCGAATGCGCGACCGATTGTACAATTGAGGGCGTTCG...TTTCAGGGCC.CGCAGAAGGAGAATCACGGCAGGACGGAAGAGCTTCTCAGGATGTTTTC   495
MEP2p CECT1137        TCCGGGCGAATGCGCGACCGATTGTACAATTGAGGGCGTTCG...TTTCAGGGCC.CGCAGAAGGAGAATCACGGCAGGACGGAAGAGCTTCTCAGGATGTTTTC   495
MEP2p Z1              TCCGGGCGAATGCGCGACCGATTGTACAATTGAGGGCGTTCG...TTTCAGGGCC.CGCAGAAGGAGAATCACGGCAGGACGGAAGAGCTTCTCAGGATGTTTTC   495
MEP2p JCM10020        TCCGGGCGAATGCGCGACCGATTGTACAATTGAGGGCGTTCG...TTTCAGGGCC.CGCAGAAGGAGAATCACGGCAGGACGGAAGAGCTTCTCAGGATGTTTTC   495
MEP2p IFO0559         TCCGGGCGAATGCGCGACCGATTGTACAATTGAGGGCGTTCG...TTTCAGGGCC.CGCAGAAGGAGAATCACGGCAGGACGGAAGAGCTTCTCAGGATGTTTTC   495
MEP2p Z11             TCCGGGCGAATGCGCGACCGATTGTACAATTGAGGGCGTTCG...TTTCAGGGCC.CGCAGAAGGAGAATCACGGCAGGACGGAAGAGCTTCTCAGGATGTTTTC   495
MEP2p CBS14           TCCGGGCGAATGCGCGACCGATTGTACAATTGAGGGCGTTCG...TTTCAGGGCC.CGCAGAAGGAGAATCACGGCAGGACGGAAGAGCTTCTCAGGATGTTTTC   495
MEP2p ATCC10788       TCCGGGCGAATGCGCGACCGATTGTACAATTGAGGGCGTTCG...TTTCAGGGCC.CGCAGAAGGAGAATCACGGCAGGACGGAAGAGCTTCTCAGGATGTTTTC   495
MEP2p MTCC457         TCCGGGCGAATGCGCGACCGATTGTACAATTGAGGGCGTTCG...TTTCAGGGCC.CGCAGAAGGAGAATCACGGCAGGACGGAAGAGCTTCTCAGGATGTTTTC   495
MEP2p CGMCC2.1609     TCCGGGCGAATGCGCGACCGATTGTACAATTGAGGGCGTTCG...TTTCAGGGCC.CGCAGAAGGAGAATCACGGCAGGACGGAAGAGCTTCTCAGGATGTTTTC   495
MEP2p CCT0783         TCCGGGCGAATGCGCGACCGATTGTACAATTGAGGGCGTTCG...TTTCAGGGCC.CGCAAAAGGAGAATCACGGCAGGACGGAAGAGCTTCTCAGGATGTTTTC   495
MEP2p VN1             TCCGGGCGAATGGGCGACCGACTGGACAATTGCTGGCGAGCA...TCGCAGGGTCACGCCGAAGGGAATCGACGGCA....GGAAGAGCTTCTCACGACGGTGTC   510
MEP2p JCM24501        TCCGGGCGAATGGGCGACCGACTGGACAATTGCCGGCGAGCA...TCGCAGGGCCACGCCGAAGGAAATCGAGGGCA....GGAAGAGCTGCTCACGACGGTTTC   514
MEP2p BOT-A2          TCCGGGCGAATGGGCGACCGACTGGACAATTGCCGGCGAGCA...TCGCAGGGTCACACCGAAGGGAATCGGGGGCA....GGAAGAGCTCCTCGAGACGGTTTC   513
MEP2p NBRC0880        TCCGGGCGAATGGGCGACCGACTGGACAATTGCCGGCGAGCA...TCGCAGGGTCACACCGAAGGGAATCGGGGGCA....GGAAGAGCTCCTCGAGACGGTTTC   513
MEP2p ATCC204091      TCCGGGCGAATGGGCGACCGACTGGACAATTGCCGGCGAGCA...TCGCAGGGTCACACCGAAGGGAATCGGGGGCA....GGAAGAGCTCCTCGAGACGGTTTC   513
MEP2p delta dao 1e   TCCGGGCGAATGGGCGACCGACTGGACAATTGCCGGCGAGCA...TCGCAGGGTCACACCGAAGGGAATCGGGGGCA....GGAAGAGCTCCTCGAGACGGTTTC   513
MEP2p JCM10021        TCCGGGCGAATGGGCGACCGACTGGACAATTGCCGGCGAGCA...TCGCAGGGTCACACCGAAGGGAATCGGGGGCA....GGAAGAGCTCCTCGAGACGGTTTC   513
MEP2p ATCC10657       TCCGGGCGAATGGGCGACCGACTGGACAATTGCCGGCGAGCA...TCGCAGGGTCACACCGAAGGGAATCGGGGGCA....GGAAGAGCTCCTCGAGACGGTTTC   513
consensus             !!!!!!!!!!***!!!!*!***!**!*!*****!*!!***!*   !**!!!!!*!  **!*************!**!****************!*!**********
```

A1-clade strains: JCM10049 – CCT0783
A2-clade strains: BOT-A2 – ATCC10657

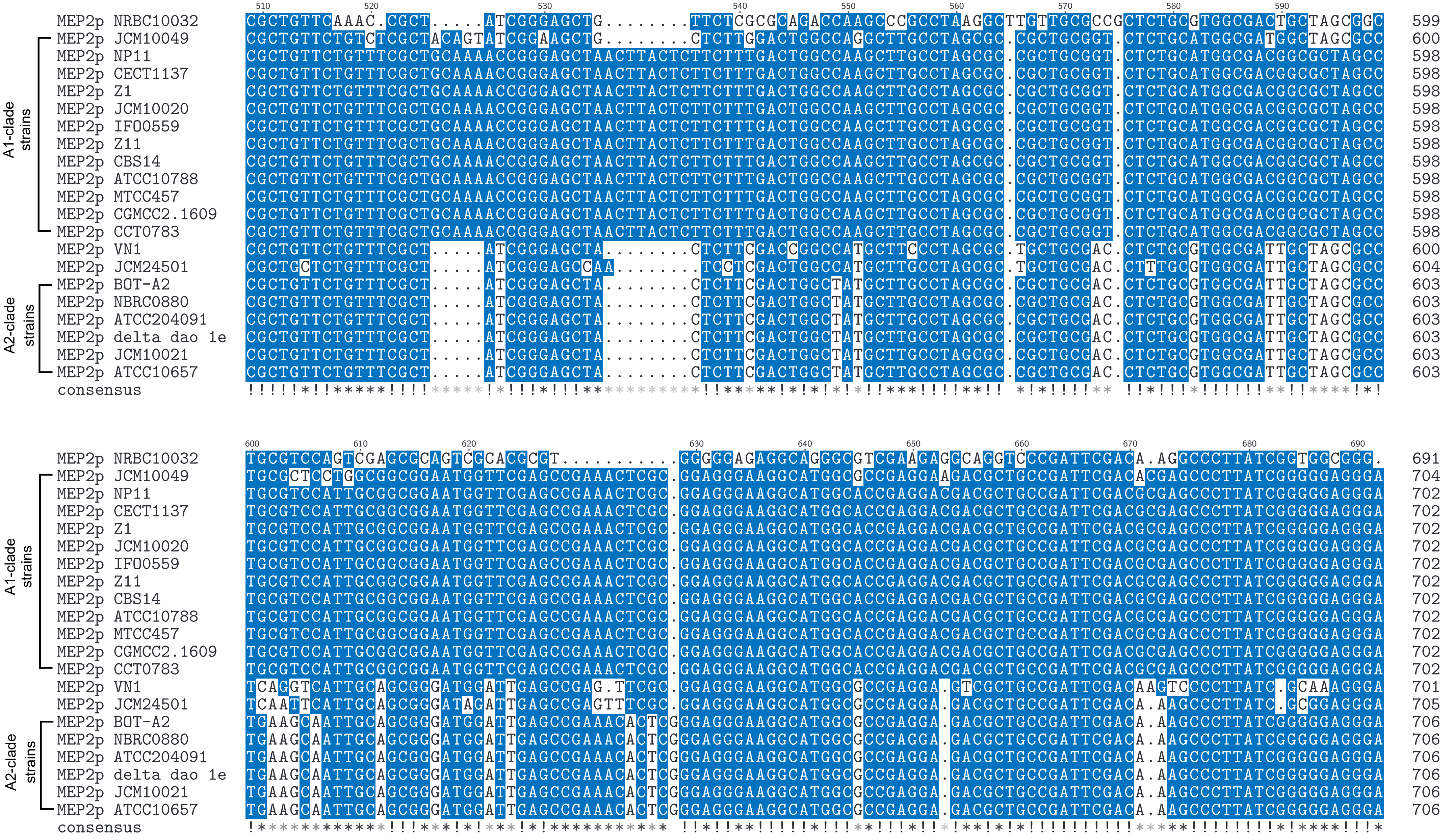

510 520 530 540 550 560 570 580 590
MEP2p NRBC10032 599
MEP2p JCM10049 600
MEP2p NP11 598
MEP2p CECT1137 598
MEP2p Z1 598
MEP2p JCM10020 598
MEP2p IFO0559 598
MEP2p Z11 598
MEP2p CBS14 598
MEP2p ATCC10788 598
MEP2p MTCC457 598
MEP2p CGMCC2.1609 598
MEP2p CCT0783 598
MEP2p VN1 600
MEP2p JCM24501 604
MEP2p BOT-A2 603
MEP2p NBRC0880 603
MEP2p ATCC204091 603
MEP2p delta dao 1e 603
MEP2p JCM10021 603
MEP2p ATCC10657 603
consensus
A1-clade strains
A2-clade strains
600 610 620 630 640 650 660 670 680 690
MEP2p NRBC10032 691
MEP2p JCM10049 704
MEP2p NP11 702
MEP2p CECT1137 702
MEP2p Z1 702
MEP2p JCM10020 702
MEP2p IFO0559 702
MEP2p Z11 702
MEP2p CBS14 702
MEP2p ATCC10788 702
MEP2p MTCC457 702
MEP2p CGMCC2.1609 702
MEP2p CCT0783 702
MEP2p VN1 701
MEP2p JCM24501 705
MEP2p BOT-A2 706
MEP2p NBRC0880 706
MEP2p ATCC204091 706
MEP2p delta dao 1e 706
MEP2p JCM10021 706
MEP2p ATCC10657 706
consensus
A1-clade strains
A2-clade strains

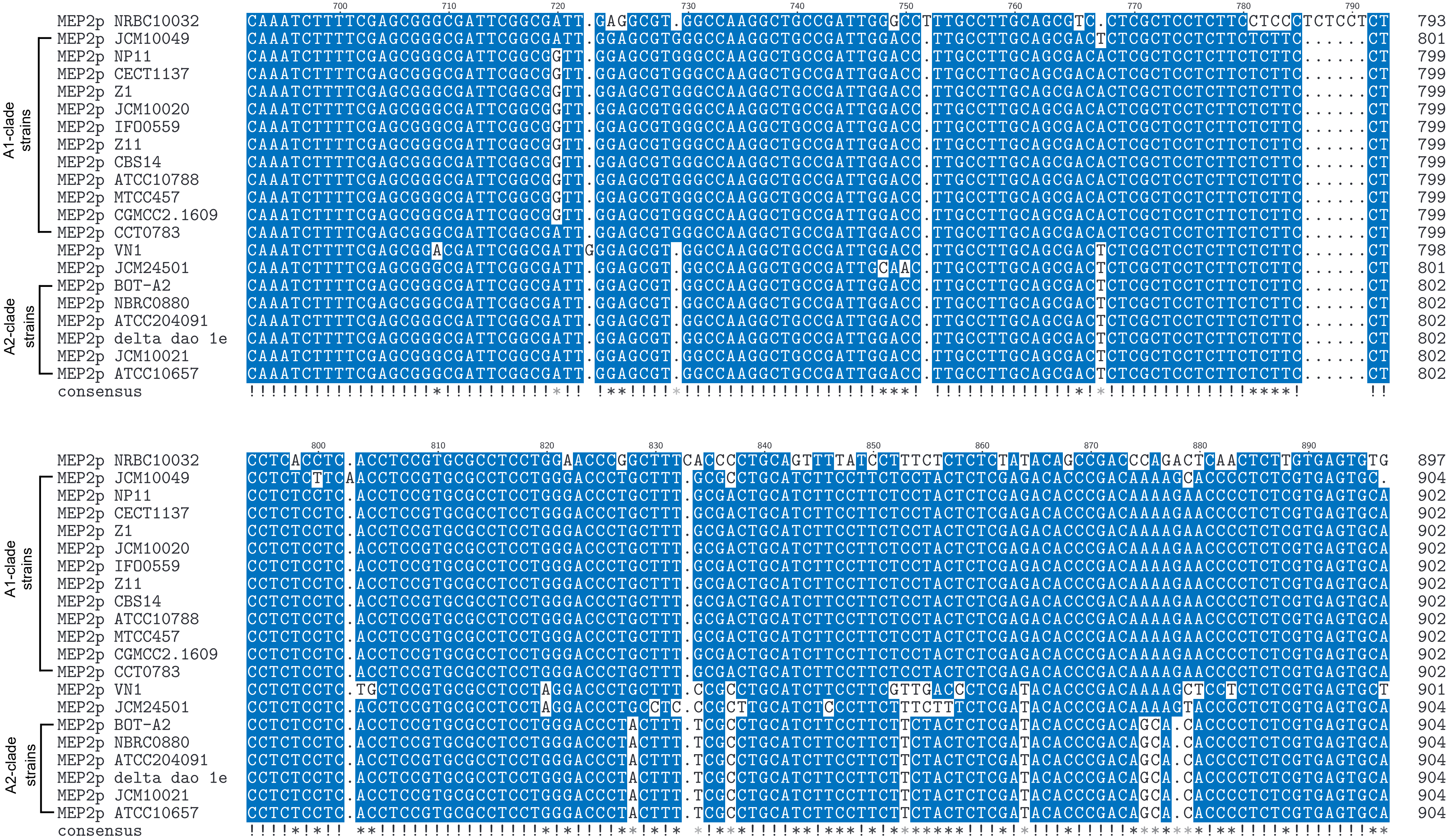
700 710 720 730 740 750 760 770 780 790
MEP2p NRBC10032 793
MEP2p JCM10049 801
MEP2p NP11 799
MEP2p CECT1137 799
MEP2p Z1 799
MEP2p JCM10020 799
MEP2p IFO0559 799
MEP2p Z11 799
MEP2p CBS14 799
MEP2p ATCC10788 799
MEP2p MTCC457 799
MEP2p CGMCC2.1609 799
MEP2p CCT0783 799
MEP2p VN1 798
MEP2p JCM24501 801
MEP2p BOT-A2 802
MEP2p NBRC0880 802
MEP2p ATCC204091 802
MEP2p delta dao 1e 802
MEP2p JCM10021 802
MEP2p ATCC10657 802
consensus
A1-clade strains
A2-clade strains
800 810 820 830 840 850 860 870 880 890
MEP2p NRBC10032 897
MEP2p JCM10049 904
MEP2p NP11 902
MEP2p CECT1137 902
MEP2p Z1 902
MEP2p JCM10020 902
MEP2p IFO0559 902
MEP2p Z11 902
MEP2p CBS14 902
MEP2p ATCC10788 902
MEP2p MTCC457 902
MEP2p CGMCC2.1609 902
MEP2p CCT0783 902
MEP2p VN1 901
MEP2p JCM24501 904
MEP2p BOT-A2 904
MEP2p NBRC0880 904
MEP2p ATCC204091 904
MEP2p delta dao 1e 904
MEP2p JCM10021 904
MEP2p ATCC10657 904
consensus
A1-clade strains
A2-clade strains

```
                          900       910       920       930       940       950       960       970       980       990       1000
                       MEP2p NRBC10032      GTTCACCAGGATC.GTCTCACCCTTCGAGGGGCGCTCCCTTATCTTCCACTGACAATCCGCTTCTCTCCCCCTCTCGCTCTTCGCTCAGCAGTGCGACTCGGCC  1000
A1-clade strains   [ MEP2p JCM10049       ..TCGCCTCGCCC.GCCTTCCCCTTCGAGGCGCGCACCCTTATCTTCCGCTAACAACCCGCTTCCCTCC....CTCGCTCCTCGCTC.GCAGTGCGGCTAAACC  1000
                   | MEP2p NP11           GCTCGCCTCGCCC.GCCTTCCCCTTCGAGGCGCGCGCCCTTATCTTCCGCTAACAACCCGCTTCTCTCC....CTCGCTCCTCGCTC.GCAGTGCGGCTAAACC  1000
                   | MEP2p CECT1137       GCTCGCCTCGCCC.GCCTTCCCCTTCGAGGCGCGCGCCCTTATCTTCCGCTAACAACCCGCTTCTCTCC....CTCGCTCCTCGCTC.GCAGTGCGGCTAAACC  1000
                   | MEP2p Z1             GCTCGCCTCGCCC.GCCTTCCCCTTCGAGGCGCGCGCCCTTATCTTCCGCTAACAACCCGCTTCTCTCC....CTCGCTCCTCGCTC.GCAGTGCGGCTAAACC  1000
                   | MEP2p JCM10020       GCTCGCCTCGCCC.GCCTTCCCCTTCGAGGCGCGCGCCCTTATCTTCCGCTAACAACCCGCTTCTCTCC....CTCGCTCCTCGCTC.GCAGTGCGGCTAAACC  1000
                   | MEP2p IFO0559        GCTCGCCTCGCCC.GCCTTCCCCTTCGAGGCGCGCGCCCTTATCTTCCGCTAACAACCCGCTTCTCTCC....CTCGCTCCTCGCTC.GCAGTGCGGCTAAACC  1000
                   | MEP2p Z11            GCTCGCCTCGCCC.GCCTTCCCCTTCGAGGCGCGCGCCCTTATCTTCCGCTAACAACCCGCTTCTCTCC....CTCGCTCCTCGCTC.GCAGTGCGGCTAAACC  1000
                   | MEP2p CBS14          GCTCGCCTCGCCC.GCCTTCCCCTTCGAGGCGCGCGCCCTTATCTTCCGCTAACAACCCGCTTCTCTCC....CTCGCTCCTCGCTC.GCAGTGCGGCTAAACC  1000
                   | MEP2p ATCC10788      GCTCGCCTCGCCC.GCCTTCCCCTTCGAGGCGCGCGCCCTTATCTTCCGCTAACAACCCGCTTCTCTCC....CTCGCTCCTCGCTC.GCAGTGCGGCTAAACC  1000
                   | MEP2p MTCC457        GCTCGCCTCGCCC.GCCTTCCCCTTCGAGGCGCGCGCCCTTATCTTCCGCTAACAACCCGCTTCTCTCC....CTCGCTCCTCGCTC.GCAGTGCGGCTAAACC  1000
                   | MEP2p CGMCC2.1609    GCTCGCCTCGCCC.GCCTTCCCCTTCGAGGCGCGCGCCCTTATCTTCCGCTAACAACCCGCTTCTCTCC....CTCGCTCCTCGCTC.GCAGTGCGGCTAAACC  1000
                   [ MEP2p CCT0783        GCTCGCCTCGCCC.GCCTTCCCCTTCGAGGCGCGCGCCCTTATCTTCCGCTAACAACCCGCTTCTCTCC....CTCGCTCCTCGCTC.GCAGTGCGGCTAAACC  1000
                     MEP2p VN1            GCTCGCCTCGCTCTCCCTTCCCCTTCGAGGCGCGCACCCTTATCTTCCGCTAACAACCCGCTTCTCTCC....CTCGCTCCTCCCTC.GCAGTGCGGCTGAACC  1000
                     MEP2p JCM24501       CTTCGCCTCG.CC.CCCTTTCCCTTCGAGGCGCGCG.CCTTATCTTCGGCTAACAACGCGCTTCTATCC....CTCGCTCGTCGCTC.GCAGTGCTGCTGAACA  1000
A2-clade strains   [ MEP2p BOT-A2         GTTCGCCTCG.CC.CCCTTCCCCTTCGAGGCGCGCG.CCTTATCTTCCGCTAACAACCCGCTTCTCTCC....CTCGCTTCCCTCTC.GCAGTGCGGCTGAACC  1000
                   | MEP2p NBRC0880       GTTCGCCTCG.CC.CCCTTCCCCTTCGAGGCGCGCG.CCTTATCTTCCGCTAACAACCCGCTTCTCTCC....CTCGCTTCCCTCTC.GCAGTGCGGCTGAACC  1000
                   | MEP2p ATCC204091     GTTCGCCTCG.CC.CCCTTCCCCTTCGAGGCGCGCG.CCTTATCTTCCGCTAACAACCCGCTTCTCTCC....CTCGCTTCCCTCTC.GCAGTGCGGCTGAACC  1000
                   | MEP2p delta dao 1e   GTTCGCCTCG.CC.CCCTTCCCCTTCGAGGCGCGCG.CCTTATCTTCCGCTAACAACCCGCTTCTCTCC....CTCGCTTCCCTCTC.GCAGTGCGGCTGAACC  1000
                   | MEP2p JCM10021       GTTCGCCTCG.CC.CCCTTCCCCTTCGAGGCGCGCG.CCTTATCTTCCGCTAACAACCCGCTTCTCTCC....CTCGCTTCCCTCTC.GCAGTGCGGCTGAACC  1000
                   [ MEP2p ATCC10657      GTTCGCCTCG.CC.CCCTTCCCCTTCGAGGCGCGCG.CCTTATCTTCCGCTAACAACCCGCTTCTCTCC....CTCGCTTCCCTCTC.GCAGTGCGGCTGAACC  1000
                     consensus            **!!*!!**!**! **!!**!!!!!!!!!!*!!!!**!!!!!!!!!!**!!*!!!!**!!!!!!**!!!    !!!!!!***!*!!! !!!!!!!**!!***!*
```
