# Supplementary material for: Expanding the genetic toolbox of Rhodotorula toruloides by identification and validation of six novel promoters induced or repressed under nitrogen starvation
Source: Microb Cell Fact. 2023 Aug 19;22:160. doi: 10.1186/s12934-023-02175-2 (PMC10440040; doi:10.1186/s12934-023-02175-2)
Supplement: Supplementary file 5 — Additional file 5. Multiple sequence alignment results are for the 3877 promoter. [file 12934_2023_2175_MOESM5_ESM.pdf]

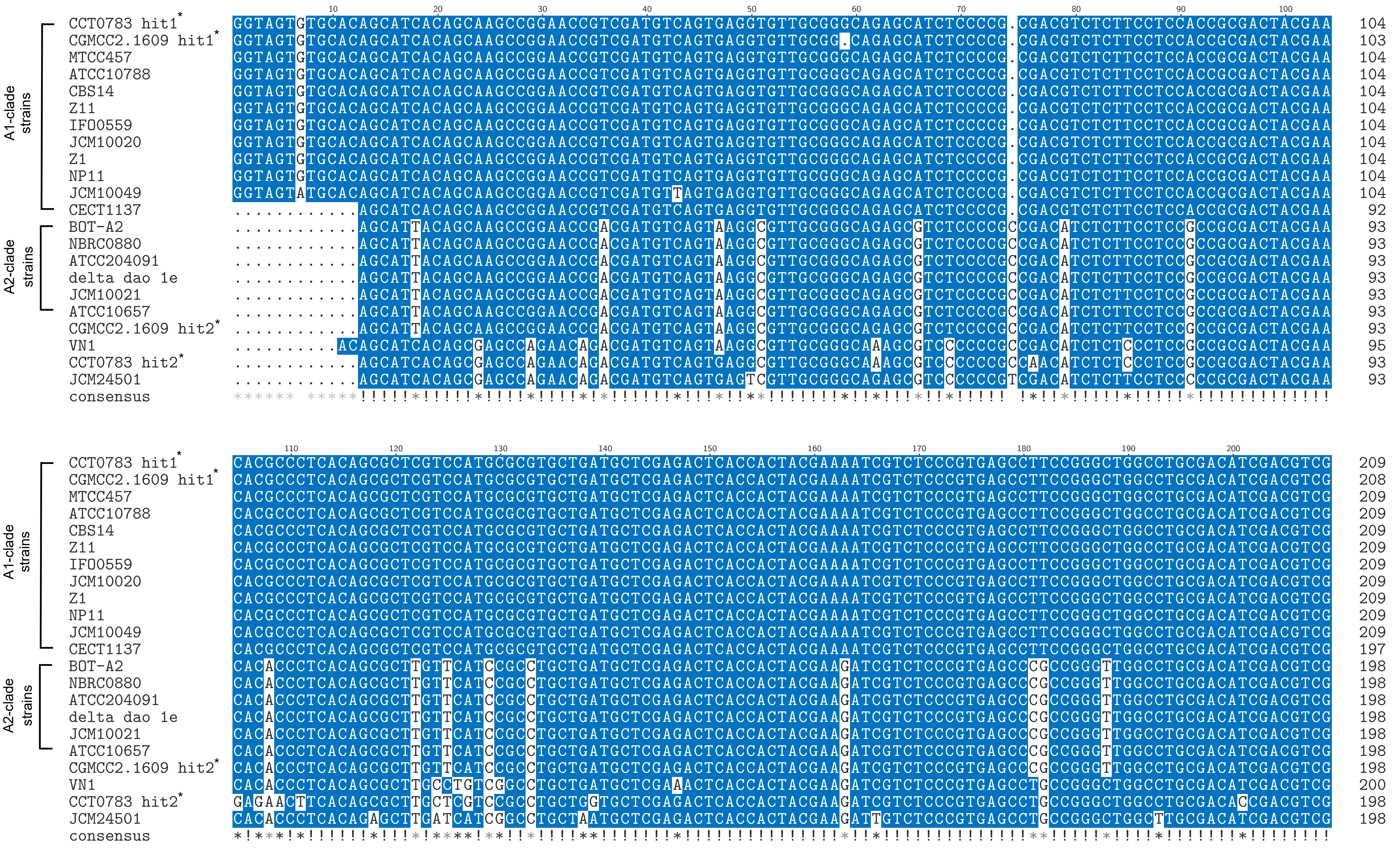

Multiple Sequence Alignment (MSA) of the putative 3877 promoter sequences from all the currently available *R. toruloides* genomes. The candidate sequence from strain BOT-A2 was identified as the 1000 kb directly upstream of the start codon of the gene, and this sequence was used as input for a nucleotide Blast analysis to identify the corresponding sequences in the other genomes. No Blast hits were found in the NRBC10032 assembly; two hits were found in each of the CGMCC2.1609 and CCT0783 assemblies and are marked with *. See the Materials and Methods section for details on the MSA. The brackets indicate the established clades from the Average Nucleotide Identity analysis (see Figure 1 in the main text).

```
                          210       220       230       240       250       260       270       280       290        300       310
   CCT0783 hit1*          AGGCGACAGGGACCATGTCGACAGAAGGGCGTTTGCGGGTGTCGTAGACGGTGGAGAGGCCTTGTACAGATGCGCAGGGTCTTGAAG.AAGTGCTCTTGTCGAGG  313
   CGMCC2.1609 hit1*      AGGCGACAGGGACCATGTCGACAGAAGGGCGTTTGCGGGTGTCGTAGACGGTGGAGAGGCCTTGTACAGATGCGCAGGGTCTTGAAG.AAGTGCTCTTGTCGAGG  312
   MTCC457                AGGCGACAGGGACCATGTCGACAGAAGGGCGTTTGCGGGTGTCGTAGACGGTGGAGAGGCCTTGTACAGATGCGCAGGGTCTTGAAG.AAGTGCTCTTGTCGAGG  313
   ATCC10788              AGGCGACAGGGACCATGTCGACAGAAGGGCGTTTGCGGGTGTCGTAGACGGTGGAGAGGCCTTGTACAGATGCGCAGGGTCTTGAAG.AAGTGCTCTTGTCGAGG  313
   CBS14                  AGGCGACAGGGACCATGTCGACAGAAGGGCGTTTGCGGGTGTCGTAGACGGTGGAGAGGCCTTGTACAGATGCGCAGGGTCTTGAAG.AAGTGCTCTTGTCGAGG  313
A1-clade strains
   Z11                    AGGCGACAGGGACCATGTCGACAGAAGGGCGTTTGCGGGTGTCGTAGACGGTGGAGAGGCCTTGTACAGATGCGCAGGGTCTTGAAG.AAGTGCTCTTGTCGAGG  313
   IFO0559                AGGCGACAGGGACCATGTCGACAGAAGGGCGTTTGCGGGTGTCGTAGACGGTGGAGAGGCCTTGTACAGATGCGCAGGGTCTTGAAG.AAGTGCTCTTGTCGAGG  313
   JCM10020               AGGCGACAGGGACCATGTCGACAGAAGGGCGTTTGCGGGTGTCGTAGACGGTGGAGAGGCCTTGTACAGATGCGCAGGGTCTTGAAG.AAGTGCTCTTGTCGAGG  313
   Z1                     AGGCGACAGGGACCATGTCGACAGAAGGGCGTTTGCGGGTGTCGTAGACGGTGGAGAGGCCTTGTACAGATGCGCAGGGTCTTGAAG.AAGTGCTCTTGTCGAGG  313
   NP11                   AGGCGACAGGGACCATGTCGACAGAAGGGCGTTTGCGGGTGTCGTAGACGGTGGAGAGGCCTTGTACAGATGCGCAGGGTCTTGAAG.AAGTGCTCTTGTCGAGG  313
   JCM10049               AGGCGACAGGGACCATGTCGACAGAAGGGCGTTTGCGGGTGTCGTAGACGGTGGAGAGGCCTTGTACAGATGCGCAGGGTCTTGAAG.AAGTGCTCTTGTCGAGG  313
   CECT1137               AGGCGACAGGGACCATGTCGACAGAAGGGCGTTTGCGGGTGTCGTAGACGGTGGAGAGGCCTTGTACAGATGCGCAGGGTCTTGAAG.AAGTGCTCTTGTCGAGG  301
   BOT-A2                 AGGCGACAGGGACCATGTCGAGGGAAACGCGGTCGCGGGTTTCGTGAAGGTTGGGGAGGTTGGCTGGTGATGTGCAGGGACTCTGAGGAAATGCTCTGGGCGAGC  303
   NBRC0880               AGGCGACAGGGACCATGTCGAGGGAAACGCGGTCGCGGGTTTCGTGAAGGTTGGGGAGGTTGGCTGGTGATGTGCAGGGACTCTGAGGAAATGCTCTGGGCGAGC  303
A2-clade strains
   ATCC204091             AGGCGACAGGGACCATGTCGAGGGAAACGCGGTCGCGGGTTTCGTGAAGGTTGGGGAGGTTGGCTGGTGATGTGCAGGGACTCTGAGGAAATGCTCTGGGCGAGC  303
   delta dao 1e           AGGCGACAGGGACCATGTCGAGGGAAACGCGGTCGCGGGTTTCGTGAAGGTTGGGGAGGTTGGCTGGTGATGTGCAGGGACTCTGAGGAAATGCTCTGGGCGAGC  303
   JCM10021               AGGCGACAGGGACCATGTCGAGGGAAACGCGGTCGCGGGTTTCGTGAAGGTTGGGGAGGTTGGCTGGTGATGTGCAGGGACTCTGAGGAAATGCTCTGGGCGAGC  303
   ATCC10657              AGGCGACAGGGACCATGTCGAGGGAAACGCGGTCGCGGGTTTCGTGAAGGTTGGGGAGGTTGGCTGGTGATGTGCAGGGACTCTGAGGAAATGCTCTGGGCGAGC  303
   CGMCC2.1609 hit2*      AGGCGACAGGGACCATGTCGAGGGAAACGCGGTCGCGGGTTTCGTGAAGGTTGGGGAGGTTGGCTGGTGATGTGCAGGGACTCTGAGGAAATGCTCTGGGCGAGC  303
   VN1                    AGGCGACAGGGACCATGTCGAGGGAAGAGCGTGGCGGGCGTTCAAGAGGTTGGAGAAGTATGGCGGAGATGTGCAGGATCTTTAAC.AAGCGCTCTGGGCGAGC  304
   CCT0783 hit2*          AGGCGACAGGGACCATGTCGAGGGAAGAGCGTGCGCGGGCGTTCAAGAGGTTGGAGAAGTATGGCGGAGATGTGCAGGGTCTTTAAC.AAGCGCTCTGGGCGAGC  302
   JCM24501               AGGCGACAGGGACCATGTCGAGGGAAAGGCGGCTACGGGTTCGTGGAGGTTGGAGAGGTCTGGTGGAGATGTGGGAGGTGTTCGAG.GAGTGGTGTGGGCGAGC  302
   consensus              !!!!!!!!!!!!!!!!!!!!**!!!**!!!****!!!!**!*****!*!*!!!*!!*!!*!*********!!!!*!***!***!***!* *!**!*!*!*!*!!!!*

                          320       330       340       350       360       370       380       390       400       410
   CCT0783 hit1*          TGGAGTAGCAGCGAGGCAGCGCAGGCTCTGAAGGACCTGTTCTCGTCAACCTGCGTCAAGGATGGGCGCGGTGCGAAGGGGCGGCTTCGGATTGGCGCACGATTA  418
   CGMCC2.1609 hit1*      TGGAGTAGCAGCGAGGCAGCGCAGGCTCTGAAGGACCTGTTCTCGTCAACCTGCGTCAAGGATGGGCGCGGTGCGAAGGGGCGGCTTCGGATTGGCGCACGATTA  417
   MTCC457                TGGAGTAGCAGCGAGGCAGCGCAGGCTCTGAAGGACCTGTTCTCGTCAACCTGCGTCAAGGATGGGCGCGGTGCGAAGGGGCGGCTTCGGATTGGCGCACGATTA  418
   ATCC10788              TGGAGTAGCAGCGAGGCAGCGCAGGCTCTGAAGGACCTGTTCTCGTCAACCTGCGTCAAGGATGGGCGCGGTGCGAAGGGGCGGCTTCGGATTGGCGCACGATTA  418
   CBS14                  TGGAGTAGCAGCGAGGCAGCGCAGGCTCTGAAGGACCTGTTCTCGTCAACCTGCGTCAAGGATGGGCGCGGTGCGAAGGGGCGGCTTCGGATTGGCGCACGATTA  418
A1-clade strains
   Z11                    TGGAGTAGCAGCGAGGCAGCGCAGGCTCTGAAGGACCTGTTCTCGTCAACCTGCGTCAAGGATGGGCGCGGTGCGAAGGGGCGGCTTCGGATTGGCGCACGATTA  418
   IFO0559                TGGAGTAGCAGCGAGGCAGCGCAGGCTCTGAAGGACCTGTTCTCGTCAACCTGCGTCAAGGATGGGCGCGGTGCGAAGGGGCGGCTTCGGATTGGCGCACGATTA  418
   JCM10020               TGGAGTAGCAGCGAGGCAGCGCAGGCTCTGAAGGACCTGTTCTCGTCAACCTGCGTCAAGGATGGGCGCGGTGCGAAGGGGCGGCTTCGGATTGGCGCACGATTA  418
   Z1                     TGGAGTAGCAGCGAGGCAGCGCAGGCTCTGAAGGACCTGTTCTCGTCAACCTGCGTCAAGGATGGGCGCGGTGCGAAGGGGCGGCTTCGGATTGGCGCACGATTA  418
   NP11                   TGGAGTAGCAGCGAGGCAGCGCAGGCTCTGAAGGACCTGTTCTCGTCAACCTGCGTCAAGGATGGGCGCGGTGCGAAGGGGCGGCTTCGGATTGGCGCACGATTA  418
   JCM10049               TGGAGTAGCAGCGAGGCAGCGCAGGCTCTGAAGGACCTGTTCTCGTCAACCTGCGTCAAGGATGGGCGCGGTGCGAAGGGGCGGCTTCGGATTGGCGCACGATTA  418
   CECT1137               TGGAGTAGCAGCGAGGCAGCGCAGGCTCTGAAGGACCTGTTCTCGTCAACCTGCGTCAAGGATGGGCGCGGTGCGAAGGGGCGGCTTCGGATTGGCGCACGATTA  406
   BOT-A2                 GGGAGTAGCAGCGAGGCAGCACAGGCTCTGACGGAGCTGTTCTCGTCGACCTGCGTCGAGGATGGGCGCGGTGCAAAGGGGCGGCTTCGGATTGGCGCACGATTG  408
   NBRC0880               GGGAGTAGCAGCGAGGCAGCACAGGCTCTGACGGAGCTGTTCTCGTCGACCTGCGTCGAGGATGGGCGCGGTGCAAAGGGGCGGCTTCGGATTGGCGCACGATTG  408
A2-clade strains
   ATCC204091             GGGAGTAGCAGCGAGGCAGCACAGGCTCTGACGGAGCTGTTCTCGTCGACCTGCGTCGAGGATGGGCGCGGTGCAAAGGGGCGGCTTCGGATTGGCGCACGATTG  408
   delta dao 1e           GGGAGTAGCAGCGAGGCAGCACAGGCTCTGACGGAGCTGTTCTCGTCGACCTGCGTCGAGGATGGGCGCGGTGCAAAGGGGCGGCTTCGGATTGGCGCACGATTG  408
   JCM10021               GGGAGTAGCAGCGAGGCAGCACAGGCTCTGACGGAGCTGTTCTCGTCGACCTGCGTCGAGGATGGGCGCGGTGCAAAGGGGCGGCTTCGGATTGGCGCACGATTG  408
   ATCC10657              GGGAGTAGCAGCGAGGCAGCACAGGCTCTGACGGAGCTGTTCTCGTCGACCTGCGTCGAGGATGGGCGCGGTGCAAAGGGGCGGCTTCGGATTGGCGCACGATTG  408
   CGMCC2.1609 hit2*      GGGAGTAGCAGCGAGGCAGCACAGGCTCTGACGGAGCTGTTCTCGTCGACCTGCGTCGAGGATGGGCGCGGTGCAAAGGGGCGGCTTCGGATTGGCGCACGATTG  408
   VN1                    TGGAGTAGCAGCGAGGCAGCAGAGGCTCTGAGGGACCTGTTCTCGTCGACCTCTGTCGAGGATGGGCGCGGTGCGAAGGGGCGGCTTCGGATTGGCGCACGATTG  409
   CCT0783 hit2*          TGGAGTAGCAGCGAGGCAGCACAGGCTCTGGGGGACCTGTACTCGTTGACCTCTGTCGAGGATGGGCGCGGTGCGAAGGGGCGGCTTCGGATTGGCGCGCGATTG  407
   JCM24501               GGGATCAGCAGCGAGGCAGCACAGGCTCTGACGGAGCTGTTCTCGTCGACTTGCGTCGAGGATGGGCGCGGTGCGAAGGGGCGGCTTCGGATTGGCGCACGATTG  407
   consensus              *!!!**!!!!!!!!!!!!!!*!*!!!!!!!!*!!!*!!!!*!!!!!**!!*!**!!!*!!!!!!!!!!!!!!!!!*!!!!!!!!!!!!!!!!!!!!!!!!*!!!!!*
```

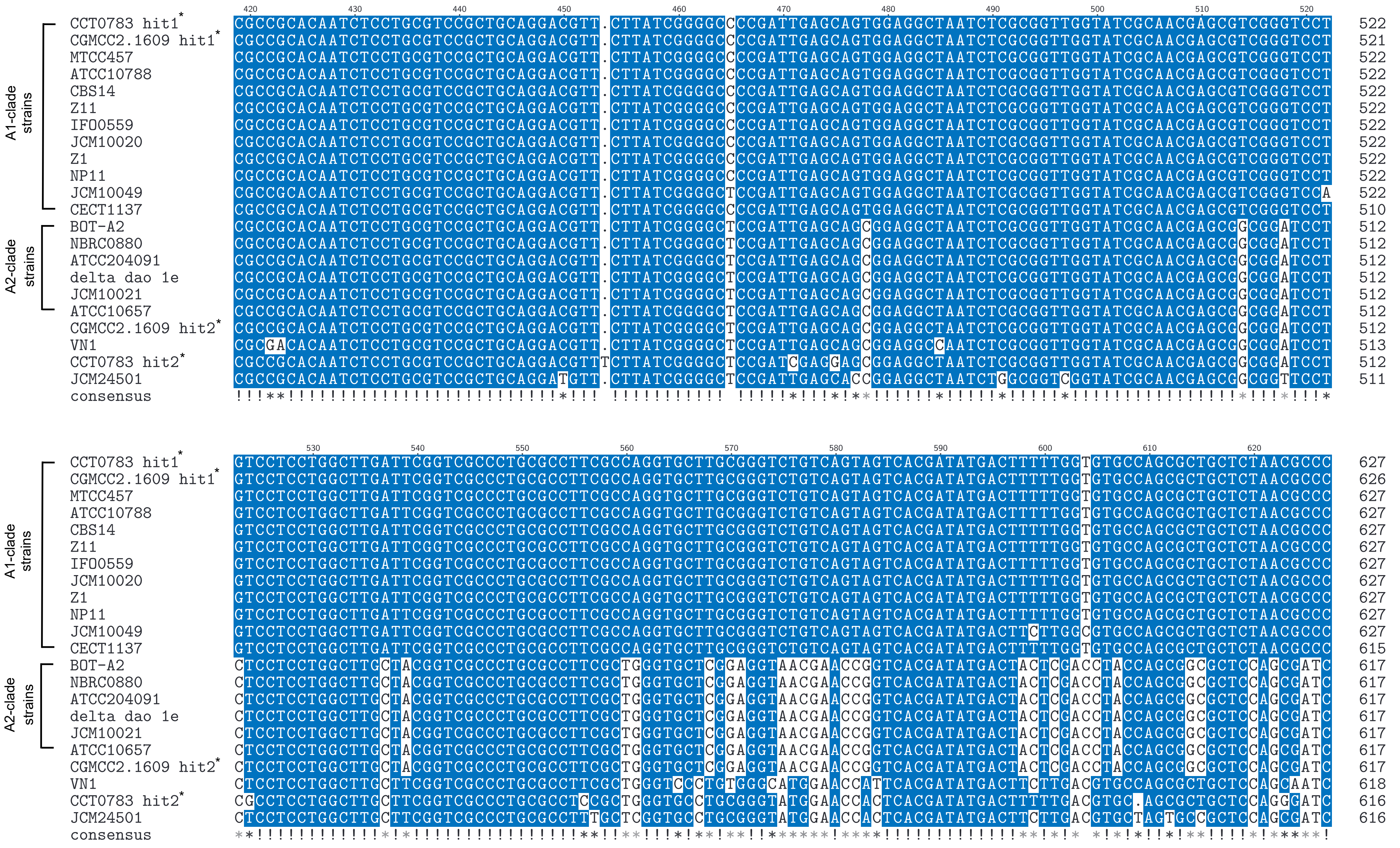

A1-clade strains
A2-clade strains
CCT0783 hit1*
CGMCC2.1609 hit1*
MTCC457
ATCC10788
CBS14
Z11
IFO0559
JCM10020
Z1
NP11
JCM10049
CECT1137
BOT-A2
NBRC0880
ATCC204091
delta dao 1e
JCM10021
ATCC10657
CGMCC2.1609 hit2*
VN1
CCT0783 hit2*
JCM24501
consensus
420
430
440
450
460
470
480
490
500
510
520
522
521
522
522
522
522
522
522
522
522
522
510
512
512
512
512
512
512
512
513
512
511
530
540
550
560
570
580
590
600
610
620
627
626
627
627
627
627
627
627
627
627
627
615
617
617
617
617
617
617
617
618
616
616

Clade	Strain	630 640 650 660 670 680 690 700 710 720 730	
A1-clade strains	CCT0783 hit1*	CCTCGTCACCAGCGAAGGCGGCAGCCGTTTCGCCCCGCCATTTCGTCGCGCGGCGCTCTTTCATTTTCCCCTCCCCGCACCGCTTCCCCTTCGACGGTGCCCTGC	732
	CGMCC2.1609 hit1*	CCTCGTCACCAGCGAAGGCGGCAGCCGTTTCGCCCCGCCATTTCGTCGCGCGGCGCTCTTTCATTTTCCCCTCCCCGCACCGCTTCCCCTTCGACGGTGCCCTGC	731
	MTCC457	CCTCGTCACCAGCGAAGGCGGCAGCCGTTTCGCCCCGCCATTTCGTCGCGCGGCGCTCTTTCATTTTCCCCTCCCCGCACCGCTTCCCCTTCGACGGTGCCCTGC	732
	ATCC10788	CCTCGTCACCAGCGAAGGCGGCAGCCGTTTCGCCCCGCCATTTCGTCGCGCGGCGCTCTTTCATTTTCCCCTCCCCGCACCGCTTCCCCTTCGACGGTGCCCTGC	732
	CBS14	CCTCGTCACCAGCGAAGGCGGCAGCCGTTTCGCCCCGCCATTTCGTCGCGCGGCGCTCTTTCATTTTCCCCTCCCCGCACCGCTTCCCCTTCGACGGTGCCCTGC	732
	Z11	CCTCGTCACCAGCGAAGGCGGCAGCCGTTTCGCCCCGCCATTTCGTCGCGCGGCGCTCTTTCATTTTCCCCTCCCCGCACCGCTTCCCCTTCGACGGTGCCCTGC	732
	IFO0559	CCTCGTCACCAGCGAAGGCGGCAGCCGTTTCGCCCCGCCATTTCGTCGCGCGGCGCTCTTTCATTTTCCCCTCCCCGCACCGCTTCCCCTTCGACGGTGCCCTGC	732
	JCM10020	CCTCGTCACCAGCGAAGGCGGCAGCCGTTTCGCCCCGCCATTTCGTCGCGCGGCGCTCTTTCATTTTCCCCTCCCCGCACCGCTTCCCCTTCGACGGTGCCCTGC	732
	Z1	CCTCGTCACCAGCGAAGGCGGCAGCCGTTTCGCCCCGCCATTTCGTCGCGCGGCGCTCTTTCATTTTCCCCTCCCCGCACCGCTTCCCCTTCGACGGTGCCCTGC	732
	NP11	CCTCGTCACCAGCGAAGGCGGCAGCCGTTTCGCCCCGCCATTTCGTCGCGCGGCGCTCTTTCATTTTCCCCTCCCCGCACCGCTTCCCCTTCGACGGTGCCCTGC	732
	JCM10049	CCTCGTCACCAGCGAAGGCGGCAGCCGTTTCGCCCCGCCATTTCGTCGCGCGGCGCTCTTTCATTTTCCCCTCCCCGCACCGCTTCCCCTTCGACGGTGCCCTGC	732
	CECT1137	CCTCGTCACCAGCGAAGGCGGCAGCCGTTTCGCCCCGCCATTTCGTCGCGCGGCGCTCTTTCATTTTCCCCTCCCCGCACCGCTTCCCCTTCGACGGTGCCCTGC	720
A2-clade strains	BOT-A2	TCCCAGGACGAGCGAAGGCGGCAGCCATTTCGCCCCGGCATTTCGTCGCGCGGCGCTCTTTCATTTTCCCCTCCCCGCACAGCTTCCCCTTCGACGACACCCTGC	722
	NBRC0880	TCCCAGGACGAGCGAAGGCGGCAGCCATTTCGCCCCGGCATTTCGTCGCGCGGCGCTCTTTCATTTTCCCCTCCCCGCACAGCTTCCCCTTCGACGACACCCTGC	722
	ATCC204091	TCCCAGGACGAGCGAAGGCGGCAGCCATTTCGCCCCGGCATTTCGTCGCGCGGCGCTCTTTCATTTTCCCCTCCCCGCACAGCTTCCCCTTCGACGACACCCTGC	722
	delta dao 1e	TCCCAGGACGAGCGAAGGCGGCAGCCATTTCGCCCCGGCATTTCGTCGCGCGGCGCTCTTTCATTTTCCCCTCCCCGCACAGCTTCCCCTTCGACGACACCCTGC	722
	JCM10021	TCCCAGGACGAGCGAAGGCGGCAGCCATTTCGCCCCGGCATTTCGTCGCGCGGCGCTCTTTCATTTTCCCCTCCCCGCACAGCTTCCCCTTCGACGACACCCTGC	722
	ATCC10657	TCCCAGGACGAGCGAAGGCGGCAGCCATTTCGCCCCGGCATTTCGTCGCGCGGCGCTCTTTCATTTTCCCCTCCCCGCACAGCTTCCCCTTCGACGACACCCTGC	722
	CGMCC2.1609 hit2*	TCCCAGGACGAGCGAAGGCGGCAGCCATTTCGCCCCGGCATTTCGTCGCGCGGCGCTCTTTCATTTTCCCCTCCCCGCACAGCTTCCCCTTCGACGACACCCTGC	722
	VN1	TCCCAGGATGAGCGAAGGCGGCAGCCATTTCGCCCCGCCATTTCGTCGCCCGGCGCTCTTTCATTTTCCCCTCCCCGCACCGCTGCCCCTTCGACAATGCCCTGC	723
	CCT0783 hit2*	TCCCAGGATGAGCGAAGGCGGCAGCGATTTCGCCCCGCCATTTCGTCGCCCGGCGCTCTTTCATTTTCCCCTCCCCGCACCGCTTCCTCTTCGACAGTGCCCTGC	721
	JCM24501	TCCCAGGATGAGCGAAGTCAGCAGCCATTTCGCCCCGCCATTTCGTCGCCCGGAGCTCTTTCATTTTCCCCTCCCCGCACCGCTTGCCCTTCGACAATGCCCTGC	721
	consensus	*!*!***!**!!!!!!!*!*!!!!!**!!!!!!!!!!*!!!!!!!!!!!*!!!*!!!!!!!!!!!!!!!!!!!!!!!!!!*!!!**!*!!!!!!!****!!!!!!	

Clade	Strain	740 750 760 770 780 790 800 810 820 830	
A1-clade strains	CCT0783 hit1*	TCCTTCTGCGCTGTCCGCGTCGGACATTGGCTAGCCTGGGCGCTCCTGATCGAGGGACCGGCCCTAACGCCTTCTCCTCGATAGACGGTCCCTTCAACGTTCCTC	837
	CGMCC2.1609 hit1*	TCCTTCTGCGCTGTCCGCGTCGGACATTGGCTAGCCTGGGCGCTCCTGATCGAGGGACCGGCCCTAACGCCTTCTCCTCGATAGACGGTCCCTTCAACGTTCCTC	836
	MTCC457	TCCTTCTGCGCTGTCCGCGTCGGACATTGGCTAGCCTGGGCGCTCCTGATCGAGGGACCGGCCCTAACGCCTTCTCCTCGATAGACGGTCCCTTCAACGTTCCTC	837
	ATCC10788	TCCTTCTGCGCTGTCCGCGTCGGACATTGGCTAGCCTGGGCGCTCCTGATCGAGGGACCGGCCCTAACGCCTTCTCCTCGATAGACGGTCCCTTCAACGTTCCTC	837
	CBS14	TCCTTCTGCGCTGTCCGCGTCGGACATTGGCTAGCCTGGGCGCTCCTGATCGAGGGACCGGCCCTAACGCCTTCTCCTCGATAGACGGTCCCTTCAACGTTCCTC	837
	Z11	TCCTTCTGCGCTGTCCGCGTCGGACATTGGCTAGCCTGGGCGCTCCTGATCGAGGGACCGGCCCTAACGCCTTCTCCTCGATAGACGGTCCCTTCAACGTTCCTC	837
	IFO0559	TCCTTCTGCGCTGTCCGCGTCGGACATTGGCTAGCCTGGGCGCTCCTGATCGAGGGACCGGCCCTAACGCCTTCTCCTCGATAGACGGTCCCTTCAACGTTCCTC	837
	JCM10020	TCCTTCTGCGCTGTCCGCGTCGGACATTGGCTAGCCTGGGCGCTCCTGATCGAGGGACCGGCCCTAACGCCTTCTCCTCGATAGACGGTCCCTTCAACGTTCCTC	837
	Z1	TCCTTCTGCGCTGTCCGCGTCGGACATTGGCTAGCCTGGGCGCTCCTGATCGAGGGACCGGCCCTAACGCCTTCTCCTCGATAGACGGTCCCTTCAACGTTCCTC	837
	NP11	TCCTTCTGCGCTGTCCGCGTCGGACATTGGCTAGCCTGGGCGCTCCTGATCGAGGGACCGGCCCTAACGCCTTCTCCTCGATAGACGGTCCCTTCAACGTTCCTC	837
	JCM10049	TCCTTCTGCGCTGTCCGCGTCGGACATTGGCTAGCCTGGGCGCTCCTGATCGAGGGACCGGCCCTAACGCCTTCTCCTCGATAGACGGTCCCTTCAACGTTCCTC	837
	CECT1137	TCCTTCTGCGCTGTCCGCGTCGGACATTGGCTAGCCTGGGCGCTCCTGATCGAGGGACCGGCCCTAACGCCTTCTCCTCGATAGACGGTCCCTTCAACGTTCCTC	825
A2-clade strains	BOT-A2	TCCTCCTGCGCTGTCCGCGTCGGACACTGGCTAGCCTGGGCGCTCCTGATCGAGGGACCGGCCCTAACGCCTTCTCCTCGATAGACGGTCCCTTCGACGTTCCTC	827
	NBRC0880	TCCTCCTGCGCTGTCCGCGTCGGACACTGGCTAGCCTGGGCGCTCCTGATCGAGGGACCGGCCCTAACGCCTTCTCCTCGATAGACGGTCCCTTCGACGTTCCTC	827
	ATCC204091	TCCTCCTGCGCTGTCCGCGTCGGACACTGGCTAGCCTGGGCGCTCCTGATCGAGGGACCGGCCCTAACGCCTTCTCCTCGATAGACGGTCCCTTCGACGTTCCTC	827
	delta dao 1e	TCCTCCTGCGCTGTCCGCGTCGGACACTGGCTAGCCTGGGCGCTCCTGATCGAGGGACCGGCCCTAACGCCTTCTCCTCGATAGACGGTCCCTTCGACGTTCCTC	827
	JCM10021	TCCTCCTGCGCTGTCCGCGTCGGACACTGGCTAGCCTGGGCGCTCCTGATCGAGGGACCGGCCCTAACGCCTTCTCCTCGATAGACGGTCCCTTCGACGTTCCTC	827
	ATCC10657	TCCTCCTGCGCTGTCCGCGTCGGACACTGGCTAGCCTGGGCGCTCCTGATCGAGGGACCGGCCCTAACGCCTTCTCCTCGATAGACGGTCCCTTCGACGTTCCTC	827
	CGMCC2.1609 hit2*	TCCTCCTGCGCTGTCCGCGTCGGACACTGGCTAGCCTGGGCGCTCCTGATCGAGGGACCGGCCCTAACGCCTTCTCCTCGATAGACGGTCCCTTCGACGTTCCTC	827
	VN1	TCCTTCTGCGCTGTCCGCGTCGGACACTGGCTAGCCTGGGCGCTCCTGATCGAGGGACCGGCCCTAACGCCTTCTGCTCGATAGACGGTCCT.TCGACGTTCCTC	827
	CCT0783 hit2*	TCCTTCTGCGCTGTCCGTGTCGGACACGGGCTAGCCTGGGCGCTCCTGATCGAGGGACCGGCCTCAACGCCTTCTCCTCGATAGACGGTCCCGTCGACGTTCCTC	826
	JCM24501	TCCTTCTGCGCTGTCCGCGTCGGACTCTGGCTAGCCTGGGCGCTCCTAATCGAGGGACCGGCCCTAACGCCTTCTCCTCGATAGACGGTCCCTTCGACGTTCCTC	826
	consensus	!!!!*!!!!!!!!!!!!*!!!!!!!***!!!!!!!!!!!!!!!!!!!*!!!!!!!!!!!!!!!**!!!!!!!!!!*!!!!!!!!!!!!!!!**!!*!!!!!!!!!	

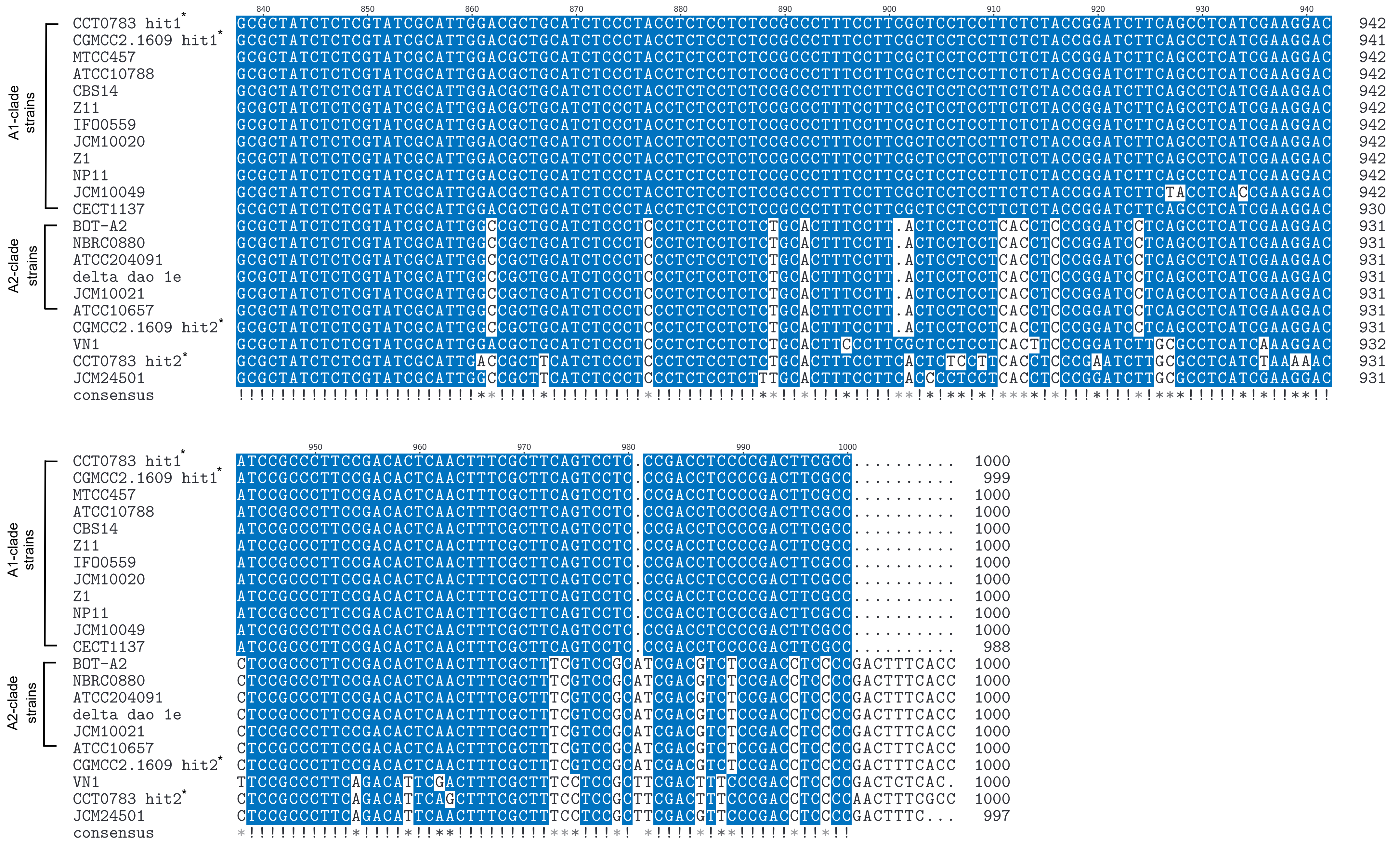
A1-clade strains
A2-clade strains
CCT0783 hit1*
CGMCC2.1609 hit1*
MTCC457
ATCC10788
CBS14
Z11
IFO0559
JCM10020
Z1
NP11
JCM10049
CECT1137
BOT-A2
NBRC0880
ATCC204091
delta dao 1e
JCM10021
ATCC10657
CGMCC2.1609 hit2*
VN1
CCT0783 hit2*
JCM24501
consensus
